# Fecal profiling reveals a common microbial signature for pancreatic cancer in Finnish and Iranian cohorts

**DOI:** 10.1186/s13099-025-00698-0

**Published:** 2025-04-16

**Authors:** Heidelinde Sammallahti, Sama Rezasoltani, Satu Pekkala, Arto Kokkola, Hamid Asadzadeh Agdaei, Mehdi Azizmohammad Looha, Reza Ghanbari, Farhad Zamani, Amir Sadeghi, Virinder Kaur Sarhadi, Marja Tiirola, Pauli Puolakkainen, Sakari Knuutila

**Affiliations:** 1 Department of Pathology, Faculty of Medicine, University of Helsinki, 00014 Helsinki, Finland; 2 Department of Surgery, Abdominal Center, University of Helsinki, Helsinki University Hospital, 00290 Helsinki, Finland; 3 Division of Oral Microbiology and Immunology, Department of Operative Dentistry, Periodontology and Preventive Dentistry, Rheinisch-Westfälische Technische Hochschule (RWTH) University Hospital, 52074 Aachen, Germany; 4 Faculty of Sport and Health Sciences, University of Jyväskylä, 40014 Jyväskylä, Finland; 5 Department of Surgery, University of Helsinki and Helsinki University Hospital, 00290 Helsinki, Finland; 6 Basic and Molecular Epidemiology of Gastrointestinal Disorders Research Center, Research Institute for Gastroenterology and Liver Diseases, Shahid Beheshti University of Medical Sciences, P.O. Box 1985717411, Tehran, Iran; 7 Gene Therapy Research Center, Digestive Diseases Research Institute, Shariati Hospital, Tehran University of Medical Sciences, Tehran, Iran; 8 Gastrointestinal and Liver Diseases Research Center, Iran University of Medical Sciences, Tehran, Iran; 9 Gastroenterology and Liver Diseases Research Center, Shahid Beheshti University of Medical Sciences, Tehran, Iran; 10 Department of Oral and Maxillofacial Diseases, Helsinki University Hospital and University of Helsinki, 00290 Helsinki, Finland; 11 Department of Environmental and Biological Sciences, Nanoscience Center, University of Jyväskylä, 40014 Jyväskylä, Finland; 12 BiopSense Oy, Eeronkatu 10, 40720 Jyväskylä, Finland

**Keywords:** Pancreatic cancer, Gut microbiota, Fecal, 16S rRNA gene sequencing, Microbial profile, Microbial classifier, Butyrate-producing, Clostridia, Population differences, Noninvasive biomarkers

## Abstract

**Background:**

Pancreatic cancer (PC) presents a significant challenge in oncology because of its late-stage diagnosis and limited treatment options. The inadequacy of current screening methods has prompted investigations into stool-based assays and microbial classifiers as potential early detection markers. The gut microbiota composition of PC patients may be influenced by population differences, thereby impacting the accuracy of disease prediction. However, comprehensive profiling of the PC gut microbiota and analysis of these cofactors remain limited. Therefore, we analyzed the stool microbiota of 33 Finnish and 50 Iranian PC patients along with 35 Finnish and 34 Iranian healthy controls using 16S rRNA gene sequencing. We assessed similarities and differences of PC gut microbiota in both populations while considering sociocultural impacts and generated a statistical model for disease prediction based on microbial classifiers. Our aim was to expand the current understanding of the PC gut microbiota, discuss the impact of population differences, and contribute to the development of early PC diagnosis through microbial biomarkers.

**Results:**

Compared with healthy controls, PC patients presented reduced microbial diversity, with discernible microbial profiles influenced by factors such as ethnicity, demographics, and lifestyle. PC was marked by significantly higher abundances of facultative pathogens including Enterobacteriaceae, Enterococcaceae, and Fusobacteriaceae, and significantly lower abundances of beneficial bacteria. In particular, bacteria belonging to the Clostridia class, such as butyrate-producing Lachnospiraceae, Butyricicoccaceae, and Ruminococcaceae, were depleted. A microbial classifier for the prediction of pancreatic ductal adenocarcinoma (PDAC) was developed in the Iranian cohort and evaluated in the Finnish cohort, where it yielded a respectable AUC of 0.88 (95% CI 0.78, 0.97).

**Conclusions:**

This study highlights the potential of gut microbes as biomarkers for noninvasive PC screening and the development of targeted therapies, emphasizing the need for further research to validate these findings in diverse populations. A comprehensive understanding of the role of the gut microbiome in PC could significantly enhance early detection efforts and improve patient outcomes.

**Supplementary Information:**

The online version contains supplementary material available at 10.1186/s13099-025-00698-0.

## Introduction

Despite significant declines in overall cancer mortality in recent decades, pancreatic cancer (PC) remains a formidable challenge [1, 2]. In 2017, PC accounted for 1.8% of new cancer cases worldwide and 4.6% of cancer-related deaths [3]. With higher sociodemographic status linked to this malignancy and as living standards rise in low- and middle-income countries, the global burden of PC is increasing, with death rates projected to nearly double in the next 40 years [3]. Tumor resection, often combined with (neo)adjuvant therapy, is currently the only curative option. However, due to late-onset and nonspecific symptoms, PC is frequently diagnosed at an advanced, unresectable stage [4]. No early detection screening tests are available at present [5, 6]. Current diagnostic methods for PC, including computed tomography and magnetic resonance imaging, are typically employed only after symptom onset [7]. Serum protein carbohydrate antigen 19–9 (CA19-9) is used for disease monitoring but is unsuitable for early screening because of its low sensitivity (79–81%) and low positive predictive value (0.5–0.9%) in symptomatic patients [8, 9]. Various early screening strategies currently under investigation involve biomarkers based on proteins and nucleic acids, such as circulating tumor cells, circulating tumor DNA, microRNAs, and exosomes, in biofluids such as blood, urine, stool, and saliva [10, 11]. Stool-based sampling is particularly promising because it is noninvasive, cost-effective, and can be conveniently performed at home [12]. Differentially abundant gut microbes have been proposed as stool biomarkers [13].

The pancreas, which is connected to the small intestine via the pancreatic ducts, interacts closely with the gut microbiota. Intestinal bacterial metabolites can induce peptide expression in pancreatic β-cells, which in turn can regulate the composition of the gut microbiota [14]. Pancreatic dysfunction due to inflammation or disease may alter these secretions, possibly impacting the composition, diversity, and functions of the gut microbiota [14]. Disrupted homeostasis in microbial communities, termed dysbiosis [15], has been associated with various cancers, particularly those affecting the gastrointestinal tract, such as gastric and colorectal cancer (CRC) [15–19]. Characteristic microbiota profiles have also been identified in PC, both in the gut and other body sites [20]. These profiles may have potential as biomarkers for PC screening and surveillance [13, 21]. Unfortunately, findings in PC remain sparse, sometimes contradictory, and difficult to generalize. Lifestyle, geographic location, and population differences significantly influence the gut microbiota composition [22, 23] and must be considered in microbiome-based biomarker research. However, comprehensive profiling of the PC gut microbiota and analysis of these cofactors remain limited.

This study explored the gut microbiota of PC patients from Finland and Iran. Our objectives were to identify characteristic gut microbiota traits in both populations, assess similarities and differences while considering sociocultural influences, and generate a statistical model for disease prediction based on a panel of microbial markers characteristic of PC in both cohorts. Our aim was to expand the current understanding of the PC gut microbiota, discuss the impact of population differences on the PC microbiota, and contribute to the development of early screening methods for this malignancy.

## Material and methods

### Study population

In this observational case‒control study, we analyzed the stool microbiota of 83 PC patients (33 Finnish and 50 Iranian patients) and 69 healthy controls (HCs, 35 Finnish and 34 Iranian controls) via amplicon sequencing of the bacterial 16S rRNA gene. All patients included in the study were diagnosed with pancreatic ductal adenocarcinoma (PDAC), the most common type of PC. The exclusion criteria for patients and controls were antimicrobial treatment for up to 3 months and treatment for other kinds of cancer for up to 5 years before sample collection.

Among individuals who underwent pancreatic surgery, 53 Finnish patients were recruited at the surgical department of Helsinki University Hospital, Finland, between March 2021 and May 2022. After excluding patients with diagnoses other than PDAC or those who did not meet the criteria, 33 individuals remained. Of these, 12 individuals had received neoadjuvant chemotherapy treatment, and 22 individuals had undergone endoscopic retrograde cholangiopancreatography (ERCP) and biliary stenting before sampling. This was considered during data analysis and interpretation. 35 Finnish HCs were recruited among spouses and acquaintances of the patients, among others. Lifestyle and health-related data of patients and controls were acquired through a questionnaire, and patients’ clinical characteristics were retrieved from the Finnish Patient Data Repository. The Finnish population consisted of 64 Finns and 4 other Europeans of Caucasian ethnicity living in Finland. Participants with Finnish mother tongue and typical Finnish first and last names were considered ethnically Finnish.

**Fig. 1:**
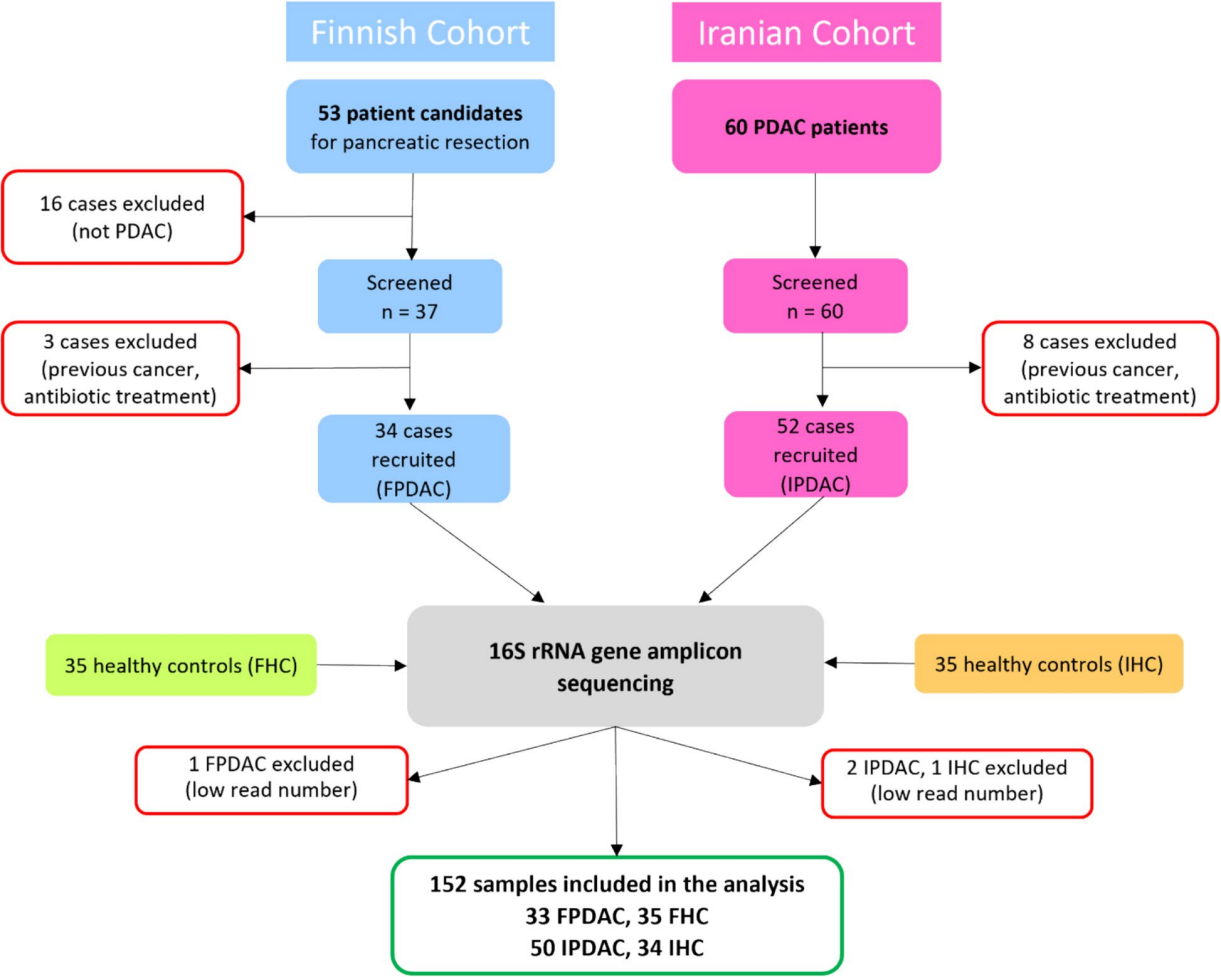
Study design overview and sample sizes. This figure provides a comprehensive overview of the study design, detailing the recruitment and screening process for pancreatic ductal adenocarcinoma (PDAC) patients and healthy controls in the Finnish and Iranian cohorts. FHC, Finnish HC; FPDAC, Finnish PDAC; HC, healthy control; IHC, Iranian HC; IPDAC, Iranian PDAC; PDAC, pancreatic ductal adenocarcinomaa

The Iranian participants were recruited between March and October 2021. Stool samples were collected prior treatment from 60 patients newly diagnosed with PDAC at Taleghani Hospital, Tehran, Iran. After the exclusion of samples that did not meet all eligibility criteria, 50 cases remained. 34 Iranian HCs were included in this study, recruited amongst healthy patient relatives, hospital staff, and healthy individuals visiting the hospital for disease screening. Clinical and health-related data were obtained through a questionnaire*.* The Iranian population consisted of Iranians living in Iran, encompassing various ethnic groups such as Pars, Kurds, Lor, Baluch, and Bakhtiari. In both cohorts, over 60% of patients suffered from comorbidities such as hypertension, hypercholesterolemia, type 2 diabetes mellitus, asthma, coronary artery disease, chronic gastrointestinal inflammation, and rheumatoid arthritis. Likewise, 40–50% HCs suffered from similar medical conditions as the patients, albeit at lower prevalences. All HCs were cancer-free and without any history of cancer. 21% of the Finnish and 6% of the Iranian patients had suffered from previous cancers more than 5 years before participating in this study. The flowchart below (Fig. 1) provides an overview of the study design and the sample sizes, and Table 1 displays the clinical and lifestyle characteristics of the study participants.

**Table 1:** Clinical and lifestyle characteristics of the pancreatic cancer patients and controls included in this study

Variable	Finnish cohort			Iranian cohort		
	PDAC	HC	*p* value^c^	PDAC	HC	*p* value^c^
Samples included (n)	33	35		50	34	
Age^a^ (years)	69 ± 8	65.0 ± 10.3	0.109	62.36 ± 11.9	40.4 ± 11.6	** < 0.001**
Sex (% female)	57.6	60.0	0.839	40.0	52.9	0.242
BMI^a^ (kg/m^2^)	24.8 ± 4.2	26.6 ± 4.4	0.096	NA	NA	
Biliary stent (%)	67.7	0		0	0	
Neoadjuvant treatment (%)	36.4	0		0	0	
Smokers^b^ (%)	40.0	28.6	0.332	32.7	11.8	**0.011**
Alcohol use (%)	63.0	51.4	0.364	3.8	8.8	0.359
Previous cancer	21.2	0		6.0	0	

^a^Given as mean ± standard deviation

^b^Including ex-smokers with smoking cessation < 10 years ago

^c^Significant (< 0.05) *p* values are bolded. Significance was tested by Student’s t-test for continuous variables and Wald H_0_ test for categorical variables. BMI, body mass index; HC, healthy control; NA, not available; PDAC, pancreatic ductal adenocarcinoma

This study was approved by the Ethical Review Board of the Hospital District of Helsinki and Uusimaa, Finland (HUS/1763/2020), and the Clinical Research Ethics Committee of Shahid Beheshti University of Medical Sciences and the Ethics Committee of Taleghani Hospital, Tehran, Iran (IR.SBMU.RIGLD.REC.1398.039). Written informed consent was obtained from all participants before sample collection.

### Stool sample collection, storage, and DNA extraction

The Finnish stool samples were collected into INVITEK^®^ Stool Collection Tubes with DNA Stabilizer (Invitek Molecular, Berlin, Germany) according to the manufacturer’s instructions, stored at + 4 °C for 0–5 days, aliquoted, and preserved at − 20 °C for 1–14 months until further use. After the samples were thawed at room temperature and vortexed, they were prepared for DNA extraction by combining 200 μl of sample suspension with 800 μl of lysis buffer, homogenized by bead beating with 0.5 g × 0.1 mm and 0.1 g × 0.5 mm glass beads (MoBio laboratories, Carlsbad, CA, USA) at 2500 rpm and 4.04 m/s for 2 × 30 s with a Bead Rupture Elite Bead Mill Homogenizer (Omni International by PerkinElmer, Waltham, MA, USA), followed by 5 min of centrifugation at 15,000 ×*g*. The Iranian stool samples were collected in Eppendorf tubes, snap frozen at − 80 °C and stored for 0–9 months until their shipment to Finland on dry ice, where they were stored at − 20 °C for 5 months, due to organizational circumstances. To prepare the Iranian samples for DNA extraction, we attempted to mimic the collection and thawing procedures of the Finnish samples as closely as possible by vortexing 200 mg of frozen stool with 1 ml INVITEK^®^ DNA Stabilizer (Invitek Molecular, Berlin, Germany), combining 200 μl sample suspension with 800 μl lysis buffer, and further homogenizing and handling them in the same way as the Finnish samples described above. Automated DNA extraction was performed simultaneously for all samples on the chemagic™ 360 instrument using the chemagic™ DNA Stool 200 Kit H96 (PerkinElmer, Waltham, MA, USA), following the manufacturer’s instructions. The DNA concentration was spot-checked with the Qubit dsDNA HS Assay Kit and the Qubit 2.0 fluorometer (Thermo Fisher Scientific, Waltham, MA, USA), and DNA quality was assessed with the Agilent 2200 TapeStation and the Genomic DNA Screen Tape Assay (Agilent Technologies, Inc., Santa Clara, CA, USA).

### 16S rRNA gene sequencing

The V4 variable region of the bacterial 16S rRNA gene was amplified using the Earth Microbiome primers 515F-Y [24] and 806R [25] with a protocol adapted from Mäki et al. [26]. In brief, the qPCR mixtures (25 μl) consisted of 1 × Maxima SYBR Green/Fluorescein qPCR Master Mix (Thermo Fisher Scientific, Waltham, MA, USA), 0.4 μM reverse and forward primers and 6 ng of template DNA. The thermal conditions were initial denaturation at 95 °C for 10 min, followed by 30 cycles at 94 °C for 30 s, 52 °C and 72 °C for 60 s, and a final extension at 72 °C for 5 min (C1000 ThermalCycler, Bio-Rad Laboratories, Hercules, CA, USA). Secondary PCR with 10 cycles was conducted using fusion primers IonA-barcode-M13: M13-515F-Y (in 1:0.1 ratio) and P1-806R (linker M13 sequence TGTAAAACGACGGCCAGT) to attach Ion Torrent barcodes and sequencing adaptors to the amplicons, as described by Mäki et al. [26]. The products were purified using the AMPure XP purification system (Beckman Coulter Life Sciences, Indianapolis, IN, USA). Sample concentrations were analyzed using the Qubit dsDNA HS assay (Thermo Fisher Scientific Inc., USA), and samples were pooled in equimolar concentrations. The sample pool was sequenced uni-directionally on the Ion Torrent PGM™ System with the Ion PGM™ Hi-Q View chemistry (Thermo Fisher Scientific Inc., Waltham, MA, USA) in the sequencing facility of the Department of Biological and Environmental Sciences, University of Jyväskylä, Finland.

### Statistical and bioinformatic analysis

#### Statistical analysis

Patient metadata and sequencing results were analyzed with Microsoft Excel (version 2408 build 16.0.17928.20114, Redmond, WA, USA) and IBM SPSS Statistics (version 29.0.1.0, Chicago, IL, USA). Descriptive statistics are presented as percentages or means ± standard deviations. Statistical differences were tested with the Student’s t-test for continuous variables (age, body mass index (BMI)) and the Wald H_0_ test for categorical variables (sex, smoking, alcohol consumption, comorbidities), and *p* values less than 0.05 were considered significant. For power analysis, we calculated post-hoc statistical power (two independent study groups, dichotomous) of the main differential phyla, families, and genera, using relative abundances between patients and controls, with type I/II errors set at α = 0.05.

#### Microbial profiling

The microbial community composition and diversity were analyzed using QIAGEN CLC Genomics Workbench 24.0, CLC Microbial Genomics Module, version 24.0 (Qiagen, Aarhus, Denmark). Raw reads from 16S rRNA gene amplicon sequencing were filtered and trimmed. Reference-based OTU clustering was performed at 97% similarity using the Silva SSU database (version 138.1, 99% full-length sequences) [27] as a reference dataset, with a parameter setting at the best matching result, the minimum number of duplicates for specific read-data set at 2, chimera crossover cost set at 6, and Kmer size set at 2 (see [28] for further details). The group differences in the alpha diversity of the gut microbiota (Shannon entropy, *i.e.*, species diversity, and Chao1 index, *i.e.*, species richness) and phylogenetic diversity were analyzed with the Kruskal–Wallis test. For these diversity analyses, the read depth was rarefied to 5207 reads per sample. The gut microbiota beta diversity analysis was based on the Bray–Curtis distance and PERMANOVA (PERmutational Multivariate ANalysis Of Variance) between the groups, and the results were visualized through principal coordinate analysis (PCoA). In addition to analyzing the main groups, we also tested several covariate groups by PERMANOVA, including age group, sex, BMI, alcohol consumption, smoking, neoadjuvant treatment, and biliary stenting. After removing chloroplasts and mitochondria and filtering out OTUs present in fewer than 5 samples, we performed differential abundance analysis (DAA) via the generalized linear model (GLM), which assumes that abundances follow a negative binomial distribution, as described [29]. We used the default parameter settings and added corrections for the covariates age group, sex, and smoking. This was followed by Benjamini–Hochberg correction for multiple testing. Statistical significance was set at an FDR (false discovery rate) of *p* < 0.05. To obtain a better overview of the differentially abundant features, we generated subtables of the OTU abundance table, grouped them according to the taxonomic levels of phylum, class, family, and genus, and analyzed them once more with GLM, using the same settings and corrections as described above. Since certain Clostridiales strains have been shown to mediate anticancer immune responses [30], we paid particular attention to families and genera belonging to the class Clostridia, especially those with lower abundance in PDAC. The main results were visualized in Venn diagrams using CLC Microbial Genomics Module and in bar diagrams and heatmaps using Microsoft Excel. To further explore and visualize the taxonomic differences between the groups, we used linear discriminant analysis effect size (LEfSe) [31]. An LDA (linear discriminant analysis) score of > 4 and an alpha *p* value of < 0.01 were considered significant.

#### Gut microbial function prediction and pathway analysis

The Kyoto Encyclopedia of Genes and Genomes (KEGG) functions of the OTUs were predicted using CLC Microbial Genomics and Phylogenetic Investigation of Communities by Reconstruction of Unobserved States (PICRUSt) software [32]. The group differences of these predicted functions were then explored in MicrobiomeAnalyst surroundings [33], using low count filtering (minimum count 4 and 20% prevalence in samples), low variance filtering with an interquartile range, and cumulative sum scaling. Differential abundance analysis of the predicted KO (KEGG Orthology)-term features was performed with Microbiome Multivariable Association using Linear Models2 (MaAsLin2 [34]) and LEfSe, with the statistical significance set at FDR *p* < 0.05. In addition, we performed a pathway analysis in MicrobiomeAnalyst to identify the KEGG pathways associated with the differential predicted functions. The results were visualized through principal component analysis (PCA) and LEfSe.

#### Statistical modeling for the prediction of PC, and model evaluation

This part of the analysis was performed in R (version 4.3.1) [35], and the statistical significance was set at *p* < 0.05. For the development of a statistical model for PC prediction, we used the larger Iranian cohort (34 patients and 50 controls) as the training data and the smaller Finnish cohort (33 patients and 35 controls) for external validation to assess the robustness and generalizability of the models across a geographically and ethnically distinct population. To generate a microbial classifier for PC prediction, microbial taxa (referred to as “variables”) on phylum, family, and genus levels were selected by two complementary approaches: random forest (RF) and logistic regression (LR). RF was used to rank variables based on their contribution to model performance, as measured by the Mean Decrease GINI (MDG) [36], which included a tenfold cross-validation tuning of the RF parameters with ten iterations. Concurrently, univariate LR was conducted to evaluate the association of each variable with the outcome, followed by multivariate LR to assess combined effects and adjust for confounding factors. Variables were selected based on their contribution to the model’s area under the curve (AUC). The selected variables from both methods were then combined to develop multiple models, employing machine learning (ML) algorithms that included logistic regression (LR), naïve Bayes (NB), support vector machines (SVMs), neural network (NN), and decision trees (DTs) [37–39]. To increase the precision of disease prediction, each ML algorithm was subjected to a fine-tuning process consisting of fivefold cross-validation with ten iterations. For determining the best predictive performance, each model was then evaluated in the Finnish cohort by the area under the receiver operating characteristic (ROC) curve, followed by sensitivity (SE), specificity (SP), positive predictive value (PPV), negative predictive value (NPV), and accuracy (ACC). As recommended by Hosmer et al*.*, an AUC of 0.5 implied a lack of discrimination (*i.e.*, the ability to distinguish patients with or without disease), an AUC of 0.7–0.8 was acceptable, 0.8–0.9 was excellent, and > 0.9 was considered exceptional [40].

## Results

### 16S rRNA gene amplicon sequencing statistics and post-hoc power calculations

After trimming and quality filtering, 1,923,005 reads were analyzed, with an average of 12,651 reads per sample (range: 6904–50,392, SD: 4566). To determine statistical power, we performed post-hoc power calculations with the relative abundances of the main differentially abundant taxa. Due to our rather small sample size, the highest power values were around 30%. For details on sequencing statistics and power calculations see Table S1 (Additional file 1).

### Alpha diversity is decreased in the PC gut microbiota

**Fig. 2:**
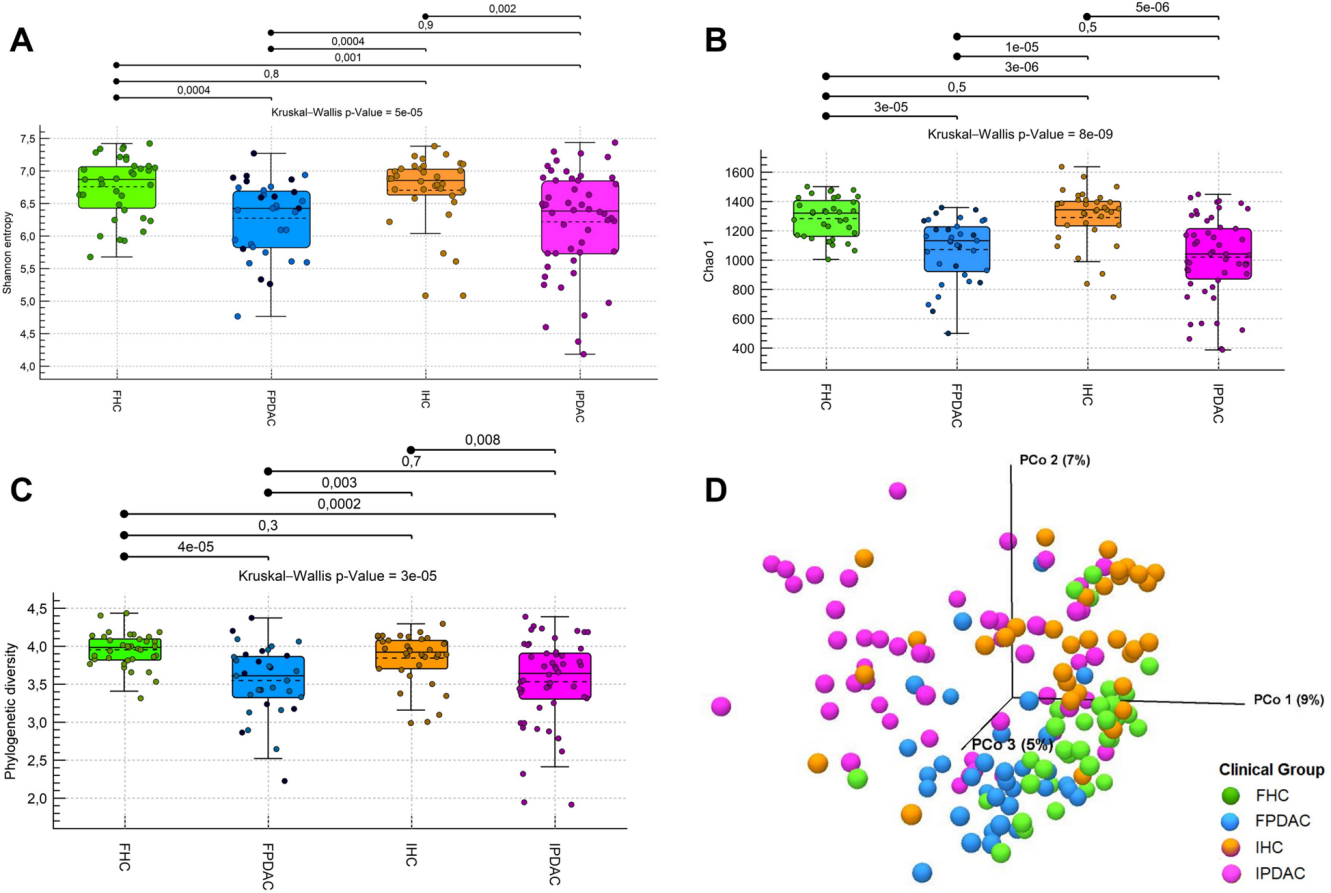
Gut microbiota diversities of Finnish and Iranian PDAC patients and healthy controls. **A** Alpha diversity: Shannon entropy (*i.e.*, species diversity), **B** alpha diversity: Chao1 index (*i.e.*, species richness), and **C** phylogenetic diversity. **D** Beta diversity: Principal coordinate (PCo) plot of the Bray–Curtis distance. FHC, Finnish HC; FPDAC, Finnish PDAC; HC, healthy control; IHC, Iranian HC; IPDAC, Iranian PDAC; PDAC, pancreatic ductal adenocarcinoma

Finnish and Iranian PDAC patients presented significantly lower alpha diversity indices than healthy controls did. The Shannon entropy, Chao 1 index, and phylogenetic diversity were significantly reduced in PDAC patients within both populations (Fig. 2A–C). Additionally, in the integrated dataset, when the Finnish and Iranian groups were combined, PCs presented significantly lower alpha diversity than HCs did (Figure S1A, see Additional file 2). However, comparisons between the individual groups (FPDAC vs. IPDAC and FHC vs. IHC, Fig. 2A–C) and between the populations (All Finns vs. all Iranians, Figure S1B, Additional file 2) revealed no significant differences.

The testing of covariates indicated no significant impact of age, alcohol consumption, biliary stenting, neoadjuvant treatment, sex, or smoking on microbial diversity in the Finnish cohort (Figures S1C-S1E, S1G-S1I; see Additional file 2). Nevertheless, obese Finns had significantly lower species richness (Chao 1) than did normal-weight individuals (Figure S1F, Additional file 2). In the Iranian cohort, the covariates smoking and age had significant impacts on alpha diversity. Both the phylogenetic diversity and the species richness (Chao 1) in individuals younger than 40 years were significantly greater than those in individuals between 40 and 60 years of age and those over 60 years of age, respectively (Figures S1J and S1M, Additional file 2). In addition, both the Shannon entropy and Chao 1 index were significantly lower in smokers than in nonsmokers.

### Microbial community composition varies between the groups

**Fig. 3:**
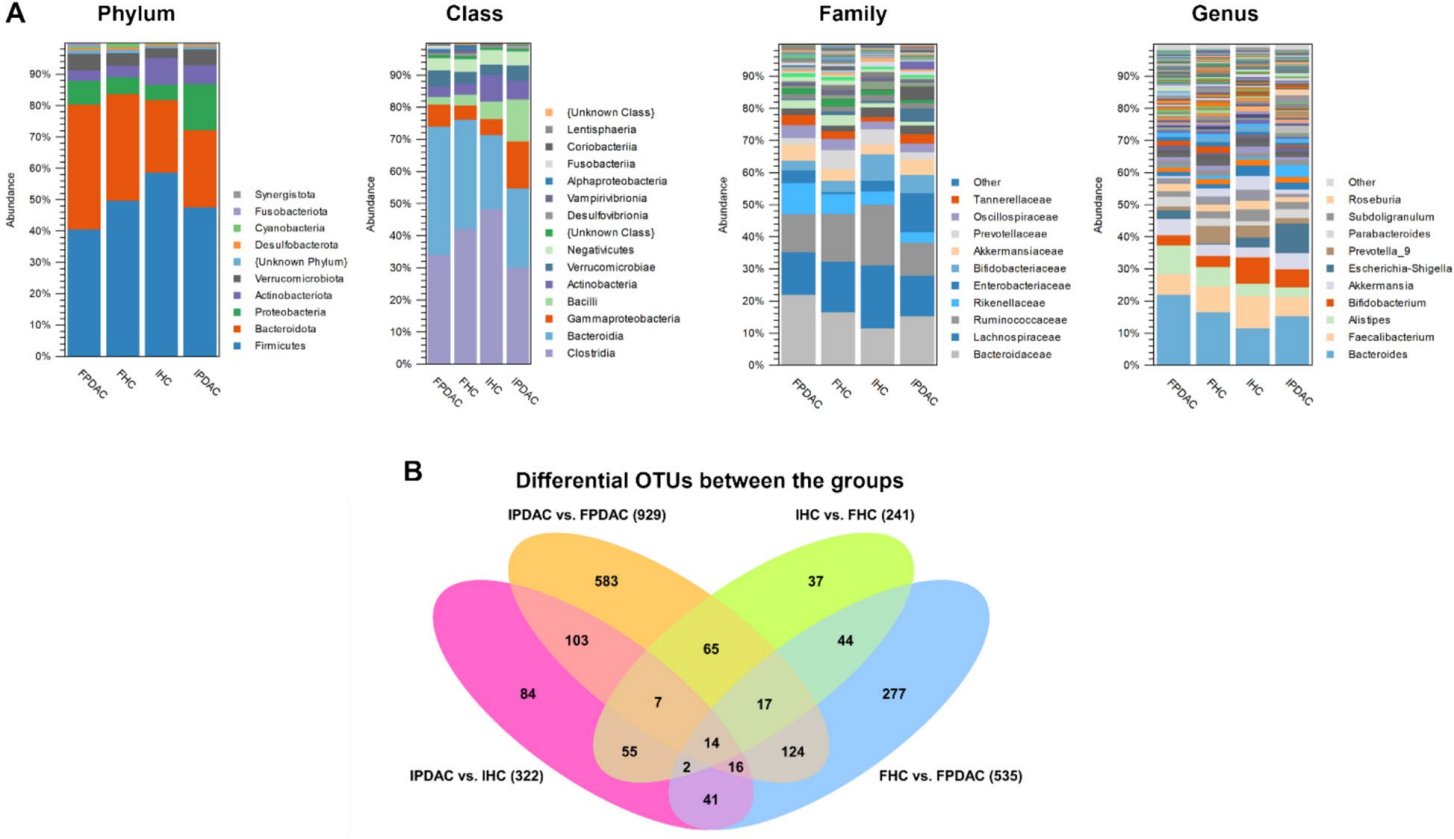
Gut microbiota composition and differentially abundant OTUs. **A** Average composition of the gut microbiota (relative abundance) of FPDAC (n = 33), FHC (n = 35), IPDAC (n = 50), and IHC (n = 34) at the phylum, class, family, and genus levels, with the most abundant taxa listed. **B** Venn diagram visualizing unique and shared OTUs between the groups. The bracketed numbers refer to the differentially abundant OTUs in each group comparison. FHC, Finnish HC; FPDAC, Finnish PDAC; HC, healthy control; IHC, Iranian HC; IPDAC, Iranian PDAC; PDAC, pancreatic ductal adenocarcinoma

A total of 16,343 OTUs were identified and assigned to 15 phyla, 26 classes, 110 families, and 348 genera. The average community composition at different taxonomic levels is presented in Fig. 3A and Table S2, Additional file 3.

The relative abundance of the main phyla in the Finnish PDAC gut microbiota was 41% Firmicutes, followed by 40% Bacteroidota, 8% Proteobacteria, 5% Verrucomicrobiota, 3% Actinobacteriota, and 1% Fusobacteriota. Compared to that, Finnish HCs had higher Firmicutes (50%), lower Bacteroidota (34%), Proteobacteria (5%), Verrucomicrobiota (4%), and Fusobacteriota (< 0.01%) but higher Actinobacteriota (4%) relative abundances. In contrast, the relative abundances of the Iranian PDAC gut microbiota were 48% Firmicutes, 25% Bacteroidota, 15% Proteobacteria, 6% Actinobacteriota, and 5% Verrucomicrobiota, and their respective controls had higher Firmicutes (59%), lower Bacteroidota (23%), Proteobacteria (5%), and Verrucomicrobiota (3%), and higher Actinobacteriota (9%) relative abundances. The top ten genera in Finnish PDAC patients were *Bacteroides, Alistipes, Faecalibacterium*, *Akkermansia*, *Parabacteroides*, *Bifidobacterium, Escherichia-Shigella*, *Roseburia*, *Ruminococcus*, and *Subdoligranulum*. In Iranian PDAC patients, they were *Bacteroides, Faecalibacterium*, *Bifidobacterium*, *Agathobacter, Prevotella_9, Subdoligranulum*, *Alistipes, Ruminococcus*, *Blautia*, and *Akkermansia*. See Table S3, Additional file 3, for details.

### Gut microbial beta diversity differs significantly between pancreatic cancer patients and healthy controls and between Finns and Iranians

The PCo plot of the Bray‒Curtis distances clearly separated the clinical groups (FPDAC, FHC, IPDAC, and IHC) by cancer status and population (Fig. 2D). PERMANOVA confirmed significant differences in microbial community composition between patients and controls within and between cohorts (*p* < 0.0001), with varying magnitudes of differences (Table 2).

**Table 2:** Beta diversity analysis via PERMANOVA

Variable	Groups	Group comparisons	pseudo-F statistic^a^	*p* value^b^	*p* value^b^ (Bonferroni)
Clinical groups A	FPDAC, FHC, IPDAC, IHC	FPDAC vs. FHC	2.37449	< 0.0001	< 0.0001
		IPDAC vs. IHC	5.13292	< 0.0001	< 0.0001
		IPDAC vs. FPDAC	3.88052	< 0.0001	< 0.0001
		IHC vs. FHC	3.82313	< 0.0001	< 0.0001
Clinical groups B	PDAC (FPDAC + IPDAC), HC (FHC + IHC)	PDAC vs. HC	5.82668	< 0.0001	< 0.0001
Population origin	All Finnish (FPDAC + FHC), All Iranian (IPDAC + IHC)	All Finnish vs. All Iranian	5.91189	< 0.0001	< 0.0001

^a^pseudo-F is a measure of effect size. The larger it is, the greater the difference in the respective comparison

^b^*p* values < 0.05 indicate significant differences in the average community compositions of the compared groups

FHC, Finnish HC; FPDAC, Finnish PDAC; HC, healthy control; IHC, Iranian HC; IPDAC, Iranian PDAC; PDAC, pancreatic ductal adenocarcinoma

The analysis of covariates by PERMANOVA revealed no significant differences between treated and untreated patients or between those with and without biliary stents in the Finnish cohort. In the Iranian cohort, significant differences were detected between age groups, sexes, and smoking statuses (Table S3, Additional file 4).

### The PC gut microbiota has a distinct profile in both populations

#### Overlapping and distinct taxa in PDAC across Finnish and Iranian cohorts

Statistical comparisons between PDAC patients and HCs yielded 535 differing OTUs in the Finnish cohort and 322 differing OTUs in the Iranian cohort. When comparing Finnish and Iranian PDAC patients, 929 OTUs differed, whereas 241 OTUs varied between the HCs of both cohorts (Fig. 3B).

#### Phylum-level differences

**Fig. 4:**
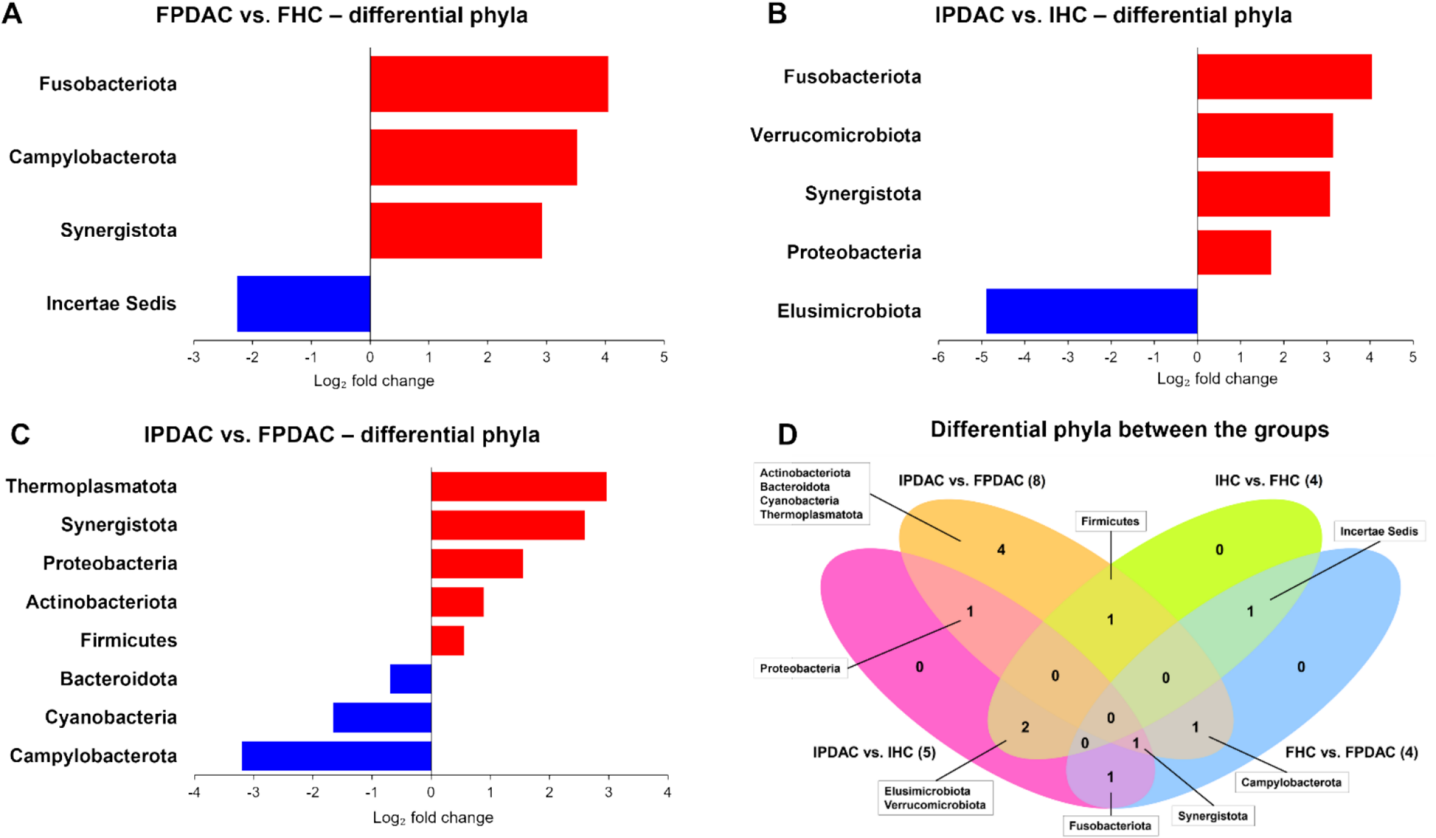
Differentially abundant phyla. Phylum-level differences in the gut microbiota between **A** Finnish FPCAC patients (n = 33) and controls (n = 35), **B** Iranian FPDAC patients (n = 50) and controls (n = 34), and **C** Finnish and Iranian patients. **D** Venn diagram of the differentially abundant phyla between the groups. FHC, Finnish HC; FPDAC, Finnish PDAC; HC, healthy control; IHC, Iranian HC; IPDAC, Iranian PDAC; PDAC, pancreatic ductal adenocarcinoma

Compared with their respective HCs, PDAC patients in both populations presented significantly greater abundances of Fusobacteriota and Synergistota. Additionally, Iranian PDAC patients vs. HCs had higher abundances of Verrucomicrobiota and Proteobacteria and a lower abundance of Elusimicrobiota, whereas Finnish patients had a greater abundance of Campylobacterota than their respective HCs (Fig. 4A, B, D; Table S4; see Additional File 5).

#### Family-level differences

**Fig. 5:**
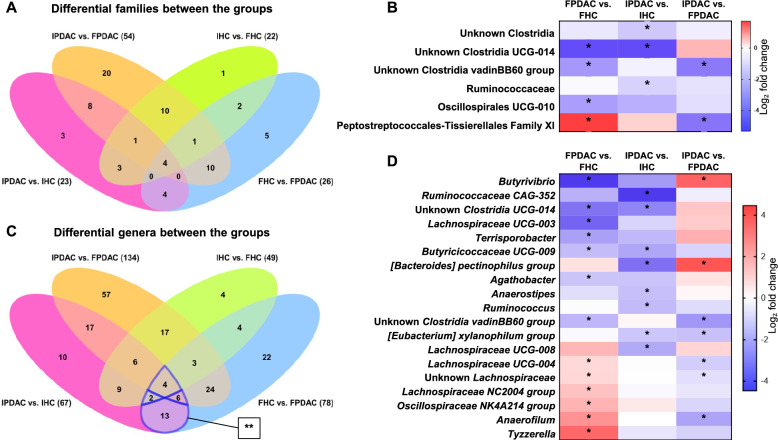
Differentially abundant families and genera. **A** Venn diagram of differentially abundant families between the groups. **B** Heatmap of differentially abundant families belonging to the class Clostridia. **C** Venn diagram of differentially abundant genera between the groups. **D** Heatmap of selected differentially abundant genera belonging to the class Clostridia. *FDR* p* < 0.05. **25 overlapping differential genera in PDAC common to both populations are shown in Table 3. FHC, Finnish HC; FPDAC, Finnish PDAC; HC, healthy control; IHC, Iranian HC; IPDAC, Iranian PDAC; PDAC, pancreatic ductal adenocarcinoma

DAA identified 26 families that differed between patients and controls in the Finnish cohort and 23 in the Iranian cohort (Fig. 5A). Families with significantly higher abundance in PDAC patients in both cohorts included Enterococcaceae, Fusobacteriaceae, and Enterobacteriaceae. In addition, Finnish PDAC patients presented higher abundances of Yersiniaceae, Hafniaceae, and Campylobacteraceae, whereas Iranian PDAC patients presented higher levels of Lactobacillaceae, Akkermansiaceae, and Streptococcaceae compared to their respective HCs, among others. Families with lower abundance in PDAC patients included Clostridia UCG-014, Butyricicoccaceae UCG-009, and Bacilli RF39 in both cohorts; Succinivibrionaceae, Clostridia vadinBB60, and VadinBE97 in Finnish PDAC patients; and unknown Gastranaerophilales, Muribaculaceae, and Ruminococcaceae in Iranian PDAC patients (Table S4, Additional file 5). The heatmap of fold differences in Fig. 5B illustrates that several families belonging to the class Clostridia were present at lower abundances in PC in both populations.

#### Genus-level differences

At the genus level, we detected 78 taxa in the Finnish and 67 taxa in the Iranian cohort, which were differentially abundant between patients and controls (Fig. 5C). Among these, 25 taxa overlapped, with 13 genera unique to PDAC and 12 shared with other comparisons (Fig. 5C and Table 3). The most abundant genera in PDAC in both populations included *Enterococcus, Sellimonas, Veillonella, Klebsiella, Hungatella, Eisenbergiella, Fusobacterium, Enterobacter, Flavonifractor,* and *Coprobacillus*. Genera with lower abundance common to both populations were *Asteroleplasma, Clostridia UCG-014, and Butyricicoccaceae UCG-009.* Notably, *Succinivibrio* had a greater abundance in Iranian PDAC patients and a lower abundance in Finnish PDAC patients, and vice versa, the *Rikenellaceae RC9* gut group had a greater abundance in FPDAC patients but a lower abundance in IPDAC patients. Highly enriched genera unique to Finnish patients included *Serratia, Succiniclasticum, Citrobacter,* and *Hafnia*, and highly depleted genera included *Butyrivibrio, Alloprevotella, Lachnospiraceae UCG-003,* and *Mailhella*. In Iranian patients, *Limosilactobacillus*, *Lactobacillus, Pseudomonas,* and *Acidaminococcus* were notably more abundant, whereas *Muribaculaceae CAG-873*, *Elusimicrobium, [Bacteroides] pectinophilus group, and Lycinibacillus* were notably depleted (Table S4, see Additional file 5). The differential abundance heatmap in Fig. 5D highlights selected Clostridia genera with lower abundance in PDAC patients compared to HCs, in one or both cohorts. These patterns illustrate the consistent decline in key butyrate-producing Clostridia across different populations in PDAC.

**Table 3:** Differentially abundant genera in PDAC common to both populations

	Genus^a^	Change^b^	Taxonomy (phylum/class)	Occurrence^c^
1	*Sellimonas*	+	Firmicutes/Clostridia	Unique to PDAC vs. HC in both populations
2	*Veillonella*	+	Firmicutes/Negativicutes	
3	*Klebsiella*	+	Proteobacteria/Gammaproteobacteria	
4	*Eisenbergiella*	+	Firmicutes/Clostridia	
5	*Fusobacterium*	+	Fusobacteriota/Fusobacteriia	
6	*Enterobacter*	+	Proteobacteria/Gammaproteobacteria	
7	Unknown Enterobacterales	+	Proteobacteria/Gammaproteobacteria	
8	*[Clostridium] innocuum group*	+	Firmicutes/Clostridia	
9	Unknown Enterobacteriaceae	+	Proteobacteria/Gammaproteobacteria	
10	*Anaerotruncus*	+	Firmicutes/Clostridia	
11	*Lachnoclostridium*	+	Firmicutes/Clostridia	
12	*Butyricicoccaceae UCG-009*	−	Firmicutes/Clostridia	
13	*Clostridia UCG-014*	−	Firmicutes/Clostridia	
14	*Coprobacillus*	+	Firmicutes/Bacilli	In PDAC vs. HC in both populations In IPDAC vs. FPDAC
15	*Cloacibacillus*	+	Synergistota/Synergistia	
16	Unknown Gammaproteobacteria	+	Proteobacteria/Gammaproteobacteria	
17	*Kluyvera*	+	Proteobacteria/Gammaproteobacteria	
18	*Bacteroides*	+	Bacteroidota/Bacteroidia	
19	*Asteroleplasma*	−	Firmicutes/Bacilli	
20	*Enterococcus*	+	Firmicutes/Bacilli	In PDAC vs. HC in both populations In IPDAC vs. FPDAC In IHC vs. FHC
21	*Erwinia*	+	Proteobacteria/Gammaproteobacteria	
22	*Flavonifractor*	+	Firmicutes/Clostridia	
23	*Succinivibrio*	+ / −	Proteobacteria/Gammaproteobacteria	
24	*Hungatella*	+	Firmicutes/Clostridia	In PDAC vs. HC in both populations In IHC vs. FHC
25	*Rikenellaceae RC9 gut group*	+ / −	Bacteroidota/Bacteroidia	

^a^In descending order of fold differences

^b^Indicates significantly higher (+) or lower (−) abundance in PDAC in both populations

^c^See also Venn diagram, Fig. 5C

FHC, Finnish HC; FPDAC, Finnish PDAC; HC, healthy control; IHC, Iranian HC; IPDAC, Iranian PDAC; PDAC, pancreatic ductal adenocarcinoma

#### Differences between the populations

The comparison between Finnish and Iranian PDAC patients revealed several distinct differences. At the phylum level, Iranian PC vs. Finnish PC presented significantly greater abundances of Thermoplasmatota, Synergistota, Proteobacteria, Actinobacteriota, and Firmicutes (Fig. 4C). At the family level, we observed significantly greater abundances of the facultative pathogens Streptococcaceae, Enterococcaceae, and Lactobacillaceae of the Lactobacillales order and several Gammaproteobacteria families, such as Pseudomonadaceae, Xanthomonadaceae, and Enterobacteriaceae, the latter of which includes the facultative pathogen *E. coli* (Table S4, Additional file 5)*.* Conversely, Finnish PDAC patients showed significant enrichment of the phyla Campylobacteriota, Cyanobacteria, and Bacteroidota; the families Bacteroidaceae, Barnesiellaceae, and Rikenellaceae within the Bacteroidia class; Monoglobaceae, Clostridia vadinBB60 group, and Family XI within the Clostridia class; and Helicobacteraceae and Campylobacteraceae within the Campylobacteria class, among others (Table S4, Additional file 5).

#### Cohortwise biomarker potential

**Fig. 6:**
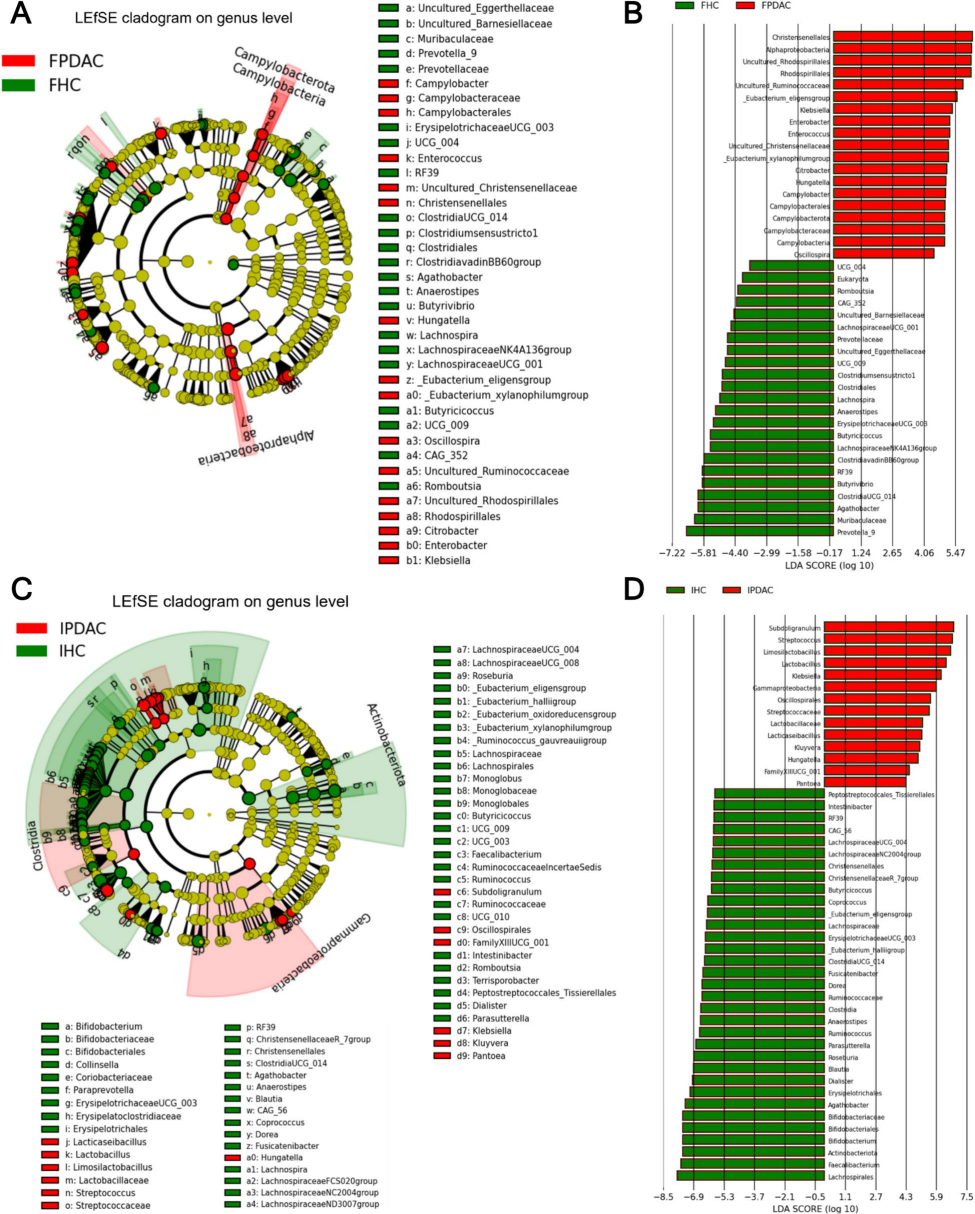
Major differential taxonomic features between PDAC patients and healthy controls visualized via a linear discriminant analysis (LDA) effect size (LEfSe) cladogram and histogram. The cladogram shows the phylogenetic relationships of differentially abundant taxa for the PDAC groups (red) with their controls (green). The size of the nodes is proportional to the taxon’s abundance. In the histogram, positive LDA scores indicate enrichment of taxa in the PDAC groups (red) relative to healthy controls (green), and negative LDA scores indicate depletion of the respective taxa. **A** LEfSe cladogram and **B** LEfSe histogram of FPDAC vs. FHC. **C** LEfSe cladogram and **D** LEfSe histogram of IPDAC vs. IHC. Kruskal–Wallis sum rank test, *p* < 0.01, LDA scores (log10) > 4. FPDAC, Finnish PDAC; FHC, Finnish HC; IPDAC, Iranian PDAC; IHC, Iranian HC; HC, healthy control; PDAC, pancreatic ductal adenocarcinoma

In addition to differential abundance analysis, we assessed the biomarker potential of differential taxa using LEfSe, which evaluates biological consistency and effect size. Both populations' PDAC samples were enriched in *Klebsiella* and *Hungatella* and depleted of *Agathobacter*, *Anaerostipes*, and *Clostridia*. Finnish PDAC samples were furthermore enriched in Christensenellales, Rhodospirillales, *Enterobacter, Enterococcus, Citrobacter, Campylobacter*, and *Oscillospira* and depleted in *Prevotella_9, Butyrivibrio, Butyricicoccus, Lachnospira, and Romboutsia,* among others (Fig. 6A, B). Iranian PDAC samples, on the other hand, were enriched in *Subdoligranulum, Streptococcus, Lactobacillus, Limosilactobacillus, Kluyvera,* and *Pantotea* and depleted in *Faecalibacterium, Bifidobacterium, Dialister, Blautia, Roseburia, Parasutterella,* and *Ruminococcus,* among others (Fig. 6C, D).

### Predicted functions of PC gut microbes and their pathway analysis further underline the differences between the populations

**Fig. 7:**
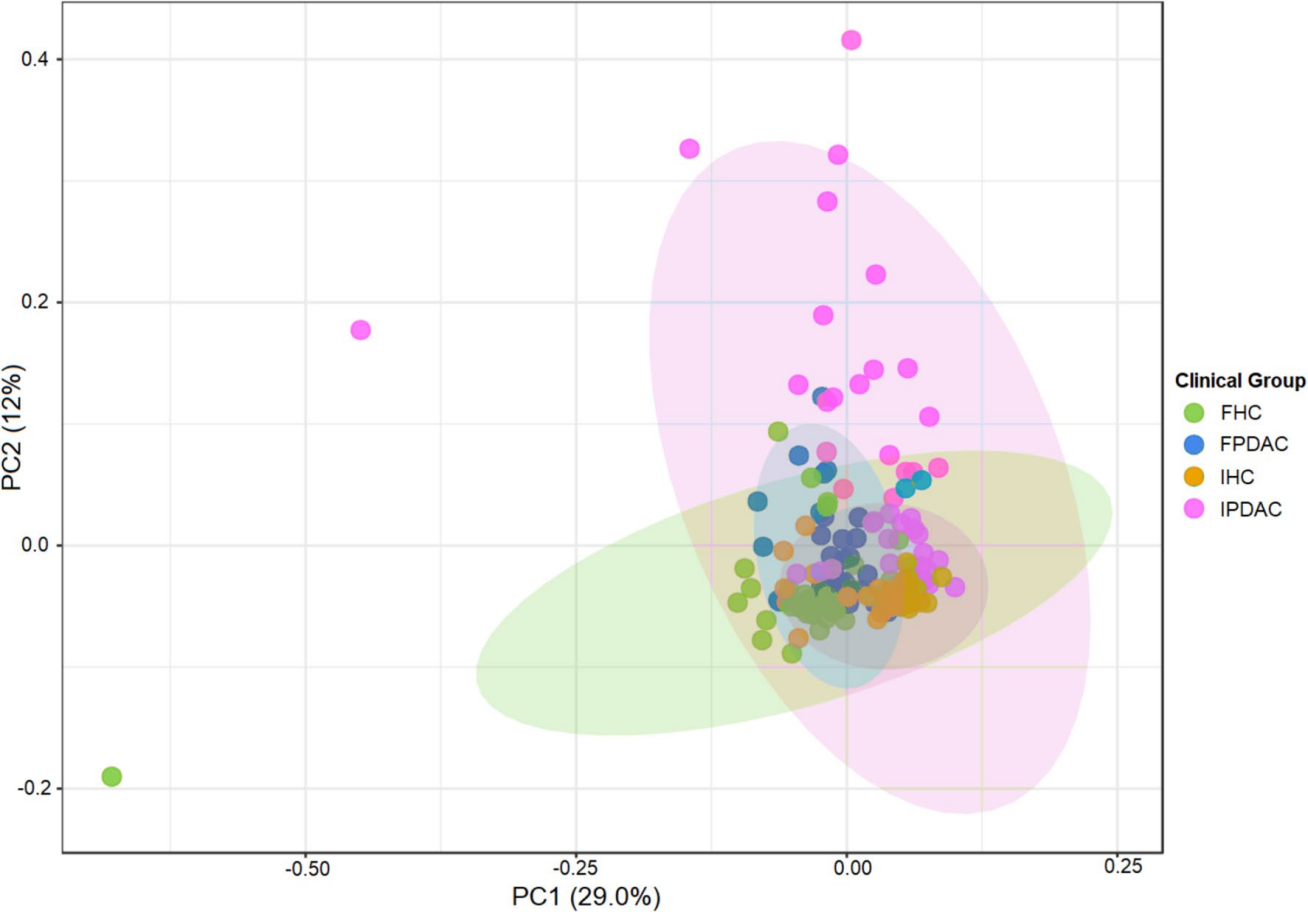
Principal component analysis (PCA) plot of predicted microbial functions grouped according to clinical status and cohort. FPDAC, Finnish PDAC; FHC, Finnish HC; IPDAC, Iranian PDAC; IHC, Iranian HC; HC, healthy control; PDAC, pancreatic ductal adenocarcinoma

**Fig. 8:**
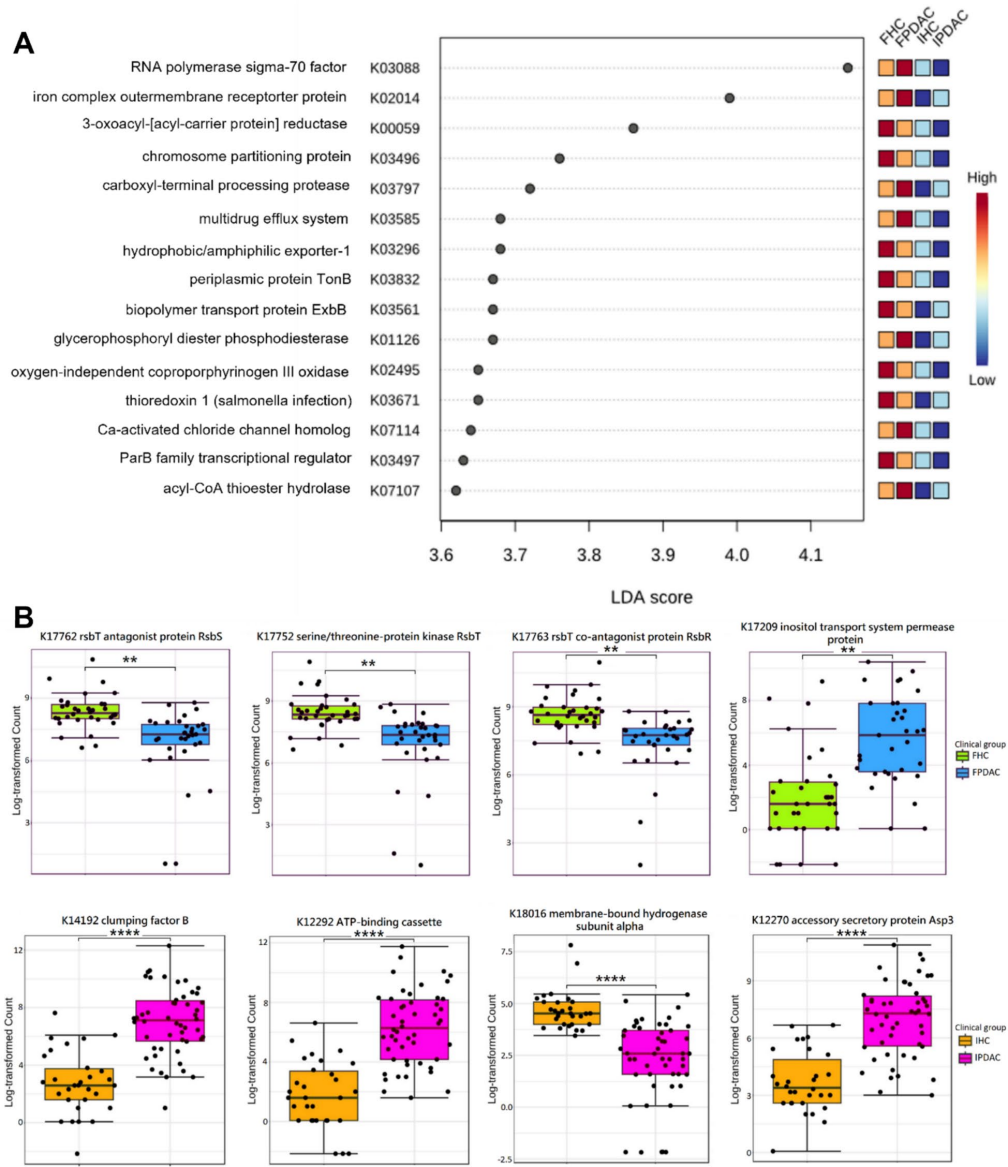
Function prediction. **A** Linear discriminant analysis (LDA) effect size (LEfSe) analysis showing the 15 functions with the greatest differences between PDAC patients and HCs. An LDA score > 3.5 and a false discovery rate (FDR)-corrected *p* value < 0.05 were considered significant. **B** Log-transformed counts of the top 4 differing predicted functions for FPDAC vs. FHC and IPDAC vs. IHC. Significance was tested using the Mann–Whitney U test with FDR correction, *p < 0.05, **p < 0.01, ***p < 0.001, ****p < 0.0001. Annotations of the KO terms were retrieved from the Kyoto Encyclopedia of Genes and Genomes (KEGG) database. FPDAC, Finnish pancreatic ductal adenocarcinoma (PDAC); FHC, Finnish healthy control; IPDAC, Iranian PDAC; IHC, Iranian healthy control

For the prediction of Kyoto Encyclopedia of Genes and Genomes (KEGG) functions, we identified 8,598 KEGG orthology (KO) term features, of which 6417 features remained after filtering. Among these, 872 KOs in Finnish, and 2049 KOs in Iranian PC patients were significantly different from their respective HCs (FDR < 0.05). Among the 500 most distinctive predicted microbial functions in PC, only 40 overlapped between the populations (Table S5, Additional file 6). Visualisation via a PCA plot (Fig. 7) suggests that particularly Iranian cancer patients differed from the other groups. The LEfSe analysis in Fig. 8A displays the top 15 differing predicted functions between the groups, clearly illustrating the divergences between patients and controls and between the populations.

Figure 8B highlights the top four differing predicted functions in PDAC patients versus HCs. Clumping factor B (K14192), accessory secretory protein Asp3 (K12270), and ATP-binding cassette subfamily C (K12292) were highly enriched in Iranian patients, and inositol transport system permease protein (K17209) was highly enriched in Finnish patients. Conversely, highly depleted predicted functions included the rsbT antagonist protein RsbS (K17762), serine/threonine-protein kinase RsbT (K17752), and rsbT coantagonist protein RsbR (K17763) in Finnish, and membrane-bound hydrogenase subunit alpha (K18016) in Iranian patients. All differential predicted functions are detailed in Table S5 (see Additional file 6).

Subsequent pathway analysis revealed that differentially expressed predicted KOs involved in benzoate degradation, toluene degradation, and carbon fixation pathways in prokaryotes were significantly enriched in Finnish patients. In Iranian patients, significantly enriched differentially expressed predicted KOs were involved in peptidoglycan biosynthesis, galactose metabolism, lysine biosynthesis, and furfural degradation pathways (FDR-corrected *p* < 0.05, Table 4 and Table S5, Additional file 6).

**Table 4:** Pathway analysis of the predicted gut microbial functions of Finnish and Iranian PDAC patients

	Total^a^	Hits^b^	*p* value	FDR
Predicted functions FPDAC				
Benzoate degradation	86	15	3.30E−06	0.000495
Toluene degradation	36	9	1.68E−05	0.00126
Carbon fixation pathways in prokaryotes	97	14	6.66E−05	0.00333
Predicted functions IPDAC				
Peptidoglycan biosynthesis	49	13	2.83E−09	4.25E−07
Galactose metabolism	58	10	1.49E−05	0.00112
Lysine biosynthesis	41	7	0.000327	0.0163
Furfural degradation	6	3	0.000665	0.0249

^a^Number of functions in the respective pathway

^b^Number of differential functions that map into the respective pathway

FDR, false discovery rate-corrected *p* value; FPDAC, Finnish PDAC; IPDAC, Iranian PDAC; PDAC, pancreatic ductal adenocarcinoma

### Statistical modeling for the prediction of PC in the Iranian cohort, and prediction performance testing in the Finnish cohort

#### Variable selection methods and model comparison

**Fig. 9:**
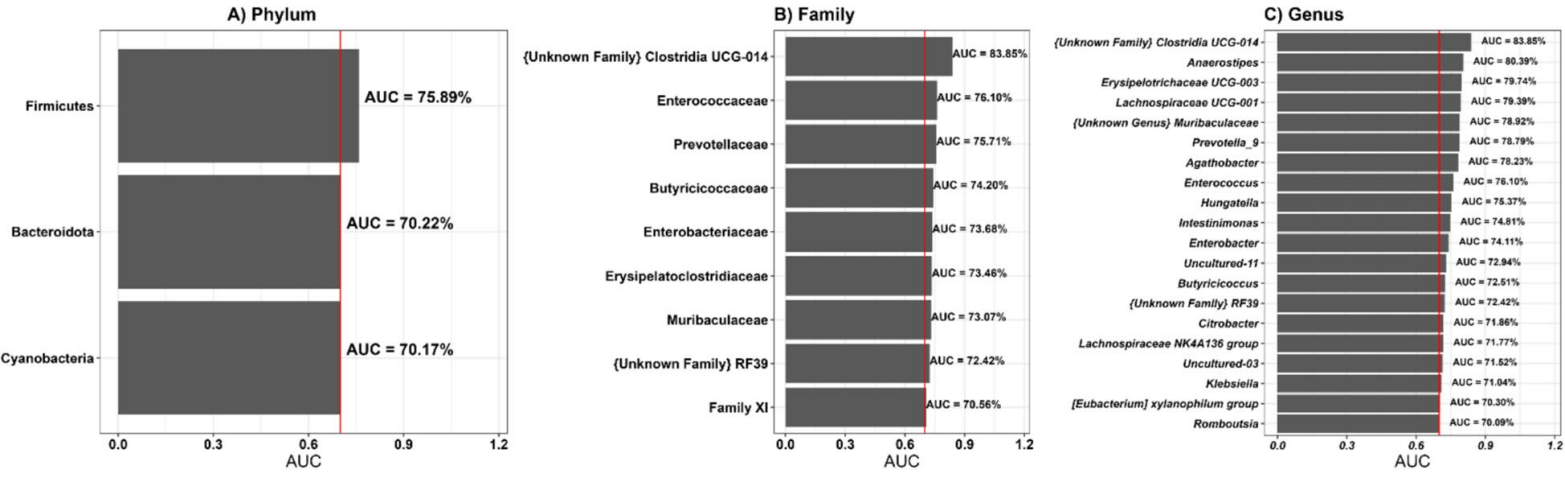
Variable importance analysis for pancreatic ductal adenocarcinoma (PDAC) prediction using Area Under the Curve (AUC) across the taxonomic ranks phylum, family, and genus

For generating a PDAC classifier within the Iranian cohort, microbial taxa at phylum, family, and genus ranks were analysed by Mean Decrease GINI (MDG) using the random forest (RF) method (Figure S2, Additional file 7), and logistic regression (LR) based on AUC values > 0.7 (Table S6, Additional file 8). The resulting variables (*i.e.*, microbial taxa) were used to build predictive models, of which LR demonstrated the highest performance (Table S7, Additional file 9). In the LR model, PDAC-predicting variables included Firmicutes, Bacteroidota, and Cyanobacteria at the phylum level, {Unknown Family} Clostridia UCG-014, Enterococcaceae, Prevotellaceae, Butyricicoccaceae, Enterobacteriaceae, Erysipelatoclostridiaceae, Muribaculaceae, {Unknown Family} RF39, and FamilyXI at the family level, and *Unknown Family Clostridia UCG-014*, *Anaerostipes*, *Erysipelotrichaceae UCG-003*, *Lachnospiraceae UCG-001*, *Unknown Genus Muribaculaceae*, *Prevotella_9*, *Agathobacter*, *Enterococcus*, *Hungatella*, *Intestinimonas*, *Enterobacter*, *Uncultured11*, *Butyricicoccus*, *Unknown Family RF39*, *Citrobacter*, *Lachnospiraceae NK4A136 group*, *Uncultured03*, *Klebsiella*, *Eubacterium xylanophilum group*, and *Romboutsia* at the genus level (Fig. 9). These discriminating taxa were used as classifiers in the subsequent prediction analysis. As it is the nature of microbiota to be distributed rather randomly, not all classifier genera were present in all subjects, nevertheless each taxon was present in a majority of samples. Supplementary Table S8 (Additional file 10) lists the relative abundances of the classifier families and genera. In addition, Supplementary Table S8 (Additional file 10) displays the differences in read counts of Unknown Family Clostridia UCG-014 between patients and controls as a prominent proxy for all microbial taxa included in the classifier.

#### Prediction performance by the taxonomic ranks of phylum, family, and genus

**Fig. 10:**
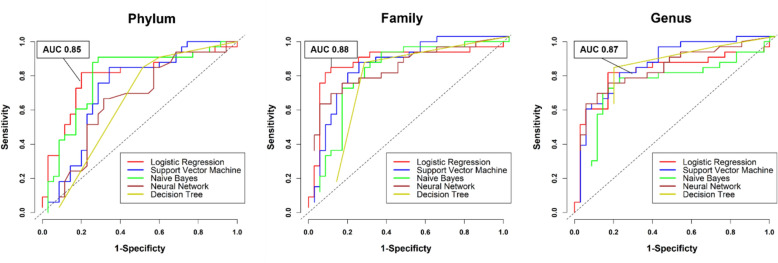
ROC curve analysis for PDAC prediction using five Machine Learning methods across the taxonomic ranks phylum, family, and genus. At the phylum level, the logistic regression model (95% CI 0.74, 0.95) achieved the best AUC of 0.85. At the family level, the logistic regression model (95% CI 0.78, 0.97) reached the highest AUC of 0.88. At the genus level, the SVM model performed best, with an AUC of 0.87 (95% CI 0.78, 0.95). AUC, area under the receiver operating characteristic (ROC) curve

In this step, we evaluated the performance of machine learning (ML) models for predicting PDAC across different taxonomic ranks, as shown in Fig. 10 and Table 5. At the phylum level, total variables, referring to all differentially abundant phyla (including Elusimicrobiota, Fusobacteriota, Verrucomicrobiota, Synergistota, and Proteobacteria), yielded an AUC of 0.85 (95% CI 0.74–0.95), with a sensitivity of 0.91 and a specificity of 0.86, which demonstrates strong predictive performance. Models with selected variables showed slightly reduced AUCs, with LR and NB both achieving AUCs of 0.79 (95% CI 0.68–0.91), and the other models achieving lower values. At the family level, LR with total variables resulted in a low AUC of 0.50 (95% CI 0.38–0.63), but with selected variables, LR achieved the highest AUC of 0.88 (95% CI 0.78–0.97), with a sensitivity of 0.85 and a specificity of 0.89. Finally, at the genus taxonomic rank, models with all variables performed poorly, with LR achieving an AUC of 0.42 (95% CI 0.30–0.54), while models employing selected variables showed significantly improved performance with SVM achieving the highest AUC of 0.87 (95% CI 0.78–0.95), with a sensitivity of 0.79 and a specificity of 0.80. For detailed results, see Table S7, Additional File 9.

**Table 5:** ROC curve performance metrics for PDAC prediction by taxonomic ranks and machine learning methods

Taxonomic ranks	Models and variables^a^	AUC^b^ (95% CI)	Sensitivity (95% CI)	Specificity (95% CI)	Positive predictive value (95% CI)	Negative predictive value (95% CI)	Accuracy (95% CI)
Phylum	LR–all variables	**0.85 (0.74, 0.95)**	0.91 (0.76, 0.97)	0.86 (0.71, 0.94)	0.86 (0.71, 0.94)	0.91 (0.76, 0.97)	0.87 (0.76, 0.94)
	LR–selected variables	0.79 (0.68, 0.91)	0.82 (0.66, 0.91)	0.80 (0.64, 0.90)	0.79 (0.63, 0.90)	0.82 (0.66, 0.92)	0.79 (0.68, 0.88)
	SVM–selected variables	0.72 (0.60, 0.85)	0.85 (0.69, 0.93)	0.66 (0.49, 0.79)	0.70 (0.55, 0.82)	0.82 (0.64, 0.92)	0.74 (0.61, 0.83)
	NB–selected variables	0.79 (0.68, 0.91)	0.91 (0.76, 0.97)	0.71 (0.55, 0.84)	0.75 (0.60, 0.86)	0.89 (0.73, 0.96)	0.81 (0.70, 0.89)
	NN – selected variables	0.66 (0.52, 0.79)	0.67 (0.50, 0.80)	0.69 (0.52, 0.81)	0.67 (0.50, 0.80)	0.69 (0.52, 0.81)	0.68 (0.55, 0.78)
	DT – selected variables	0.65 (0.53, 0.76)	0.85 (0.69, 0.93)	0.49 (0.33, 0.64)	0.61 (0.46, 0.74)	0.77 (0.57, 0.90)	0.66 (0.54, 0.77)
Family	LR – all variables	0.50 (0.38, 0.63)	0.64 (0.47, 0.78)	0.40 (0.26, 0.56)	0.50 (0.36, 0.64)	0.54 (0.35, 0.71)	0.51 (0.39, 0.64)
	LR – selected variables	**0.88 (0.78, 0.97)**	0.85 (0.69, 0.93)	0.89 (0.74, 0.95)	0.88 (0.72, 0.95)	0.86 (0.71, 0.94)	0.87 (0.76, 0.94)
	SVM – selected variables	0.86 (0.77, 0.95)	0.85 (0.69, 0.93)	0.77 (0.61, 0.88)	0.78 (0.62, 0.88)	0.84 (0.68, 0.93)	0.78 (0.66, 0.87)
	NB – selected variables	0.81 (0.70, 0.92)	0.91 (0.76, 0.97)	0.66 (0.49, 0.79)	0.71 (0.56, 0.83)	0.88 (0.71, 0.96)	0.78 (0.66, 0.87)
	NN – selected variables	0.84 (0.74, 0.94)	0.73 (0.56, 0.85)	0.86 (0.71, 0.94)	0.83 (0.65, 0.92)	0.77 (0.62, 0.87)	0.79 (0.68, 0.88)
	DT – selected variables	0.77 (0.65, 0.88)	0.85 (0.69, 0.93)	0.74 (0.58, 0.86)	0.76 (0.60, 0.87)	0.84 (0.67, 0.93)	0.79 (0.68, 0.88)
Genus	LR – all variables	0.42 (0.30, 0.54)	1.00 (0.90, 1.00)	0.00 (0.00, 0.10)	0.49 (0.37, 0.60)	NA (NA, NA)	0.49 (0.36, 0.61)
	LR – selected variables	0.82 (0.71, 0.93)	0.82 (0.66, 0.91)	0.83 (0.67, 0.92)	0.82 (0.66, 0.91)	0.83 (0.67, 0.92)	0.81 (0.70, 0.89)
	SVM – selected variables	**0.87 (0.78, 0.95)**	0.79 (0.62, 0.89)	0.80 (0.64, 0.90)	0.79 (0.62, 0.89)	0.80 (0.64, 0.90)	0.79 (0.68, 0.88)
	NB – selected variables	0.75 (0.62, 0.88)	0.76 (0.59, 0.87)	0.80 (0.64, 0.90)	0.78 (0.61, 0.89)	0.78 (0.62, 0.88)	0.78 (0.66, 0.87)
	NN – selected variables	0.83 (0.73, 0.93)	0.73 (0.56, 0.85)	0.86 (0.71, 0.94)	0.83 (0.65, 0.92)	0.77 (0.62, 0.87)	0.79 (0.68, 0.88)
	DT – selected variables	0.85 (0.75, 0.94)	0.82 (0.66, 0.91)	0.83 (0.67, 0.92)	0.82 (0.66, 0.91)	0.83 (0.67, 0.92)	0.82 (0.71, 0.91)

AUC, area under the curve; CI, confidence interval; DT, decision tree; LR, logistic regression; NB, naïve Bayes; NN, neural network; SVM, support vector machine

^a^“All variables” refers to all differential taxa at the respective taxonomic level, “selected variables” refers to the microbial classifier at the respective taxonomic level, determined by logistic regression, with an AUC threshold of  > 0.7 (see also Fig. 9). At the phylum level, Firmicutes, Bacteroidota, and Cyanobacteria were included in the classifier. At the family level, {Unknown Family} Clostridia UCG-014, Enterococcaceae, Prevotellaceae, Butyricicoccaceae, Enterobacteriaceae, Erysipelatoclostridiaceae, Muribaculaceae, {Unknown Family} RF39, and FamilyXI were selected. At the genus level, the selected variables included *Unknown Family Clostridia UCG-014, Anaerostipes, Erysipelotrichaceae UCG-003, Lachnospiraceae UCG-001, Unknown Genus Muribaculaceae, Prevotella_9, Agathobacter, Enterococcus, Hungatella, Intestinimonas, Enterobacter, Uncultured11, Butyricicoccus, Unknown Family RF39, Citrobacter, Lachnospiraceae NK4A136 group, Uncultured03, Klebsiella, Eubacterium xylanophilum group*, and *Romboutsia*

^b^The highest AUC scores are bolded

## Discussion

In this study, we had the unique opportunity to profile the PC stool microbiota in two divergent populations —Finland and Iran— with different geographical and sociocultural backgrounds, however, using consistent analytical methods. We analyzed the cancer gut microbiota within each population and established microbial classifiers for predicting PC, that were generated in the Iranian cohort and validated in the Finnish cohort.

Our results indicate that PC is associated with a distinct gut microbial profile. Common features across both populations included significantly lower alpha diversity indices in PC patients, significant differences in beta diversity between the cancer and control groups, and significant shifts in the abundance of certain bacterial taxa. While the microbial signatures of Finnish and Iranian PC patients differed in some respects, they overlapped sufficiently, so that the classifiers created in one cohort was successfully used for PC prediction in the other cohort, demonstrating a high predictive performance. Fecal microbial classifiers have been proposed as noninvasive diagnostic and prognostic markers for various cancers [41–47], particularly CRC [48, 49], where they have been extensively investigated and have reached the clinical trial phase [50–53]. Comparable studies for PC are rare, but promising results have recently been obtained in a Spanish and two Japanese cohorts, based on 27, 30, and 24 differential species, with high AUCs of 0.84 [54], 0.72 [55], and 0.91 [56], respectively. Our classifiers, consisting of 9 families and 20 genera, aligned with these, achieving excellent AUCs of 0.88 (95% CI 0.78–0.97) and 0.87 (95% CI 0.78–0.95), respectively. Despite its strong performance, this method is insufficient for early screening applications and could be improved by combining it with the CA 19–9 marker, as demonstrated previously [54, 55]. Further refinement to the species level through shotgun sequencing or quantitative real-time PCR could increase the predictive accuracy.

The observation of significantly lower phylogenetic and alpha diversity indices in PC patients compared to HCs aligns with the results of earlier PC studies [55, 57, 58]. Typically, higher alpha diversity is associated with a healthy and stable microbiome due to increased microbial functional redundancy [59, 60], and lower alpha diversity has been linked to various medical conditions, including cancer [61–65]. However, several studies reported a stronger influence of geographical or ethnic factors than disease status on alpha diversity [66–70], which we could not confirm. We did not find any differences in alpha diversity between the populations, whether we compared patients, controls, or the populations as a whole.

Consistent with earlier studies [54–58, 71], beta- or interindividual species diversity differed significantly between PC and HCs within the populations. Beta diversity also differed between the populations, which is not surprising and is likely a consequence of different host genomes, lifestyles, and dietary habits. Interestingly, the differences in beta diversity between PC and HCs were more pronounced in the Iranian cohort than in the Finnish cohort (pseudo-F = 5.13 and 2.37, respectively). This could be caused by the diverging age distributions between Iranian PC and HCs compared with the Finnish cohort, since the microbial community composition is known to change with age [72]. However, inter-cohort comparisons of patients vs. patients and controls vs. controls revealed similar beta diversity differences, suggesting the effects of factors other than age. In our merged dataset including both populations, the differences between all PC cases and all HCs had similarly high pseudo-F values as those between all Finns and all Iranians. This finding indicates equally strong impacts of PC and population origin on the gut microbial community composition and contrasts with the literature. In a study by Half et al. that compared fecal microbiota profiles of Israeli and Chinese PC cohorts, ethnic origin had a stronger effect on microbial community composition than cancer did [71]. Notably, unlike our study, the analytical methodologies differed between the cohorts [57, 71], which might have influenced their outcomes.

Differential abundance analysis revealed characteristic compositional features of the PC gut microbiota shared by both populations: overrepresentation of potentially pathogenic bacteria, such as Enterococcaceae, Fusobacteriaceae, Enterobacteriaceae, and Veillonellaceae, and underrepresentation of taxa associated with healthy gut flora, such as SCFA-producing Clostridia, which confirms previous findings [54–57, 71, 73]. Several overrepresented taxa are gram-negative and thus lipopolysaccharide (LPS)-producing. As components of the outer bacterial membrane, LPS interact with the immune system, mediating inflammation and participating in various pathogenic processes [74, 75]. In PC cells, LPS have been shown to activate the PI3K/Akt/mTOR pathway, a known oncogenic driver [76]. This provides a plausible mechanism by which the overrepresented gram-negative bacteria in PC could activate an oncogenic pathway and contribute to tumorigenesis. The most enriched phylum in PC in both populations, the gram-negative Fusobacteriota, contains the oral opportunistic pathogen *Fusobacterium nucleatum*, which is considered a crucial factor in CRC tumorigenesis and progression [77]. Enriched levels of *F. nucleatum* have been detected in PC saliva [78], gut microbiota [54, 56, 73], and tumor tissue [79] and might therefore play important roles in PC tumorigenesis too. Another prominent phylum associated with PC is the gram-negative Proteobacteria [56, 80], which comprises the known pathogens *E. coli*, *Shigella*, *Klebsiella, Enterobacter, Salmonella*, and *Yersinia*, among others. Notorious for their involvement in inflammation and disease [81], Proteobacteria have been associated with metabolic disorders and IBD [81, 82], as well as different types of cancer, including PC [83–86]. The enriched Proteobacteria families in Finnish patients consisted of Yersiniaceae and Hafniaceae, whereas those in Iranian patients included Xanthomonadaceae and Pseudomonadaceae, the latter of which have also been detected in PC tissue [80, 87]. Confirming earlier findings in the PC gut microbiota [80], the gram-negative facultative pathogen Synergistota was enriched in PC in both populations. Gram-negative Campylobacterota, including the pathogens *Helicobacter* and *Campylobacter*, were enriched in Finnish PC only and have both been associated with cancer [85, 88, 89]. Streptococcaceae, which include the oral pathogen *Streptococcus* and which have been linked to malignancies such as CRC and gastric cancer [90–93]*,* were enriched in Iranian PC, which aligns with findings in Japanese PC cohorts [55, 58, 94, 95]. Furthermore, beneficial but potentially pathogenic Lactobacillaceae [96–98 and Akkermansiaceae [99, 100] were enriched in Iranian PC, which is consistent with findings in Spanish [54] and Japanese [55, 94, 95], and in Spanish [54], Israeli [71], and Greek [73] PC cohorts, respectively. Interestingly, *Lactobacillus* and *Akkermansia* have also been detected in PC tumor tissue [54, 73], suggesting a possible involvement in PC tumorigenesis and progression.

The taxa depleted in PC in both cohorts included Bacilli RF39, which are beneficial as putative producers of acetate and hydrogen [101], and members of the Clostridia class (see Fig. 5B and Table S4, Additional file 5). Several studies have reported an underrepresentation of butyrate-producing Clostridia in cancer [30, 102–105], including PC [55, 58, 71] (see also Supplementary Table S9, Additional file 11, for an overview of recent PC-related microbiota studies). Selected members of this class can modulate inflammation [106] and support anticancer immune responses [30]. The Clostridia *Eubacterium* and *Anaerostipes*, depleted in CRC [30] and PC [55–57, 71, 94, 95], have been utilized as effective antitumor treatments in CRC mouse models [30]. These genera were also depleted in Iranian patients, suggesting that they might have comparable antitumor capacities in PC. Conversely, *Peptostreptococcus stomatis* has been found overrepresented in CRC [107] and has also been associated with a greater tumor burden in CRC [30]. We observed higher levels of Peptostreptococcales-Tissierellales family members in PC, namely, *Finegoldia* in Finnish patients and *Mogibacterium* and *Clostridioides* in Iranian patients. These genera might carry out analogous cancer-promoting functions in PC as *P. stomatis* does in CRC. Since the abovementioned Clostridiales strains may play crucial roles in PC, future efforts in developing gut microbiota supplementation therapies for PC should focus on these microbes, aiming to restore a healthy gut microbiome and potentially impede cancer progression.

A comparison of the microbial profiles between the populations revealed both similarities and clear differences. With respect to large-scale community composition, distinct differences were noted in the dominant phyla Bacteroidota and Firmicutes. In both cohorts, the relative abundance of Bacteroidota was greater, whereas that of Firmicutes was lower, in PC compared to HCs. A shift in the Firmicutes to Bacteroidota (F/B) ratio has been associated with dysbiosis [108], and decreased F/B ratios have been observed in several types of cancer [109–112], including PC [56, 57, 71]. In this study, the F/B ratio was 30.0% lower in the Finnish cohort (F/B_FPDAC_ = 1.03; F/B_FHC_ = 1.47) and 25.3% lower in the Iranian cohort (F/B_IPDAC_ = 1.92; F/B_IHC_ = 2.57) in patients than in their respective controls (Table S2, Additional file 3). Interestingly, in the differential abundance analysis, Bacteroidota was significantly enriched, and Firmicutes was significantly depleted in Finnish PC compared with Iranian PC, likely due to varying lifestyles, particularly dietary habits. Accordingly, *Bacteroides,* the most dominant bacterial genus in the gut, had a significantly greater abundance in Finnish PC than in Iranian PC. Higher *Bacteroides* abundance has been linked to a Western-type lifestyle characterized by a diet rich in protein and animal fats [113], which may explain the higher levels of *Bacteroides* in Finnish patients. Finns typically consume a diet high in animal fats and processed meats, with pork, chicken, and beef as primary protein sources and potatoes and wheat as primary carbohydrate sources [114]. In contrast, Iranians predominantly consume rice, often twice daily, and exclude pork in favour of mutton, owing to cultural and religious reasons [115, 116]. In addition to the differences in the two dominant phyla, the third and fourth most abundant phyla in the gut, Proteobacteria and Actinobacteriota, also exhibited significant differences between the populations, with notably higher abundances in Iranian patients than in their Finnish counterparts. These differences might likewise be attributable to dietary variations. Actinobacteriota have been positively associated with the intake of resistant starch [117], which is found in foods such as legumes and cooked and cooled rice [118], as well as the consumption of fermented dairy products [119]. Proteobacteria on the other hand have been reported to increase with the consumption of red meat [120], the intake of a calorie-dense, high-fat, low-fibre diet [121], and, consequently, obesity [122]. However, increased levels of these phyla may also reflect population-specific PC dysbiosis. Several Actinobacteriota genera enriched in Iranian versus Finnish PC belong to the oral microbiome and are involved in oral infections, e.g., *Actinomyces* [123], *Scardovia* [124], and *Rothia* [125], or are otherwise pathogenic, e.g., *Eggerthella* [126]. A noteworthy member of the Actinobacteriota, *Bifidobacterium*, which was enriched in Iranian versus Finnish PC, is known for its beneficial effects on the gut microbiome, is used as a probiotic [127], and promotes antitumor immunity [128]; however, in rare cases, this genus can act as a pathogen that causes bacteremia, particularly in immunocompromised individuals [129]. *Bifidobacterium* has previously been detected in PC tumor tissue [54], in the gut microbiota [73], in the duodenal fluid [130], and in the vermiform appendix [131] of PC patients, indicating its possible involvement in PC tumorigenesis.

Other major factors influencing the gut microbiome include alcohol and tobacco consumption, which lead to shifts in microbial community composition towards dysbiosis and decreased microbial diversity [132–134]. For cultural and religious reasons, alcohol consumption varies significantly between the countries, with a markedly higher per capita alcohol consumption of 9.2 L in Finland compared to 0.7 L in Iran in 2019 [135]. In our cohorts, over 60% of patients and over 50% of HCs in the Finnish, but only approximately 4% of patients and 9% of controls in the Iranian cohort reported alcohol consumption. Similarly, smoking habits differ between the two countries, with reported tobacco use by 17% of Finns and 9% of Iranians in 2020 [136], and were also distinct between our study populations, albeit less dramatically. These differences in alcohol and tobacco consumption likely contributed to the divergent microbial profiles observed between the populations.

The microbial function prediction analysis further highlighted the overall diversity between the populations while also demonstrating similar trends. Notably, two of the most enriched predicted functions, subfamily C and inositol transport system permease protein, are linked to ATP-binding cassette (ABC) transporters. ABC transporters mediate multidrug resistance [137, 138], play critical roles in the virulence of several microbial pathogens [139], and have been associated with cancer [140, 141]. Additionally, two significantly enriched predicted functions in Iranian PC are linked to the pathogen *Staphylococcus aureus*: clumping factor B, a virulence factor in *S. aureus* infection [142, 143], and accessory secretory protein Asp3, which is involved in the export of surface glycoproteins in *S. aureus* and other gram-positive bacteria [144]. *S. aureus* infection has been associated with an increased risk of primary cancer, including PC, possibly caused by tumor-associated immune suppression [145]. In contrast, three of the top decreased differential functions in Finnish PC are related to environmental stress signalling in *Bacillus subtilis*: the serine/threonine-protein kinase RsbT, the RsbT antagonist protein RsbS, and the RsbT coantagonist protein RsbR [146–148]. *B. subtilis* is a beneficial microbe known for modulating host metabolite pathways [149] and boosting immunity [150]. Since these microbial functions are predictions only based on 16S rRNA gene amplicon sequencing, further functional studies are needed to confirm these results.

Pathway analysis revealed PC-linked enrichment of pathways related to the biosynthesis of peptidoglycan and lysine; galactose metabolism; carbon fixation in prokaryotes; and the degradation of benzoate, toluene, and furfural. Peptidoglycan, a critical component of the bacterial cell wall, and lysine, an essential amino acid and protein precursor, are fundamental to bacterial growth. The enrichment of these pathways might be linked to the increase in peptidoglycan-producers, that is, gram-positive bacteria. Gram-positive microbes, such as enterococci, staphylococci, and streptococci have been shown to be the main responsible for invasive bacterial disease in cancer patients [151]. In our case, the cancer patients had higher abundances of enterococci. Galactose metabolism involves the fermentation of galactose into lactic acid, a process carried out by various gut microbes, especially lactic acid bacteria (LAB), such as *Lactobacillus* [152]. The enrichment of this pathway is likely associated with the increased *Lactobacillaceae* in Iranian PC compared with HCs. Carbon fixation is a key process in autotrophic organisms such as plants and cyanobacteria [153], but it has also been detected in heterotrophic *E. coli* [154]. Therefore, the enrichment of this pathway might be associated with an increase in opportunistic pathogenic anaerobes such as *E. coli*. The enrichment of pathways related to the biodegradation of toluene, benzoate, and furfural may be associated with increased xenobiotics intake through smoking [155] and the consumption of processed foods, as sodium benzoate is widely used as a food preservative [156, 157]. Overall, microbial function prediction and pathway analysis underscore distinct microbial features between the populations, likely driven by different lifestyles and dietary habits.

This study had some limitations that should be taken into consideration. Since pancreatic cancer is a relatively rare disease, and only a fraction of patients undergo surgery, the number of available samples was limited, which caused low statistical power in the analyses. Due to organizational circumstances, also healthy controls were limited and did not match the patients 1:1 across all clinical and lifestyle parameters. For example, age and smoking status differed significantly between Iranian patients and controls. To mitigate these imbalances, we applied corrections in the differential abundance analysis. Moreover, patients and HCs had comorbidities to varying extents, which were difficult to control for. Another limitation of this study was the fact that stool sampling differed between our cohorts. We attempted to minimize these differences by treating the Iranian samples similarly to the Finnish ones before DNA extraction, as described in the methods section. Concerning storage conditions, they varied between the cohorts. However, for practical reasons all samples were stored at − 20 °C for at least five months before DNA extraction, which is not ideal for stool samples but increases consistency. As a major strength of this study, DNA extraction and subsequent microbial analyses were performed simultaneously using identical methods in both populations, thereby reducing methodological impacts on population differences. To reinforce our findings, larger cohorts in both populations are needed, and validation of the microbial classifiers in large public datasets of healthy individuals and patients with PC and other medical conditions from various populations and geographic backgrounds is essential in future studies. Despite these shortcomings, our study adds very valuable insights to the present knowledge on pancreatic cancer microbiota, especially in terms of population differences.

## Conclusion

Our study identified a distinct gut microbial profile for patients with pancreatic cancer (PC) that was independent of patients’ geographic or cultural backgrounds. We observed consistent trends in PC-related microbial diversity and community composition in our two populations—Finnish and Iranian—with profoundly different environments and lifestyles. These findings suggest that the gut microbiota plays a crucial role in the development of PC, likely through the increased prevalence of pathogenic microbes with proinflammatory and tumor-promoting functions and the depletion of protective microbes, such as butyrate- and other short-chain fatty acid- (SCFA-) producers. Moreover, we show that this unique microbial profile has potential as a classifier for PC and could be used for noninvasive early PC screening. However, further refinement and validation in larger, more diverse cohorts are necessary to enhance its predictive accuracy. Finally, the results of our differential abundance analysis, particularly the depletion of Clostridia, offer promising future avenues for developing novel treatment strategies for PC. These could involve the integration of next-generation probiotics alongside conventional chemotherapeutic drugs, potentially offering a more targeted and effective approach to managing this challenging disease. Future research should explore these therapeutic possibilities, aiming to translate microbial insights into clinical interventions.

## Supplementary Information

Additional file 1: Table S1. Sequencing statistics and power calculations.

Additional file 2: Figure S1. Alpha diversities of covariate groups.

Additional file 3: Table S2. Relative abundances of gut microbes. Relative abundances of gut microbes per clinical group on several taxonomic levels.

Additional file 4: Table S3. Beta diversity. PERMANOVA testing of covariates.

Additional file 5: Table S4. Differential abundance analysis of gut microbes.

Additional file 6: Table S5. Predicted microbial functions and pathway analysis.

Additional file 7: Figure S2. Mean Decrease Gini. Variable Importance Analysis for PDAC prediction using Mean Decrease Gini across the taxonomic ranks phylum, family, and genus.

Additional file 8: Table S6. Variable selection for PDAC prediction. Variable selection for PDAC prediction based on phylum, family, and genus variables using logistic regression.

Additional file 9: Table S7. ROC curve analysis for PDAC prediction performance metrics. ROC curve analysis for PDAC prediction performance metrics by taxonomic phylum-, family- and genus-based and machine learning methods using different feature selections methods

Additional file 10: Table S8. Classifier relative abundance and Clostridia UCG-014. Relative abundances of the classifier families (n = 9) and genera (n = 20). In addition, this file contains per-sample read counts and relative abundances of one representative classifier taxon, Clostridia UCG-014. A boxplot of the Clostridia UCG-014 read counts visualizes the differences between the groups.

Additional file 11: Table S9. Overview of PDAC-related microbiota studies. This table compiles 18 recent studies on PC microbiota with details on bacteria with higher or lower abundance in fecal and oral samples of PC patients compared to healthy controls.

## Data Availability

Data access is restricted due to personal information protection (General Data Protection Regulation (GDPR) 2016/679 and Directive 95/46/EC). However, the datasets used in the current study are available from the corresponding author upon reasonable request.
