# Correction: Fecal profiling reveals a common microbial signature for pancreatic cancer in Finnish and Iranian cohorts

**DOI:** 10.1186/s13099-025-00710-7

**Published:** 2025-05-23

**Authors:** Heidelinde Sammallahti, Sama Rezasoltani, Satu Pekkala, Arto Kokkola, Hamid Asadzadeh Agdaei, Mehdi Azizmohammad Looha, Reza Ghanbari, Farhad Zamani, Amir Sadeghi, Virinder Kaur Sarhadi, Marja Tiirola, Pauli Puolakkainen, Sakari Knuutila

**Affiliations:** 1 Department of Pathology, Faculty of Medicine, University of Helsinki, 00014 Helsinki, Finland; 2 Department of Surgery, Abdominal Center, University of Helsinki, Helsinki University Hospital, 00290 Helsinki, Finland; 3 Division of Oral Microbiology and Immunology, Department of Operative Dentistry, Periodontology and Preventive Dentistry, Rheinisch-Westfälische Technische Hochschule (RWTH) University Hospital, 52074 Aachen, Germany; 4 Faculty of Sport and Health Sciences, University of Jyväskylä, 40014 Jyväskylä, Finland; 5 Department of Surgery, University of Helsinki and Helsinki University Hospital, 00290 Helsinki, Finland; 6 Basic and Molecular Epidemiology of Gastrointestinal Disorders Research Center, Research Institute for Gastroenterology and Liver Diseases, Shahid Beheshti University of Medical Sciences, P.O. Box 1985717411, Tehran, Iran; 7 Gene Therapy Research Center, Digestive Diseases Research Institute, Shariati Hospital, Tehran University of Medical Sciences, Tehran, Iran; 8 Gastrointestinal and Liver Diseases Research Center, Iran University of Medical Sciences, Tehran, Iran; 9 Gastroenterology and Liver Diseases Research Center, Shahid Beheshti University of Medical Sciences, Tehran, Iran; 10 Department of Oral and Maxillofacial Diseases, Helsinki University Hospital and University of Helsinki, 00290 Helsinki, Finland; 11 Department of Environmental and Biological Sciences, Nanoscience Center, University of Jyväskylä, 40014 Jyväskylä, Finland; 12 BiopSense Oy, Eeronkatu 10, 40720 Jyväskylä, Finland

**Correction: Gut Pathogens (2025) 17:24** 10.1186/s13099-025-00698-0

In this article [1], the author’s name Mehdi Azizmohammad Looha was incorrectly written as Mehdi Azizmohhammad Looha.

The original article has been corrected
