# Supplementary material for: Fecal profiling reveals a common microbial signature for pancreatic cancer in Finnish and Iranian cohorts
Source: Gut Pathog. 2025 Apr 16;17:24. doi: 10.1186/s13099-025-00698-0 (PMC12001732; doi:10.1186/s13099-025-00698-0)
Supplement: Supplementary file 2 — Additional file 2: Figure S1. Alpha diversities of covariate groups. [file 13099_2025_698_MOESM2_ESM.docx]

**Supplementary Figure S1**. **Alpha diversities of covariate groups.**

*Statistically significant differences (*p* < 0.05). FHC, Finnish HC; FPDAC, Finnish PDAC; HC, healthy control; IHC, Iranian HC; IPDAC, Iranian PDAC; PDAC, pancreatic ductal adenocarcinoma.

1. Both cohorts – All PC vs. All HC*


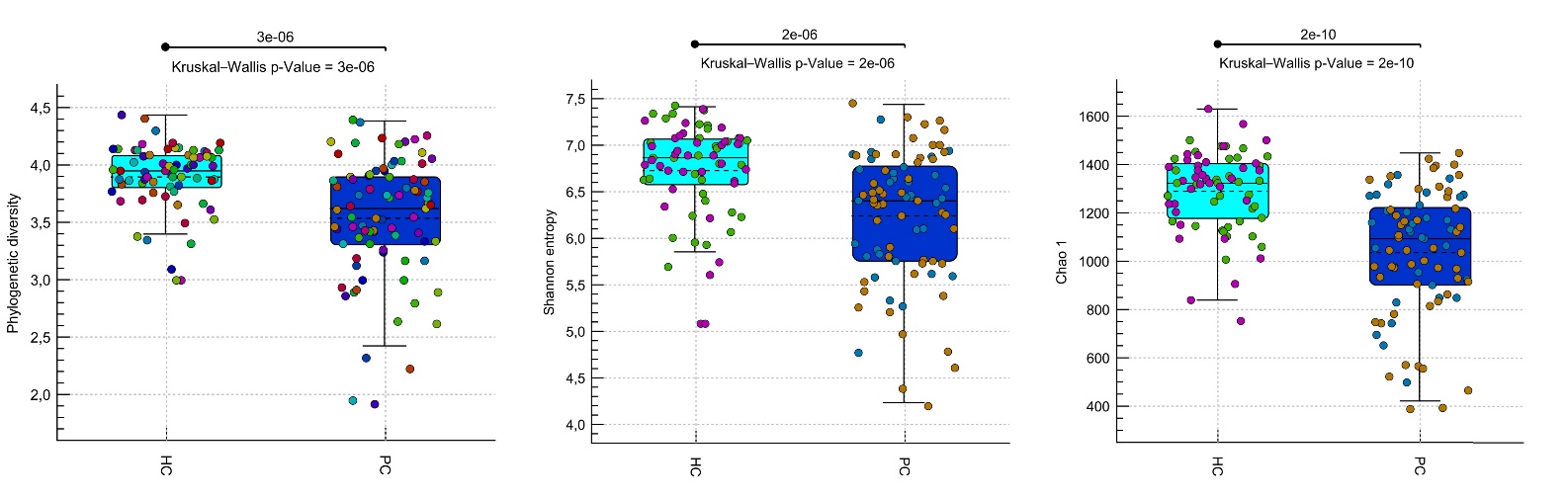


*

*

*

1. Both cohorts – Population (Finnish/Iranian)


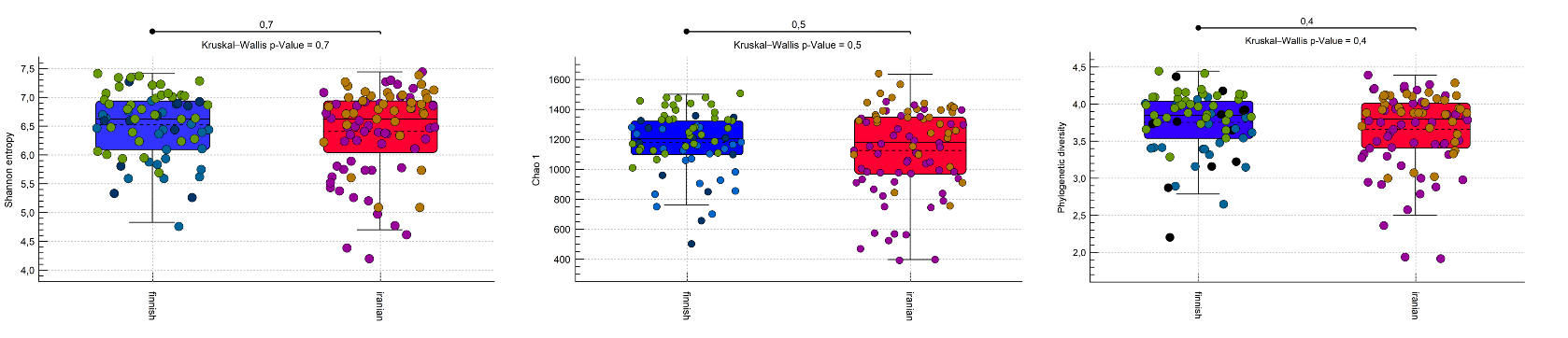


1. Finnish cohort – Age (2 = 40-59, 3 = 60-80 years)


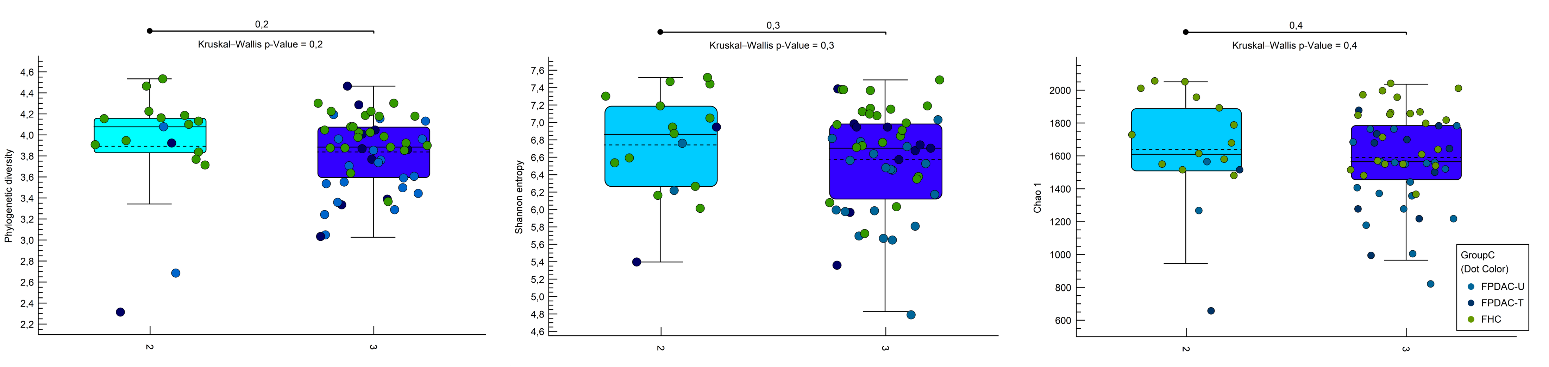


1. Finnish cohort – Alcohol use (1 = no, 2 = yes)


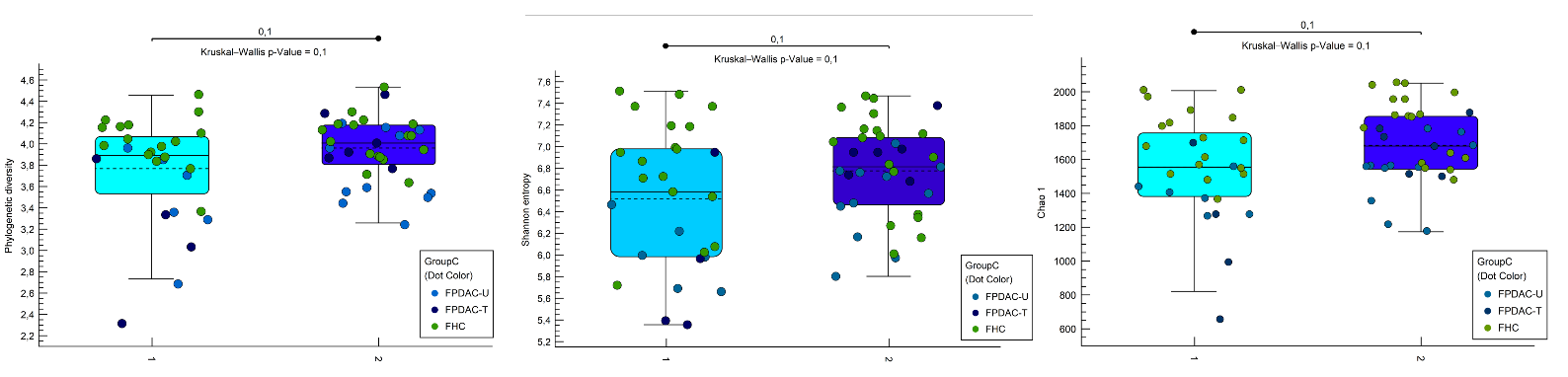


1. Finnish cohort – Biliary stent (1 = no, 2 = yes)


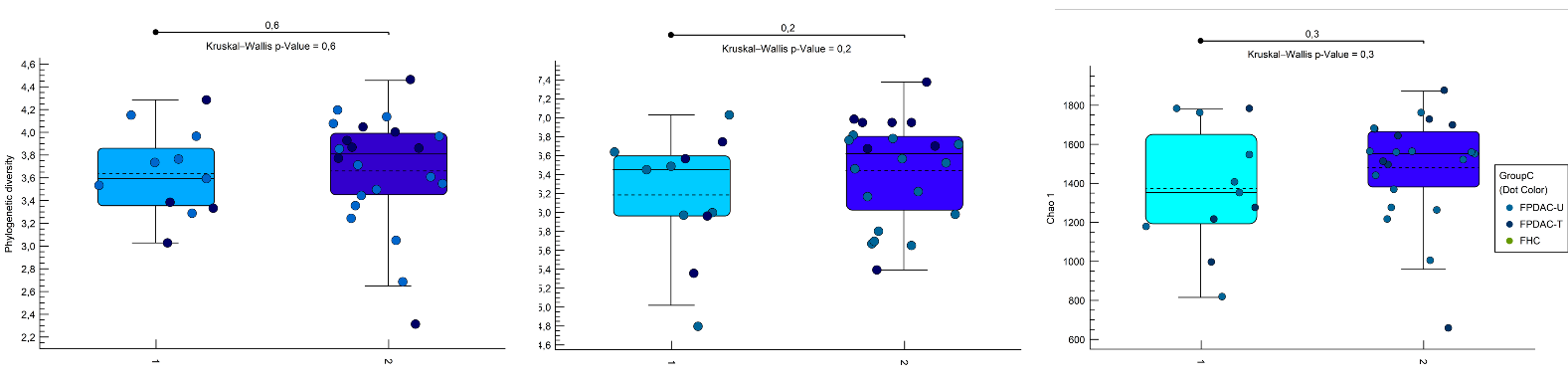


1. Finnish cohort – BMI categorical (1 < 20 (underweight), 2 = 20-26,99 (normal), 3 = 27-29,99 (overweight), 4 > 30 (obese))*


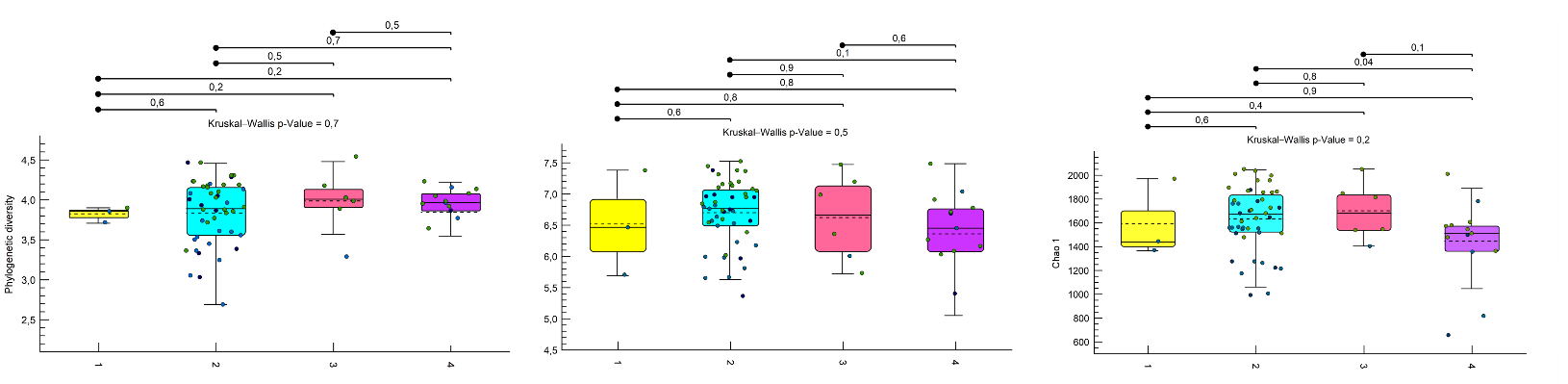


*

1. Finnish cohort – Neoadjuvant treatment (1 = no, 2 = yes)


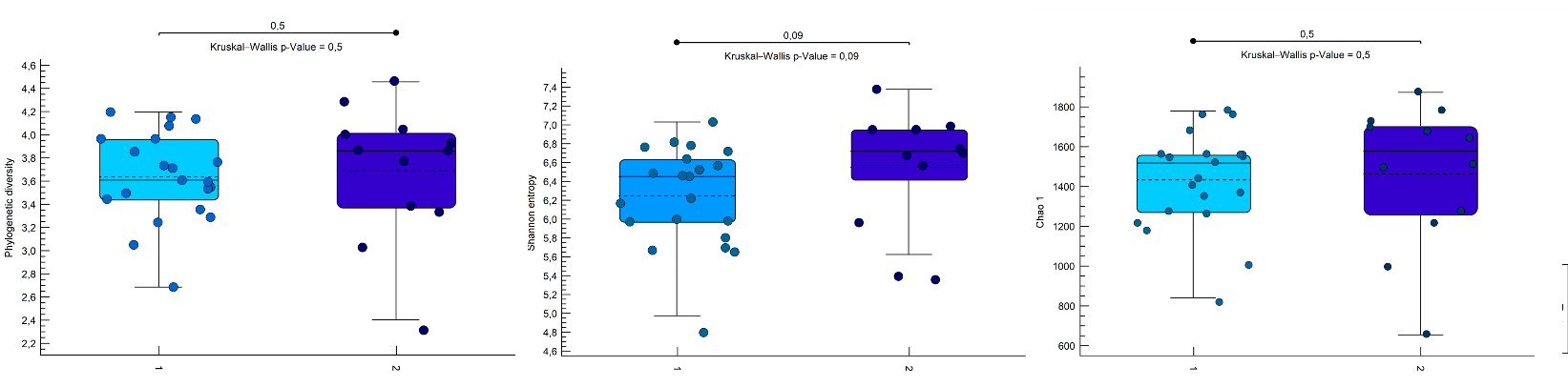


1. Finnish cohort – Sex (f = female, m = male)


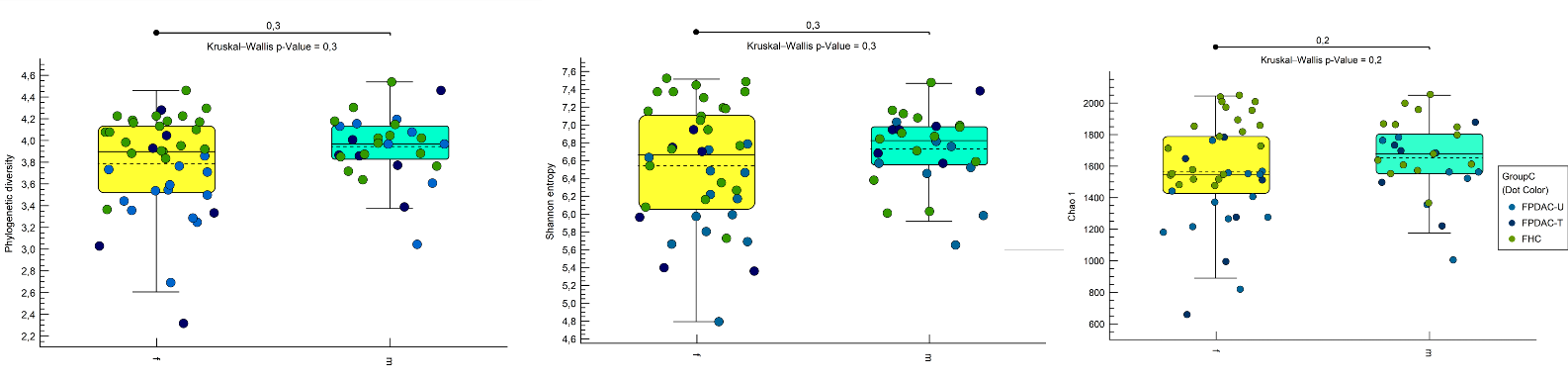


1. Finnish cohort – Smokers (1 = no, 2 = yes)


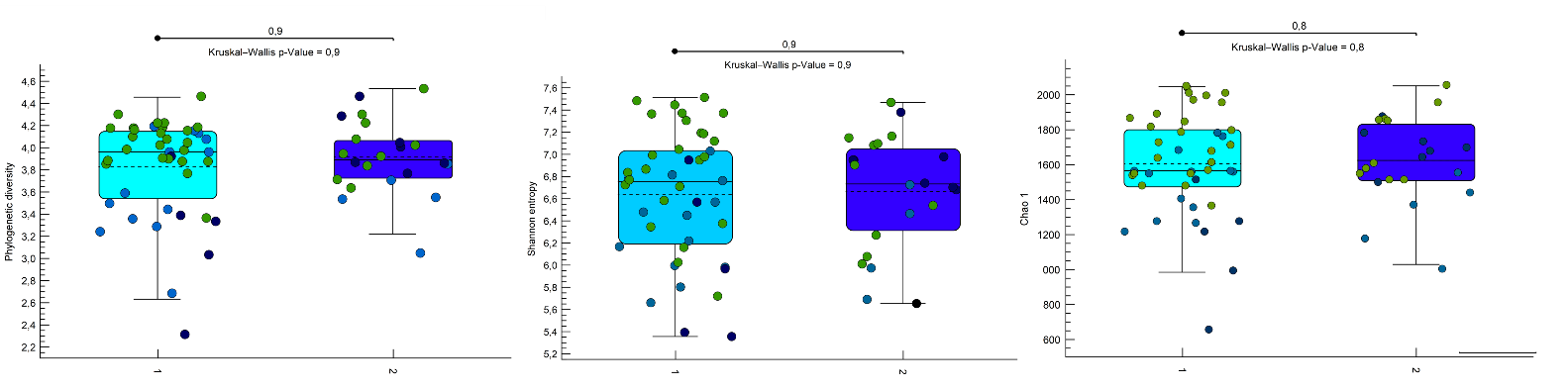


1. Iranian cohort – Age (1 < 40, 2 = 40-59, 3 = 60-80 years)*


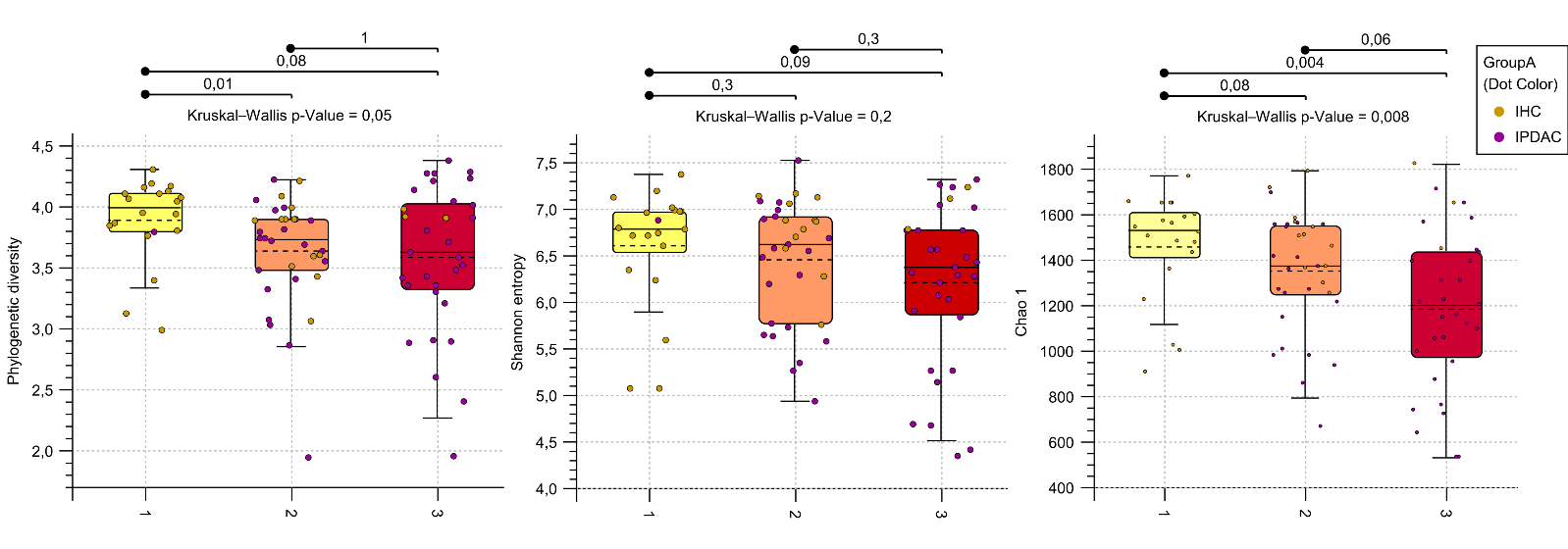


*

*

1. Iranian cohort – Alcohol use (1 = no, 2 = yes)


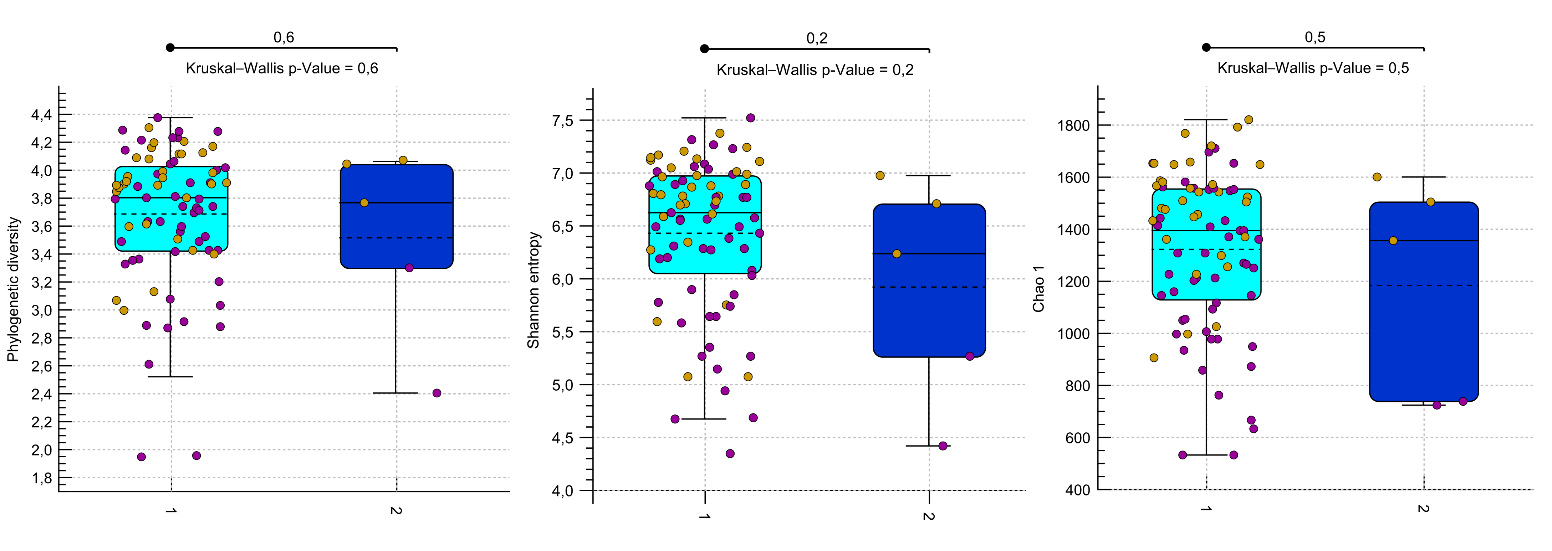


1. Iranian cohort – Sex (f = female, m = male)


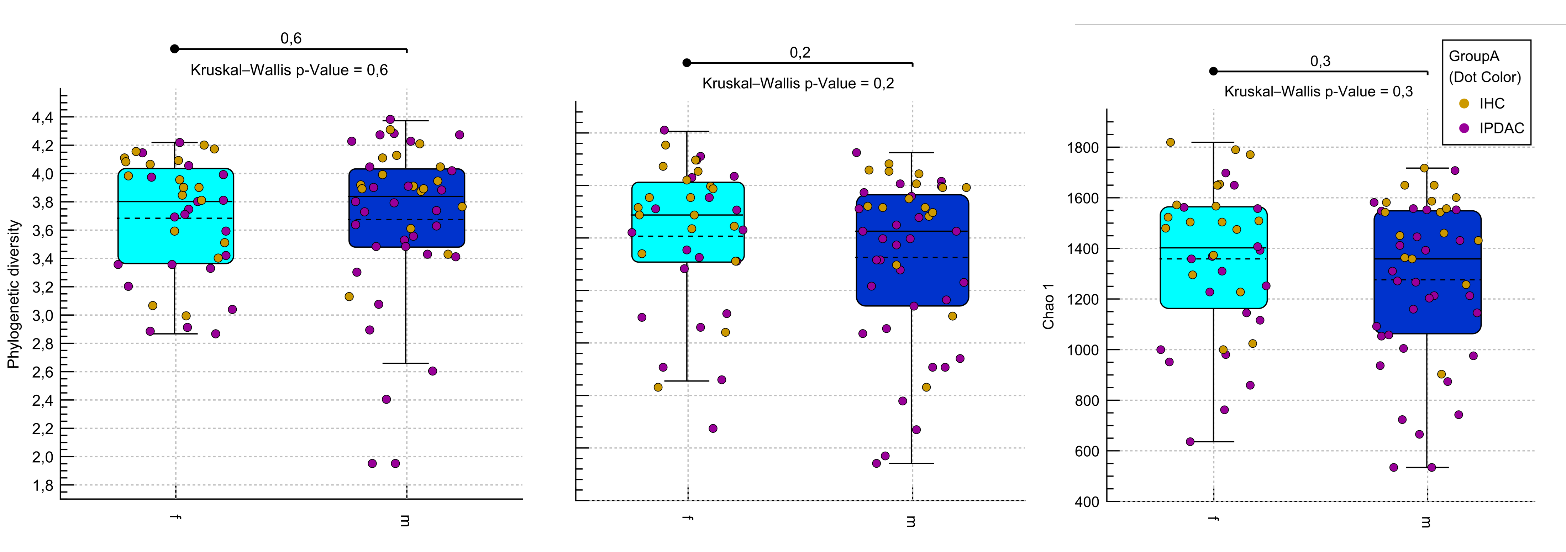


1. Iranian cohort – Smokers (1 = no, 2 = yes)*


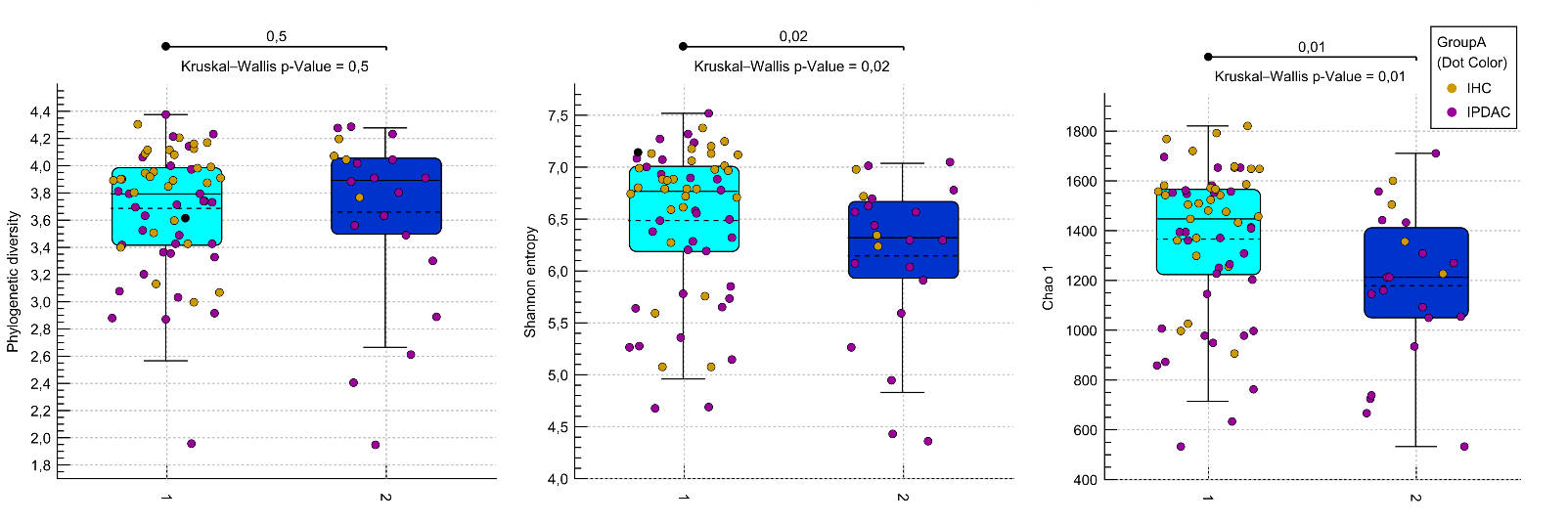


*

*
