# Supplementary material for: Fecal profiling reveals a common microbial signature for pancreatic cancer in Finnish and Iranian cohorts
Source: Gut Pathog. 2025 Apr 16;17:24. doi: 10.1186/s13099-025-00698-0 (PMC12001732; doi:10.1186/s13099-025-00698-0)
Supplement: Supplementary file 4 — Additional file 4: Table S3. Beta diversity. PERMANOVA testing of covariates. [file 13099_2025_698_MOESM4_ESM.docx]

**Supplementary Table S3**. **PERMANOVA testing of microbial community composition for covariates**

| **A. Finnish cohort** | | | | | |
| --- | --- | --- | --- | --- | --- |
| **Variable** | **Groups** | **Group comparisons**^1^ | **pseudo-F statistic**^2^ | ***p* value** | ***p* value (Bonferroni)**^3^ |
| Age group^4^ | 2, 3 | 2 vs. 3 | 0.79364 | 0.88387 | 0.88387 |
| Alcohol consumption | no, yes, NA | no vs. yes | 1.08469 | 0.28968 | 0.86904 |
| BMI categorical^5^ | 1–4 | all categories compared with each other | 0.81443–1.37053 | 0.03922–0.84723 | ns |
| Neoadjuvant treatment | HC, treated PDAC,  untreated PDAC | untreated vs. treated PDAC | 1.11264 | 0.24937 | 0.74811 |
|  |  | untreated PDAC vs. HC | 2.38525 | 0.00001 | **0.00003** |
|  |  | treated PDAC vs. HC | 1.57569 | 0.00610 | **0.01830** |
| Sex | female, male | female vs. male | 1.05029 | 0.35134 | 0.35134 |
| Stent | unstented, stented, NA | stented vs. unstented FPDAC | 1.12540 | 0.23227 | 0.69681 |
| Smoking | nonsmokers, smokers, NA | nonsmokers vs. smokers | 0.78761 | 0.89272 | 1.00000 |
| **B. Iranian cohort** | | | | | |
| **Variable** | **Groups** | **Group comparisons**^1^ | **pseudo-F statistic**^2^ | ***p* value** | ***p* value (Bonferroni)**^3^ |
| Age group^4^ | 1–3 | 1 vs. 2 | 1.45697 | 0.04685 | 0.14055 |
|  |  | 1 vs. 3 | 3.21561 | 0.00001 | **0.00003** |
|  |  | 2 vs. 3 | 1.77710 | 0.00900 | **0.02700** |
| Alcohol consumption | no, yes, NA | no vs. yes | 0.69134 | 0.95712 | 0.95712 |
| Sex | female, male | female vs. male | 1.66975 | 0.01583 | **0.01583** |
| Smoking | nonsmokers, smokers, NA | nonsmokers vs. smokers | 2.14587 | 0.00150 | **0.00450** |
| ^1^ Only relevant comparisons are listed, not the ones involving “NA” values.  ^2^ Pseudo-F is a measure of effect size. The larger it is, the greater the difference in the respective comparison.  ^3^ Low p-values (< 0.05, bolded) indicate significantly different average community compositions in the compared groups.  ^4^ Age groups: 1 ≤ 40 years, 2 = 40–59 years, 3 = 60–80 years.  ^5^ BMI categories: 1 ≤ 20 kg/m^2^(underweight), 2 = 20–26,99 kg/m^2^ (normal), 3 = 27–29,99 kg/m^2^ (overweight), 4 ≥ 30 kg/m^2^ (obese). Individual comparisons are not shown here.  FHC, Finnish HC; FPDAC, Finnish PDAC; HC, healthy control; IHC, Iranian HC; IPDAC, Iranian PDAC; NA, not available; ns, non-significant; PDAC, pancreatic ductal adenocarcinoma. | | | | | |
