# Supplementary figures and images for: Fecal profiling reveals a common microbial signature for pancreatic cancer in Finnish and Iranian cohorts
Source: Gut Pathog. 2025 Apr 16;17:24. doi: 10.1186/s13099-025-00698-0 (PMC12001732; doi:10.1186/s13099-025-00698-0)

## Slide 1
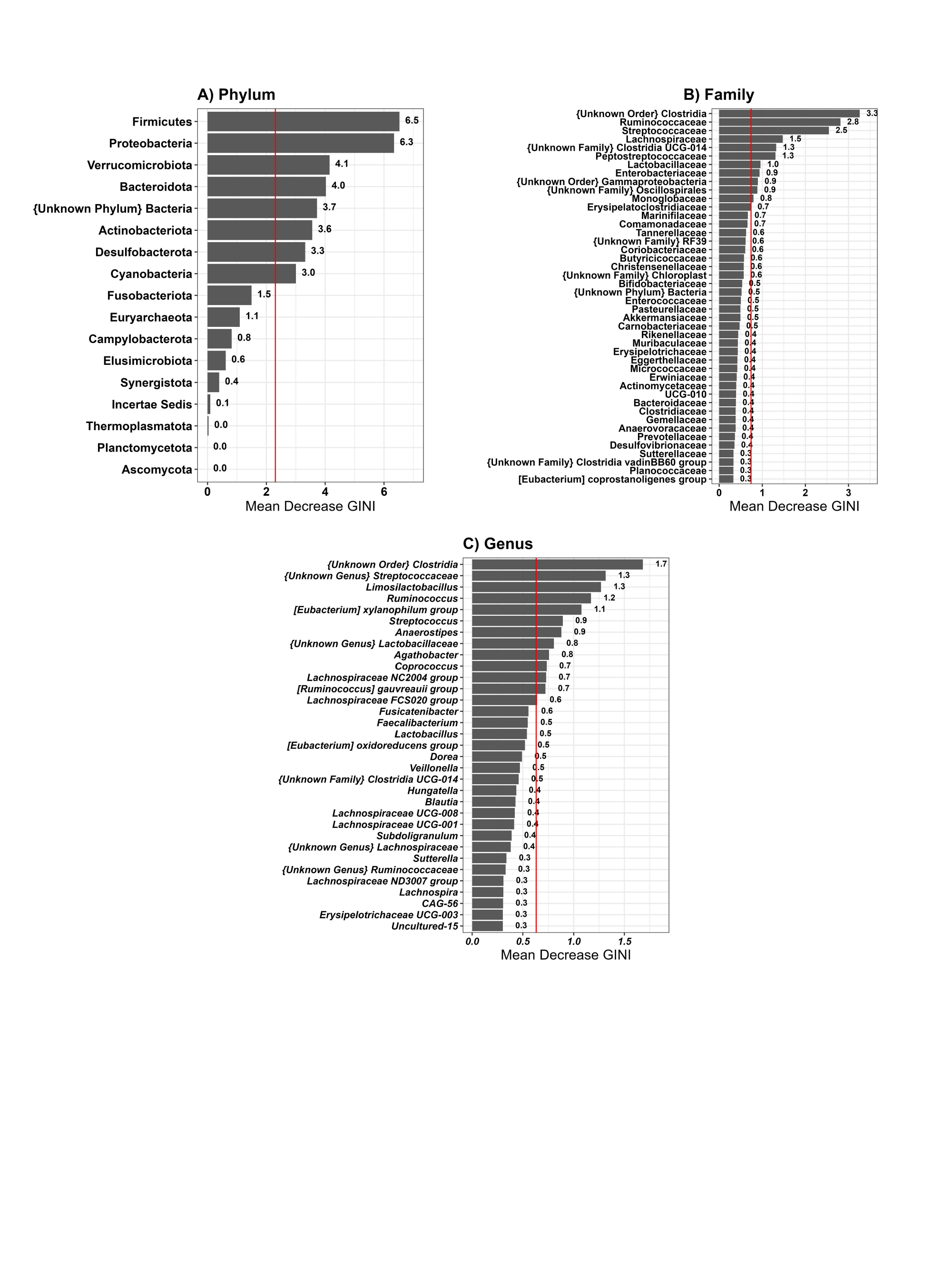

Supplement: Supplementary file 7 — Additional file 7: Figure S2. Mean Decrease Gini. Variable Importance Analysis for PDAC prediction using Mean Decrease Gini across the taxonomic ranks phylum, family, and genus. [file 13099_2025_698_MOESM7_ESM.pptx]
