# Supplementary material for: Fecal profiling reveals a common microbial signature for pancreatic cancer in Finnish and Iranian cohorts
Source: Gut Pathog. 2025 Apr 16;17:24. doi: 10.1186/s13099-025-00698-0 (PMC12001732; doi:10.1186/s13099-025-00698-0)
Supplement: Supplementary file 8 — Additional file 8: Table S6. Variable selection for PDAC prediction. Variable selection for PDAC prediction based on phylum, family, and genus variables using logistic regression. [file 13099_2025_698_MOESM8_ESM.docx]

**Supplementary Table S6. Variable selection for PDAC prediction**

**Table S6A.** Variable selection for PDAC prediction based on phylum variables using Logistic Regression

| **Variables** | **AUC (95% CI)** | **SE (95% CI)** | **SP (95% CI)** | **PPV (95% CI)** | **NPV (95% CI)** | **ACC (95% CI)** |
| --- | --- | --- | --- | --- | --- | --- |
| Euryarchaeota | 0.51 (0.43, 0.59) | 0.97 (0.85, 0.99) | 0.09 (0.03, 0.22) | 0.50 (0.38, 0.62) | 0.75 (0.30, 0.95) | 0.50 (0.38, 0.62) |
| Thermoplasmatota | 0.52 (0.49, 0.54) | 0.03 (0.01, 0.15) | 1.00 (0.90, 1.00) | 1.00 (0.21, 1.00) | 0.52 (0.40, 0.64) | 0.51 (0.39, 0.64) |
| {Unknown Phylum} Bacteria | 0.49 (0.36, 0.63) | 0.79 (0.62, 0.89) | 0.31 (0.19, 0.48) | 0.52 (0.39, 0.65) | 0.61 (0.39, 0.80) | 0.51 (0.39, 0.64) |
| Actinobacteriota | 0.57 (0.44, 0.71) | 0.33 (0.20, 0.50) | 0.83 (0.67, 0.92) | 0.65 (0.41, 0.83) | 0.57 (0.43, 0.69) | 0.54 (0.42, 0.67) |
| **Bacteroidota** | **0.70 (0.58, 0.83)** | **0.52 (0.35, 0.67)** | **0.83 (0.67, 0.92)** | **0.74 (0.54, 0.87)** | **0.64 (0.50, 0.77)** | **0.68 (0.55, 0.78)** |
| Campylobacterota | 0.65 (0.56, 0.74) | 0.36 (0.22, 0.53) | 0.94 (0.81, 0.98) | 0.86 (0.60, 0.96) | 0.61 (0.48, 0.73) | 0.51 (0.39, 0.64) |
| **Cyanobacteria** | **0.70 (0.58, 0.83)** | **0.82 (0.66, 0.91)** | **0.57 (0.41, 0.72)** | **0.64 (0.49, 0.77)** | **0.77 (0.58, 0.89)** | **0.66 (0.54, 0.77)** |
| Desulfobacterota | 0.49 (0.35, 0.63) | 0.48 (0.33, 0.65) | 0.60 (0.44, 0.74) | 0.53 (0.36, 0.70) | 0.55 (0.40, 0.70) | 0.50 (0.38, 0.62) |
| Elusimicrobiota | 0.50 (0.50, 0.50) | 1.00 (0.90, 1.00) | 0.00 (0.00, 0.10) | 0.49 (0.37, 0.60) | NA (NA, NA) | 0.49 (0.36, 0.61) |
| **Firmicutes** | **0.76 (0.65, 0.87)** | **0.88 (0.73, 0.95)** | **0.54 (0.38, 0.70)** | **0.64 (0.50, 0.77)** | **0.83 (0.63, 0.93)** | **0.71 (0.58, 0.81)** |
| Fusobacteriota | 0.57 (0.44, 0.69) | 0.30 (0.17, 0.47) | 0.94 (0.81, 0.98) | 0.83 (0.55, 0.95) | 0.59 (0.46, 0.71) | 0.60 (0.48, 0.72) |
| Planctomycetota | 0.50 (0.50, 0.50) | 1.00 (0.90, 1.00) | 0.00 (0.00, 0.10) | 0.49 (0.37, 0.60) | NA (NA, NA) | 0.51 (0.39, 0.64) |
| Proteobacteria | 0.63 (0.50, 0.77) | 0.58 (0.41, 0.73) | 0.69 (0.52, 0.81) | 0.63 (0.46, 0.78) | 0.63 (0.47, 0.77) | 0.62 (0.49, 0.73) |
| Synergistota | 0.60 (0.51, 0.68) | 0.24 (0.13, 0.41) | 0.97 (0.85, 0.99) | 0.89 (0.57, 0.98) | 0.58 (0.45, 0.69) | 0.59 (0.46, 0.71) |
| Verrucomicrobiota | 0.46 (0.32, 0.60) | 0.21 (0.11, 0.38) | 0.91 (0.78, 0.97) | 0.70 (0.40, 0.89) | 0.55 (0.42, 0.67) | 0.57 (0.45, 0.69) |
| Ascomycota | 0.50 (0.50, 0.50) | 1.00 (0.90, 1.00) | 0.00 (0.00, 0.10) | 0.49 (0.37, 0.60) | NA (NA, NA) | 0.51 (0.39, 0.64) |
| Incertae Sedis | 0.46 (0.40, 0.52) | 1.00 (0.90, 1.00) | 0.00 (0.00, 0.10) | 0.49 (0.37, 0.60) | NA (NA, NA) | 0.49 (0.36, 0.61) |
| AUC (Area Under the Curve), SE (Sensitivity), SP (Specificity), PPV (Positive Predictive Value), NPV (Negative Predictive Value). | | | | | | |

**Table S6B.** Variable selection for PDAC prediction based on family variables using Logistic Regression

| **Variables** | **AUC (95% CI)** | **SE (95% CI)** | **SP (95% CI)** | **PPV (95% CI)** | **NPV (95% CI)** | **ACC (95% CI)** |
| --- | --- | --- | --- | --- | --- | --- |
| Methanobacteriaceae | 0.51 (0.43, 0.59) | 0.97 (0.85, 0.99) | 0.09 (0.03, 0.22) | 0.50 (0.38, 0.62) | 0.75 (0.30, 0.95) | 0.50 (0.38, 0.62) |
| Methanomethylophilaceae | 0.52 (0.49, 0.54) | 0.03 (0.01, 0.15) | 1.00 (0.90, 1.00) | 1.00 (0.21, 1.00) | 0.52 (0.40, 0.64) | 0.51 (0.39, 0.64) |
| {Unknown Phylum} Bacteria | 0.49 (0.36, 0.63) | 0.79 (0.62, 0.89) | 0.31 (0.19, 0.48) | 0.52 (0.39, 0.65) | 0.61 (0.39, 0.80) | 0.51 (0.39, 0.64) |
| {Unknown Order} Actinobacteria | 0.50 (0.50, 0.50) | 1.00 (0.90, 1.00) | 0.00 (0.00, 0.10) | 0.49 (0.37, 0.60) | NA (NA, NA) | 0.49 (0.36, 0.61) |
| Actinomycetaceae | 0.52 (0.42, 0.63) | 0.24 (0.13, 0.41) | 0.86 (0.71, 0.94) | 0.62 (0.36, 0.82) | 0.55 (0.42, 0.67) | 0.51 (0.39, 0.64) |
| Bifidobacteriaceae | 0.58 (0.44, 0.71) | 0.39 (0.25, 0.56) | 0.74 (0.58, 0.86) | 0.59 (0.39, 0.77) | 0.57 (0.42, 0.70) | 0.56 (0.43, 0.68) |
| Corynebacteriaceae | 0.53 (0.46, 0.61) | 0.15 (0.07, 0.31) | 0.91 (0.78, 0.97) | 0.63 (0.31, 0.86) | 0.53 (0.41, 0.65) | 0.54 (0.42, 0.67) |
| Micrococcaceae | 0.47 (0.42, 0.53) | 0.03 (0.01, 0.15) | 1.00 (0.90, 1.00) | 1.00 (0.21, 1.00) | 0.52 (0.40, 0.64) | 0.51 (0.39, 0.64) |
| Atopobiaceae | 0.46 (0.33, 0.58) | 0.18 (0.09, 0.34) | 0.91 (0.78, 0.97) | 0.67 (0.35, 0.88) | 0.54 (0.42, 0.66) | 0.54 (0.42, 0.67) |
| Coriobacteriaceae | 0.45 (0.31, 0.60) | 0.18 (0.09, 0.34) | 0.91 (0.78, 0.97) | 0.67 (0.35, 0.88) | 0.54 (0.42, 0.66) | 0.54 (0.42, 0.67) |
| Coriobacteriales Incertae Sedis | 0.38 (0.25, 0.51) | 1.00 (0.90, 1.00) | 0.03 (0.01, 0.15) | 0.49 (0.38, 0.61) | 1.00 (0.21, 1.00) | 0.50 (0.38, 0.62) |
| Eggerthellaceae | 0.33 (0.20, 0.46) | 1.00 (0.90, 1.00) | 0.00 (0.00, 0.10) | 0.49 (0.37, 0.60) | NA (NA, NA) | 0.35 (0.24, 0.48) |
| Uncultured-1 | 0.54 (0.43, 0.64) | 0.85 (0.69, 0.93) | 0.26 (0.14, 0.42) | 0.52 (0.39, 0.65) | 0.64 (0.39, 0.84) | 0.51 (0.39, 0.64) |
| {Unknown Family} Bacteroidales | 0.46 (0.33, 0.60) | 0.36 (0.22, 0.53) | 0.74 (0.58, 0.86) | 0.57 (0.37, 0.76) | 0.55 (0.41, 0.69) | 0.56 (0.43, 0.68) |
| Bacteroidaceae | 0.66 (0.53, 0.79) | 0.73 (0.56, 0.85) | 0.60 (0.44, 0.74) | 0.63 (0.47, 0.77) | 0.70 (0.52, 0.83) | 0.65 (0.52, 0.76) |
| Barnesiellaceae | 0.51 (0.36, 0.65) | 0.36 (0.22, 0.53) | 0.89 (0.74, 0.95) | 0.75 (0.51, 0.90) | 0.60 (0.46, 0.72) | 0.63 (0.51, 0.75) |
| Marinifilaceae | 0.58 (0.45, 0.72) | 0.67 (0.50, 0.80) | 0.57 (0.41, 0.72) | 0.59 (0.43, 0.74) | 0.65 (0.47, 0.79) | 0.62 (0.49, 0.73) |
| **Muribaculaceae** | **0.73 (0.61, 0.85)** | **0.82 (0.66, 0.91)** | **0.69 (0.52, 0.81)** | **0.71 (0.55, 0.83)** | **0.80 (0.63, 0.90)** | **0.66 (0.54, 0.77)** |
| Porphyromonadaceae | 0.61 (0.49, 0.72) | 0.30 (0.17, 0.47) | 0.94 (0.81, 0.98) | 0.83 (0.55, 0.95) | 0.59 (0.46, 0.71) | 0.59 (0.46, 0.71) |
| **Prevotellaceae** | **0.76 (0.64, 0.88)** | **0.64 (0.47, 0.78)** | **0.86 (0.71, 0.94)** | **0.81 (0.62, 0.91)** | **0.71 (0.56, 0.83)** | **0.51 (0.39, 0.64)** |
| Rikenellaceae | 0.36 (0.23, 0.50) | 0.03 (0.01, 0.15) | 0.97 (0.85, 0.99) | 0.50 (0.09, 0.91) | 0.52 (0.40, 0.63) | 0.51 (0.39, 0.64) |
| Tannerellaceae | 0.54 (0.39, 0.68) | 0.39 (0.25, 0.56) | 0.74 (0.58, 0.86) | 0.59 (0.39, 0.77) | 0.57 (0.42, 0.70) | 0.57 (0.45, 0.69) |
| Uncultured-2 | 0.51 (0.42, 0.60) | 0.18 (0.09, 0.34) | 0.91 (0.78, 0.97) | 0.67 (0.35, 0.88) | 0.54 (0.42, 0.66) | 0.51 (0.39, 0.64) |
| Flavobacteriaceae | 0.56 (0.48, 0.64) | 1.00 (0.90, 1.00) | 0.14 (0.06, 0.29) | 0.52 (0.40, 0.64) | 1.00 (0.57, 1.00) | 0.54 (0.42, 0.67) |
| Lentimicrobiaceae | 0.50 (0.50, 0.50) | 1.00 (0.90, 1.00) | 0.00 (0.00, 0.10) | 0.49 (0.37, 0.60) | NA (NA, NA) | 0.51 (0.39, 0.64) |
| Sphingobacteriaceae | 0.50 (0.50, 0.50) | 1.00 (0.90, 1.00) | 0.00 (0.00, 0.10) | 0.49 (0.37, 0.60) | NA (NA, NA) | 0.49 (0.36, 0.61) |
| Campylobacteraceae | 0.65 (0.56, 0.74) | 0.36 (0.22, 0.53) | 0.94 (0.81, 0.98) | 0.86 (0.60, 0.96) | 0.61 (0.48, 0.73) | 0.51 (0.39, 0.64) |
| Helicobacteraceae | 0.50 (0.50, 0.50) | 1.00 (0.90, 1.00) | 0.00 (0.00, 0.10) | 0.49 (0.37, 0.60) | NA (NA, NA) | 0.51 (0.39, 0.64) |
| {Unknown Family} Chloroplast | 0.33 (0.21, 0.44) | 1.00 (0.90, 1.00) | 0.00 (0.00, 0.10) | 0.49 (0.37, 0.60) | NA (NA, NA) | 0.49 (0.36, 0.61) |
| {Unknown Family} Gastranaerophilales | 0.68 (0.56, 0.81) | 0.82 (0.66, 0.91) | 0.49 (0.33, 0.64) | 0.60 (0.45, 0.73) | 0.74 (0.54, 0.87) | 0.63 (0.51, 0.75) |
| Desulfovibrionaceae | 0.49 (0.35, 0.63) | 0.48 (0.33, 0.65) | 0.60 (0.44, 0.74) | 0.53 (0.36, 0.70) | 0.55 (0.40, 0.70) | 0.50 (0.38, 0.62) |
| Elusimicrobiaceae | 0.50 (0.50, 0.50) | 1.00 (0.90, 1.00) | 0.00 (0.00, 0.10) | 0.49 (0.37, 0.60) | NA (NA, NA) | 0.49 (0.36, 0.61) |
| {Unknown Class} Firmicutes | 0.34 (0.23, 0.46) | 0.03 (0.01, 0.15) | 0.97 (0.85, 0.99) | 0.50 (0.09, 0.91) | 0.52 (0.40, 0.63) | 0.51 (0.39, 0.64) |
| {Unknown Order} Bacilli | 0.49 (0.46, 0.51) | 1.00 (0.90, 1.00) | 0.00 (0.00, 0.10) | 0.49 (0.37, 0.60) | NA (NA, NA) | 0.50 (0.38, 0.62) |
| Acholeplasmataceae | 0.51 (0.45, 0.58) | 0.97 (0.85, 0.99) | 0.09 (0.03, 0.22) | 0.50 (0.38, 0.62) | 0.75 (0.30, 0.95) | 0.49 (0.36, 0.61) |
| Bacillaceae | 0.50 (0.50, 0.50) | 1.00 (0.90, 1.00) | 0.00 (0.00, 0.10) | 0.49 (0.37, 0.60) | NA (NA, NA) | 0.49 (0.36, 0.61) |
| Planococcaceae | 0.47 (0.43, 0.51) | 1.00 (0.90, 1.00) | 0.00 (0.00, 0.10) | 0.49 (0.37, 0.60) | NA (NA, NA) | 0.49 (0.36, 0.61) |
| **Erysipelatoclostridiaceae** | **0.73 (0.61, 0.86)** | **0.73 (0.56, 0.85)** | **0.74 (0.58, 0.86)** | **0.73 (0.56, 0.85)** | **0.74 (0.58, 0.86)** | **0.74 (0.61, 0.83)** |
| Erysipelotrichaceae | 0.54 (0.40, 0.68) | 0.88 (0.73, 0.95) | 0.29 (0.16, 0.45) | 0.54 (0.41, 0.66) | 0.71 (0.45, 0.88) | 0.53 (0.40, 0.65) |
| {Unknown Family} Izemoplasmatales | 0.48 (0.37, 0.60) | 0.09 (0.03, 0.24) | 1.00 (0.90, 1.00) | 1.00 (0.44, 1.00) | 0.54 (0.42, 0.65) | 0.51 (0.39, 0.64) |
| {Unknown Family} Lactobacillales | 0.55 (0.44, 0.65) | 0.27 (0.15, 0.44) | 0.86 (0.71, 0.94) | 0.64 (0.39, 0.84) | 0.56 (0.42, 0.68) | 0.57 (0.45, 0.69) |
| Aerococcaceae | 0.50 (0.50, 0.50) | 1.00 (0.90, 1.00) | 0.00 (0.00, 0.10) | 0.49 (0.37, 0.60) | NA (NA, NA) | 0.51 (0.39, 0.64) |
| Carnobacteriaceae | 0.46 (0.34, 0.58) | 0.15 (0.07, 0.31) | 0.94 (0.81, 0.98) | 0.71 (0.36, 0.92) | 0.54 (0.42, 0.66) | 0.51 (0.39, 0.64) |
| **Enterococcaceae** | **0.76 (0.67, 0.86)** | **0.58 (0.41, 0.73)** | **0.97 (0.85, 0.99)** | **0.95 (0.76, 0.99)** | **0.71 (0.57, 0.82)** | **0.71 (0.58, 0.81)** |
| Lactobacillaceae | 0.48 (0.34, 0.62) | 0.61 (0.44, 0.75) | 0.46 (0.30, 0.62) | 0.51 (0.36, 0.66) | 0.55 (0.38, 0.72) | 0.49 (0.36, 0.61) |
| Streptococcaceae | 0.52 (0.38, 0.66) | 0.91 (0.76, 0.97) | 0.23 (0.12, 0.39) | 0.53 (0.40, 0.65) | 0.73 (0.43, 0.90) | 0.53 (0.40, 0.65) |
| Uncultured-3 | 0.53 (0.49, 0.57) | 0.06 (0.02, 0.20) | 1.00 (0.90, 1.00) | 1.00 (0.34, 1.00) | 0.53 (0.41, 0.65) | 0.54 (0.42, 0.67) |
| Paenibacillaceae | 0.50 (0.50, 0.50) | 1.00 (0.90, 1.00) | 0.00 (0.00, 0.10) | 0.49 (0.37, 0.60) | NA (NA, NA) | 0.51 (0.39, 0.64) |
| **{Unknown Family} RF39** | **0.72 (0.61, 0.84)** | **0.73 (0.56, 0.85)** | **0.71 (0.55, 0.84)** | **0.71 (0.54, 0.83)** | **0.74 (0.57, 0.85)** | **0.71 (0.58, 0.81)** |
| Gemellaceae | 0.49 (0.40, 0.59) | 0.97 (0.85, 0.99) | 0.09 (0.03, 0.22) | 0.50 (0.38, 0.62) | 0.75 (0.30, 0.95) | 0.51 (0.39, 0.64) |
| Staphylococcaceae | 0.52 (0.47, 0.57) | 0.06 (0.02, 0.20) | 1.00 (0.90, 1.00) | 1.00 (0.34, 1.00) | 0.53 (0.41, 0.65) | 0.53 (0.40, 0.65) |
| {Unknown Order} Clostridia | 0.67 (0.54, 0.80) | 0.42 (0.27, 0.59) | 0.91 (0.78, 0.97) | 0.82 (0.59, 0.94) | 0.63 (0.49, 0.75) | 0.66 (0.54, 0.77) |
| Caldicoprobacteraceae | 0.45 (0.37, 0.53) | 1.00 (0.90, 1.00) | 0.00 (0.00, 0.10) | 0.49 (0.37, 0.60) | NA (NA, NA) | 0.49 (0.36, 0.61) |
| Christensenellaceae | 0.67 (0.54, 0.80) | 0.61 (0.44, 0.75) | 0.71 (0.55, 0.84) | 0.67 (0.49, 0.81) | 0.66 (0.50, 0.79) | 0.65 (0.52, 0.76) |
| **{Unknown Family} Clostridia UCG-014** | **0.84 (0.74, 0.94)** | **0.73 (0.56, 0.85)** | **0.86 (0.71, 0.94)** | **0.83 (0.65, 0.92)** | **0.77 (0.62, 0.87)** | **0.78 (0.66, 0.87)** |
| {Unknown Family} Clostridia vadinBB60 group | 0.26 (0.14, 0.38) | 0.03 (0.01, 0.15) | 0.97 (0.85, 0.99) | 0.50 (0.09, 0.91) | 0.52 (0.40, 0.63) | 0.51 (0.39, 0.64) |
| Clostridiaceae | 0.33 (0.21, 0.46) | 1.00 (0.90, 1.00) | 0.00 (0.00, 0.10) | 0.49 (0.37, 0.60) | NA (NA, NA) | 0.49 (0.36, 0.61) |
| Anaerofustaceae | 0.52 (0.45, 0.58) | 0.09 (0.03, 0.24) | 0.94 (0.81, 0.98) | 0.60 (0.23, 0.88) | 0.52 (0.40, 0.64) | 0.51 (0.39, 0.64) |
| Eubacteriaceae | 0.50 (0.46, 0.54) | 0.03 (0.01, 0.15) | 0.97 (0.85, 0.99) | 0.50 (0.09, 0.91) | 0.52 (0.40, 0.63) | 0.50 (0.38, 0.62) |
| Defluviitaleaceae | 0.54 (0.46, 0.62) | 0.97 (0.85, 0.99) | 0.14 (0.06, 0.29) | 0.52 (0.39, 0.64) | 0.83 (0.44, 0.97) | 0.54 (0.42, 0.67) |
| Lachnospiraceae | 0.66 (0.53, 0.80) | 0.58 (0.41, 0.73) | 0.77 (0.61, 0.88) | 0.70 (0.52, 0.84) | 0.66 (0.51, 0.78) | 0.68 (0.55, 0.78) |
| Monoglobaceae | 0.48 (0.34, 0.62) | 0.30 (0.17, 0.47) | 0.77 (0.61, 0.88) | 0.56 (0.34, 0.75) | 0.54 (0.40, 0.67) | 0.53 (0.40, 0.65) |
| {Unknown Family} Oscillospirales | 0.57 (0.43, 0.71) | 0.42 (0.27, 0.59) | 0.80 (0.64, 0.90) | 0.67 (0.45, 0.83) | 0.60 (0.45, 0.72) | 0.59 (0.46, 0.71) |
| [Clostridium] methylpentosum group | 0.47 (0.35, 0.59) | 0.82 (0.66, 0.91) | 0.20 (0.10, 0.36) | 0.49 (0.36, 0.62) | 0.54 (0.29, 0.77) | 0.49 (0.36, 0.61) |
| [Eubacterium] coprostanoligenes group | 0.53 (0.39, 0.67) | 0.27 (0.15, 0.44) | 0.89 (0.74, 0.95) | 0.69 (0.42, 0.87) | 0.56 (0.43, 0.69) | 0.51 (0.39, 0.64) |
| **Butyricicoccaceae** | **0.74 (0.62, 0.86)** | **0.85 (0.69, 0.93)** | **0.63 (0.46, 0.77)** | **0.68 (0.53, 0.80)** | **0.81 (0.63, 0.92)** | **0.72 (0.60, 0.82)** |
| Ethanoligenenaceae | 0.50 (0.40, 0.59) | 0.97 (0.85, 0.99) | 0.06 (0.02, 0.19) | 0.49 (0.37, 0.61) | 0.67 (0.21, 0.94) | 0.49 (0.36, 0.61) |
| Hungateiclostridiaceae | 0.50 (0.38, 0.63) | 0.64 (0.47, 0.78) | 0.43 (0.28, 0.59) | 0.51 (0.36, 0.66) | 0.56 (0.37, 0.72) | 0.53 (0.40, 0.65) |
| Oscillospiraceae | 0.53 (0.39, 0.67) | 0.76 (0.59, 0.87) | 0.31 (0.19, 0.48) | 0.51 (0.37, 0.64) | 0.58 (0.36, 0.77) | 0.51 (0.39, 0.64) |
| Ruminococcaceae | 0.66 (0.53, 0.80) | 0.48 (0.33, 0.65) | 0.86 (0.71, 0.94) | 0.76 (0.55, 0.89) | 0.64 (0.50, 0.76) | 0.68 (0.55, 0.78) |
| UCG-010 | 0.31 (0.18, 0.44) | 1.00 (0.90, 1.00) | 0.00 (0.00, 0.10) | 0.49 (0.37, 0.60) | NA (NA, NA) | 0.35 (0.24, 0.48) |
| UCG-011 | 0.54 (0.47, 0.61) | 0.94 (0.80, 0.98) | 0.14 (0.06, 0.29) | 0.51 (0.39, 0.63) | 0.71 (0.36, 0.92) | 0.51 (0.39, 0.64) |
| Uncultured-4 | 0.53 (0.42, 0.64) | 0.33 (0.20, 0.50) | 0.80 (0.64, 0.90) | 0.61 (0.39, 0.80) | 0.56 (0.42, 0.69) | 0.56 (0.43, 0.68) |
| Peptococcaceae | 0.40 (0.26, 0.54) | 0.06 (0.02, 0.20) | 1.00 (0.90, 1.00) | 1.00 (0.34, 1.00) | 0.53 (0.41, 0.65) | 0.53 (0.40, 0.65) |
| Anaerovoracaceae | 0.43 (0.29, 0.57) | 0.27 (0.15, 0.44) | 0.80 (0.64, 0.90) | 0.56 (0.33, 0.77) | 0.54 (0.41, 0.67) | 0.53 (0.40, 0.65) |
| **Family XI** | **0.71 (0.60, 0.81)** | **0.58 (0.41, 0.73)** | **0.83 (0.67, 0.92)** | **0.76 (0.57, 0.89)** | **0.67 (0.53, 0.80)** | **0.71 (0.58, 0.81)** |
| Peptostreptococcaceae | 0.64 (0.50, 0.77) | 0.70 (0.53, 0.83) | 0.57 (0.41, 0.72) | 0.61 (0.45, 0.74) | 0.67 (0.49, 0.81) | 0.63 (0.51, 0.75) |
| {Unknown Family} Uncultured | 0.54 (0.45, 0.62) | 0.88 (0.73, 0.95) | 0.20 (0.10, 0.36) | 0.51 (0.38, 0.63) | 0.64 (0.35, 0.85) | 0.53 (0.40, 0.65) |
| Desulfurisporaceae | 0.50 (0.50, 0.50) | 1.00 (0.90, 1.00) | 0.00 (0.00, 0.10) | 0.49 (0.37, 0.60) | NA (NA, NA) | 0.51 (0.39, 0.64) |
| {Unknown Family} DTU014 | 0.52 (0.42, 0.62) | 0.09 (0.03, 0.24) | 0.97 (0.85, 0.99) | 0.75 (0.30, 0.95) | 0.53 (0.41, 0.65) | 0.54 (0.42, 0.67) |
| {Unknown Family} MBA03 | 0.42 (0.33, 0.51) | 0.06 (0.02, 0.20) | 1.00 (0.90, 1.00) | 1.00 (0.34, 1.00) | 0.53 (0.41, 0.65) | 0.53 (0.40, 0.65) |
| {Unknown Order} Negativicutes | 0.58 (0.47, 0.68) | 0.33 (0.20, 0.50) | 0.83 (0.67, 0.92) | 0.65 (0.41, 0.83) | 0.57 (0.43, 0.69) | 0.59 (0.46, 0.71) |
| Acidaminococcaceae | 0.59 (0.46, 0.73) | 0.52 (0.35, 0.67) | 0.71 (0.55, 0.84) | 0.63 (0.44, 0.78) | 0.61 (0.46, 0.74) | 0.51 (0.39, 0.64) |
| Selenomonadaceae | 0.43 (0.35, 0.51) | 1.00 (0.90, 1.00) | 0.00 (0.00, 0.10) | 0.49 (0.37, 0.60) | NA (NA, NA) | 0.49 (0.36, 0.61) |
| Veillonellaceae | 0.41 (0.27, 0.55) | 0.91 (0.76, 0.97) | 0.14 (0.06, 0.29) | 0.50 (0.38, 0.62) | 0.63 (0.31, 0.86) | 0.49 (0.36, 0.61) |
| Syntrophomonadaceae | 0.50 (0.50, 0.50) | 1.00 (0.90, 1.00) | 0.00 (0.00, 0.10) | 0.49 (0.37, 0.60) | NA (NA, NA) | 0.51 (0.39, 0.64) |
| {Unknown Order} Uncultured | 0.47 (0.41, 0.54) | 0.03 (0.01, 0.15) | 1.00 (0.90, 1.00) | 1.00 (0.21, 1.00) | 0.52 (0.40, 0.64) | 0.51 (0.39, 0.64) |
| Fusobacteriaceae | 0.56 (0.44, 0.68) | 0.27 (0.15, 0.44) | 0.94 (0.81, 0.98) | 0.82 (0.52, 0.95) | 0.58 (0.45, 0.70) | 0.59 (0.46, 0.71) |
| Leptotrichiaceae | 0.48 (0.46, 0.51) | 1.00 (0.90, 1.00) | 0.00 (0.00, 0.10) | 0.49 (0.37, 0.60) | NA (NA, NA) | 0.49 (0.36, 0.61) |
| WD2101 soil group | 0.50 (0.50, 0.50) | 1.00 (0.90, 1.00) | 0.00 (0.00, 0.10) | 0.49 (0.37, 0.60) | NA (NA, NA) | 0.51 (0.39, 0.64) |
| {Unknown Class} Proteobacteria | 0.50 (0.50, 0.50) | 1.00 (0.90, 1.00) | 0.00 (0.00, 0.10) | 0.49 (0.37, 0.60) | NA (NA, NA) | 0.49 (0.36, 0.61) |
| Uncultured-5 | 0.31 (0.18, 0.44) | 0.18 (0.09, 0.34) | 0.86 (0.71, 0.94) | 0.55 (0.28, 0.79) | 0.53 (0.40, 0.65) | 0.53 (0.40, 0.65) |
| Mitochondria | 0.50 (0.50, 0.50) | 1.00 (0.90, 1.00) | 0.00 (0.00, 0.10) | 0.49 (0.37, 0.60) | NA (NA, NA) | 0.49 (0.36, 0.61) |
| {Unknown Order} Gammaproteobacteria | 0.68 (0.56, 0.81) | 0.61 (0.44, 0.75) | 0.86 (0.71, 0.94) | 0.80 (0.61, 0.91) | 0.70 (0.55, 0.81) | 0.69 (0.57, 0.80) |
| Alcaligenaceae | 0.53 (0.49, 0.57) | 0.06 (0.02, 0.20) | 1.00 (0.90, 1.00) | 1.00 (0.34, 1.00) | 0.53 (0.41, 0.65) | 0.53 (0.40, 0.65) |
| Burkholderiaceae | 0.50 (0.50, 0.50) | 1.00 (0.90, 1.00) | 0.00 (0.00, 0.10) | 0.49 (0.37, 0.60) | NA (NA, NA) | 0.51 (0.39, 0.64) |
| Comamonadaceae | 0.66 (0.56, 0.75) | 0.39 (0.25, 0.56) | 0.94 (0.81, 0.98) | 0.87 (0.62, 0.96) | 0.62 (0.49, 0.74) | 0.68 (0.55, 0.78) |
| Neisseriaceae | 0.49 (0.44, 0.53) | 1.00 (0.90, 1.00) | 0.00 (0.00, 0.10) | 0.49 (0.37, 0.60) | NA (NA, NA) | 0.50 (0.38, 0.62) |
| Oxalobacteraceae | 0.59 (0.48, 0.70) | 0.94 (0.80, 0.98) | 0.26 (0.14, 0.42) | 0.54 (0.42, 0.67) | 0.82 (0.52, 0.95) | 0.59 (0.46, 0.71) |
| Sutterellaceae | 0.45 (0.31, 0.58) | 0.09 (0.03, 0.24) | 0.94 (0.81, 0.98) | 0.60 (0.23, 0.88) | 0.52 (0.40, 0.64) | 0.51 (0.39, 0.64) |
| {Unknown Family} Enterobacterales | 0.63 (0.53, 0.74) | 0.42 (0.27, 0.59) | 0.86 (0.71, 0.94) | 0.74 (0.51, 0.88) | 0.61 (0.47, 0.74) | 0.63 (0.51, 0.75) |
| Alteromonadaceae | 0.50 (0.50, 0.50) | 1.00 (0.90, 1.00) | 0.00 (0.00, 0.10) | 0.49 (0.37, 0.60) | NA (NA, NA) | 0.51 (0.39, 0.64) |
| **Enterobacteriaceae** | **0.74 (0.61, 0.86)** | **0.64 (0.47, 0.78)** | **0.86 (0.71, 0.94)** | **0.81 (0.62, 0.91)** | **0.71 (0.56, 0.83)** | **0.72 (0.60, 0.82)** |
| Erwiniaceae | 0.58 (0.49, 0.67) | 0.24 (0.13, 0.41) | 0.91 (0.78, 0.97) | 0.73 (0.43, 0.90) | 0.56 (0.43, 0.68) | 0.59 (0.46, 0.71) |
| Hafniaceae | 0.52 (0.45, 0.58) | 0.06 (0.02, 0.20) | 1.00 (0.90, 1.00) | 1.00 (0.34, 1.00) | 0.53 (0.41, 0.65) | 0.54 (0.42, 0.67) |
| Morganellaceae | 0.48 (0.46, 0.51) | 1.00 (0.90, 1.00) | 0.00 (0.00, 0.10) | 0.49 (0.37, 0.60) | NA (NA, NA) | 0.47 (0.35, 0.60) |
| Pasteurellaceae | 0.44 (0.31, 0.58) | 0.03 (0.01, 0.15) | 1.00 (0.90, 1.00) | 1.00 (0.21, 1.00) | 0.52 (0.40, 0.64) | 0.53 (0.40, 0.65) |
| Succinivibrionaceae | 0.47 (0.39, 0.56) | 1.00 (0.90, 1.00) | 0.00 (0.00, 0.10) | 0.49 (0.37, 0.60) | NA (NA, NA) | 0.47 (0.35, 0.60) |
| Vibrionaceae | 0.57 (0.50, 0.65) | 0.18 (0.09, 0.34) | 0.97 (0.85, 0.99) | 0.86 (0.49, 0.97) | 0.56 (0.43, 0.67) | 0.59 (0.46, 0.71) |
| Yersiniaceae | 0.59 (0.52, 0.67) | 0.21 (0.11, 0.38) | 0.97 (0.85, 0.99) | 0.88 (0.53, 0.98) | 0.57 (0.44, 0.68) | 0.60 (0.48, 0.72) |
| Moraxellaceae | 0.53 (0.49, 0.57) | 0.06 (0.02, 0.20) | 1.00 (0.90, 1.00) | 1.00 (0.34, 1.00) | 0.53 (0.41, 0.65) | 0.49 (0.36, 0.61) |
| Pseudomonadaceae | 0.48 (0.41, 0.56) | 1.00 (0.90, 1.00) | 0.00 (0.00, 0.10) | 0.49 (0.37, 0.60) | NA (NA, NA) | 0.49 (0.36, 0.61) |
| Xanthomonadaceae | 0.50 (0.50, 0.50) | 1.00 (0.90, 1.00) | 0.00 (0.00, 0.10) | 0.49 (0.37, 0.60) | NA (NA, NA) | 0.49 (0.36, 0.61) |
| Synergistaceae | 0.60 (0.51, 0.68) | 0.24 (0.13, 0.41) | 0.97 (0.85, 0.99) | 0.89 (0.57, 0.98) | 0.58 (0.45, 0.69) | 0.59 (0.46, 0.71) |
| {Unknown Family} WCHB1-41 | 0.50 (0.50, 0.50) | 1.00 (0.90, 1.00) | 0.00 (0.00, 0.10) | 0.49 (0.37, 0.60) | NA (NA, NA) | 0.51 (0.39, 0.64) |
| VadinBE97 | 0.45 (0.34, 0.56) | 0.12 (0.05, 0.27) | 0.89 (0.74, 0.95) | 0.50 (0.22, 0.78) | 0.52 (0.39, 0.64) | 0.51 (0.39, 0.64) |
| Victivallaceae | 0.41 (0.28, 0.54) | 0.06 (0.02, 0.20) | 0.94 (0.81, 0.98) | 0.50 (0.15, 0.85) | 0.52 (0.40, 0.63) | 0.51 (0.39, 0.64) |
| Puniceicoccaceae | 0.44 (0.34, 0.55) | 1.00 (0.90, 1.00) | 0.00 (0.00, 0.10) | 0.49 (0.37, 0.60) | NA (NA, NA) | 0.46 (0.33, 0.58) |
| Akkermansiaceae | 0.48 (0.34, 0.62) | 0.27 (0.15, 0.44) | 0.83 (0.67, 0.92) | 0.60 (0.36, 0.80) | 0.55 (0.41, 0.67) | 0.53 (0.40, 0.65) |
| Pycnoraceae | 0.50 (0.50, 0.50) | 1.00 (0.90, 1.00) | 0.00 (0.00, 0.10) | 0.49 (0.37, 0.60) | NA (NA, NA) | 0.51 (0.39, 0.64) |
| Xylariaceae | 0.50 (0.50, 0.50) | 1.00 (0.90, 1.00) | 0.00 (0.00, 0.10) | 0.49 (0.37, 0.60) | NA (NA, NA) | 0.51 (0.39, 0.64) |
| {Unknown Class} Incertae Sedis | 0.46 (0.40, 0.52) | 1.00 (0.90, 1.00) | 0.00 (0.00, 0.10) | 0.49 (0.37, 0.60) | NA (NA, NA) | 0.49 (0.36, 0.61) |

**Table S6C.** Variable selection for PDAC prediction based on genus variables using Logistic Regression

| **Variables** | **AUC (95% CI)** | **SE (95% CI)** | **SP (95% CI)** | **PPV (95% CI)** | **NPV (95% CI)** | **ACC (95% CI)** |
| --- | --- | --- | --- | --- | --- | --- |
| Methanobrevibacter | 0.50 (0.42, 0.58) | 0.97 (0.85, 0.99) | 0.06 (0.02, 0.19) | 0.49 (0.37, 0.61) | 0.67 (0.21, 0.94) | 0.50 (0.38, 0.62) |
| Methanosphaera | 0.51 (0.49, 0.54) | 1.00 (0.90, 1.00) | 0.03 (0.01, 0.15) | 0.49 (0.38, 0.61) | 1.00 (0.21, 1.00) | 0.50 (0.38, 0.62) |
| {Unknown Genus} Methanomethylophilaceae | 0.50 (0.50, 0.50) | 1.00 (0.90, 1.00) | 0.00 (0.00, 0.10) | 0.49 (0.37, 0.60) | NA (NA, NA) | 0.51 (0.39, 0.64) |
| Candidatus Methanogranum | 0.50 (0.50, 0.50) | 1.00 (0.90, 1.00) | 0.00 (0.00, 0.10) | 0.49 (0.37, 0.60) | NA (NA, NA) | 0.49 (0.36, 0.61) |
| RumEn M2 | 0.50 (0.50, 0.50) | 1.00 (0.90, 1.00) | 0.00 (0.00, 0.10) | 0.49 (0.37, 0.60) | NA (NA, NA) | 0.49 (0.36, 0.61) |
| {Unknown Phylum} Bacteria | 0.49 (0.36, 0.63) | 0.79 (0.62, 0.89) | 0.31 (0.19, 0.48) | 0.52 (0.39, 0.65) | 0.61 (0.39, 0.80) | 0.51 (0.39, 0.64) |
| {Unknown Order} Actinobacteria | 0.50 (0.50, 0.50) | 1.00 (0.90, 1.00) | 0.00 (0.00, 0.10) | 0.49 (0.37, 0.60) | NA (NA, NA) | 0.49 (0.36, 0.61) |
| Actinomyces | 0.49 (0.39, 0.59) | 0.18 (0.09, 0.34) | 0.86 (0.71, 0.94) | 0.55 (0.28, 0.79) | 0.53 (0.40, 0.65) | 0.51 (0.39, 0.64) |
| Varibaculum | 0.53 (0.49, 0.57) | 0.06 (0.02, 0.20) | 1.00 (0.90, 1.00) | 1.00 (0.34, 1.00) | 0.53 (0.41, 0.65) | 0.51 (0.39, 0.64) |
| Alloscardovia | 0.50 (0.45, 0.56) | 0.06 (0.02, 0.20) | 1.00 (0.90, 1.00) | 1.00 (0.34, 1.00) | 0.53 (0.41, 0.65) | 0.54 (0.42, 0.67) |
| Bifidobacterium | 0.58 (0.44, 0.71) | 0.39 (0.25, 0.56) | 0.74 (0.58, 0.86) | 0.59 (0.39, 0.77) | 0.57 (0.42, 0.70) | 0.56 (0.43, 0.68) |
| Gardnerella | 0.47 (0.43, 0.51) | 1.00 (0.90, 1.00) | 0.00 (0.00, 0.10) | 0.49 (0.37, 0.60) | NA (NA, NA) | 0.49 (0.36, 0.61) |
| Scardovia | 0.52 (0.47, 0.57) | 0.06 (0.02, 0.20) | 1.00 (0.90, 1.00) | 1.00 (0.34, 1.00) | 0.53 (0.41, 0.65) | 0.54 (0.42, 0.67) |
| Corynebacterium | 0.53 (0.46, 0.60) | 0.12 (0.05, 0.27) | 0.94 (0.81, 0.98) | 0.67 (0.30, 0.90) | 0.53 (0.41, 0.65) | 0.54 (0.42, 0.67) |
| Lawsonella | 0.50 (0.50, 0.50) | 1.00 (0.90, 1.00) | 0.00 (0.00, 0.10) | 0.49 (0.37, 0.60) | NA (NA, NA) | 0.51 (0.39, 0.64) |
| Rothia | 0.47 (0.42, 0.53) | 0.03 (0.01, 0.15) | 1.00 (0.90, 1.00) | 1.00 (0.21, 1.00) | 0.52 (0.40, 0.64) | 0.51 (0.39, 0.64) |
| Atopobium | 0.49 (0.42, 0.57) | 0.06 (0.02, 0.20) | 1.00 (0.90, 1.00) | 1.00 (0.34, 1.00) | 0.53 (0.41, 0.65) | 0.54 (0.42, 0.67) |
| Coriobacteriaceae UCG-002 | 0.50 (0.50, 0.50) | 1.00 (0.90, 1.00) | 0.00 (0.00, 0.10) | 0.49 (0.37, 0.60) | NA (NA, NA) | 0.51 (0.39, 0.64) |
| Coriobacteriaceae UCG-003 | 0.50 (0.50, 0.50) | 1.00 (0.90, 1.00) | 0.00 (0.00, 0.10) | 0.49 (0.37, 0.60) | NA (NA, NA) | 0.51 (0.39, 0.64) |
| Libanicoccus | 0.53 (0.46, 0.60) | 1.00 (0.90, 1.00) | 0.06 (0.02, 0.19) | 0.50 (0.38, 0.62) | 1.00 (0.34, 1.00) | 0.51 (0.39, 0.64) |
| Olsenella | 0.46 (0.36, 0.57) | 0.15 (0.07, 0.31) | 0.94 (0.81, 0.98) | 0.71 (0.36, 0.92) | 0.54 (0.42, 0.66) | 0.54 (0.42, 0.67) |
| Uncultured-01 | 0.53 (0.49, 0.57) | 1.00 (0.90, 1.00) | 0.06 (0.02, 0.19) | 0.50 (0.38, 0.62) | 1.00 (0.34, 1.00) | 0.51 (0.39, 0.64) |
| Collinsella | 0.46 (0.32, 0.60) | 0.18 (0.09, 0.34) | 0.91 (0.78, 0.97) | 0.67 (0.35, 0.88) | 0.54 (0.42, 0.66) | 0.54 (0.42, 0.67) |
| Enorma | 0.50 (0.46, 0.54) | 0.03 (0.01, 0.15) | 1.00 (0.90, 1.00) | 1.00 (0.21, 1.00) | 0.52 (0.40, 0.64) | 0.51 (0.39, 0.64) |
| Raoultibacter | 0.50 (0.50, 0.50) | 1.00 (0.90, 1.00) | 0.00 (0.00, 0.10) | 0.49 (0.37, 0.60) | NA (NA, NA) | 0.51 (0.39, 0.64) |
| Uncultured-02 | 0.38 (0.25, 0.51) | 1.00 (0.90, 1.00) | 0.03 (0.01, 0.15) | 0.49 (0.38, 0.61) | 1.00 (0.21, 1.00) | 0.49 (0.36, 0.61) |
| {Unknown Genus} Eggerthellaceae | 0.56 (0.44, 0.69) | 0.73 (0.56, 0.85) | 0.43 (0.28, 0.59) | 0.55 (0.40, 0.68) | 0.63 (0.43, 0.79) | 0.56 (0.43, 0.68) |
| Asaccharobacter | 0.50 (0.37, 0.64) | 0.97 (0.85, 0.99) | 0.14 (0.06, 0.29) | 0.52 (0.39, 0.64) | 0.83 (0.44, 0.97) | 0.53 (0.40, 0.65) |
| CHKCI002 | 0.53 (0.49, 0.57) | 1.00 (0.90, 1.00) | 0.06 (0.02, 0.19) | 0.50 (0.38, 0.62) | 1.00 (0.34, 1.00) | 0.51 (0.39, 0.64) |
| Denitrobacterium | 0.49 (0.46, 0.51) | 1.00 (0.90, 1.00) | 0.00 (0.00, 0.10) | 0.49 (0.37, 0.60) | NA (NA, NA) | 0.49 (0.36, 0.61) |
| DNF00809 | 0.50 (0.50, 0.50) | 1.00 (0.90, 1.00) | 0.00 (0.00, 0.10) | 0.49 (0.37, 0.60) | NA (NA, NA) | 0.51 (0.39, 0.64) |
| Eggerthella | 0.52 (0.39, 0.65) | 0.58 (0.41, 0.73) | 0.54 (0.38, 0.70) | 0.54 (0.38, 0.70) | 0.58 (0.41, 0.73) | 0.54 (0.42, 0.67) |
| Enterorhabdus | 0.52 (0.42, 0.63) | 1.00 (0.90, 1.00) | 0.14 (0.06, 0.29) | 0.52 (0.40, 0.64) | 1.00 (0.57, 1.00) | 0.53 (0.40, 0.65) |
| Enteroscipio | 0.50 (0.44, 0.56) | 0.06 (0.02, 0.20) | 0.97 (0.85, 0.99) | 0.67 (0.21, 0.94) | 0.52 (0.40, 0.64) | 0.51 (0.39, 0.64) |
| Gordonibacter | 0.49 (0.38, 0.60) | 0.15 (0.07, 0.31) | 0.94 (0.81, 0.98) | 0.71 (0.36, 0.92) | 0.54 (0.42, 0.66) | 0.54 (0.42, 0.67) |
| Parvibacter | 0.50 (0.50, 0.50) | 1.00 (0.90, 1.00) | 0.00 (0.00, 0.10) | 0.49 (0.37, 0.60) | NA (NA, NA) | 0.49 (0.36, 0.61) |
| Senegalimassilia | 0.55 (0.43, 0.68) | 0.94 (0.80, 0.98) | 0.20 (0.10, 0.36) | 0.53 (0.40, 0.65) | 0.78 (0.45, 0.94) | 0.53 (0.40, 0.65) |
| Slackia | 0.65 (0.53, 0.77) | 0.79 (0.62, 0.89) | 0.57 (0.41, 0.72) | 0.63 (0.48, 0.76) | 0.74 (0.55, 0.87) | 0.62 (0.49, 0.73) |
| **Uncultured-03** | **0.72 (0.59, 0.84)** | **0.73 (0.56, 0.85)** | **0.66 (0.49, 0.79)** | **0.67 (0.50, 0.80)** | **0.72 (0.55, 0.84)** | **0.65 (0.52, 0.76)** |
| {Unknown Genus} Uncultured-01 | 0.54 (0.43, 0.64) | 0.85 (0.69, 0.93) | 0.26 (0.14, 0.42) | 0.52 (0.39, 0.65) | 0.64 (0.39, 0.84) | 0.51 (0.39, 0.64) |
| {Unknown Family} Bacteroidales | 0.46 (0.33, 0.60) | 0.36 (0.22, 0.53) | 0.74 (0.58, 0.86) | 0.57 (0.37, 0.76) | 0.55 (0.41, 0.69) | 0.56 (0.43, 0.68) |
| Bacteroides | 0.66 (0.53, 0.79) | 0.73 (0.56, 0.85) | 0.60 (0.44, 0.74) | 0.63 (0.47, 0.77) | 0.70 (0.52, 0.83) | 0.65 (0.52, 0.76) |
| Barnesiella | 0.52 (0.38, 0.66) | 0.42 (0.27, 0.59) | 0.77 (0.61, 0.88) | 0.64 (0.43, 0.80) | 0.59 (0.44, 0.72) | 0.57 (0.45, 0.69) |
| Coprobacter | 0.41 (0.28, 0.54) | 0.18 (0.09, 0.34) | 0.89 (0.74, 0.95) | 0.60 (0.31, 0.83) | 0.53 (0.41, 0.66) | 0.53 (0.40, 0.65) |
| Uncultured-04 | 0.37 (0.29, 0.45) | 1.00 (0.90, 1.00) | 0.00 (0.00, 0.10) | 0.49 (0.37, 0.60) | NA (NA, NA) | 0.49 (0.36, 0.61) |
| {Unknown Genus} Marinifilaceae | 0.51 (0.40, 0.62) | 0.18 (0.09, 0.34) | 0.91 (0.78, 0.97) | 0.67 (0.35, 0.88) | 0.54 (0.42, 0.66) | 0.51 (0.39, 0.64) |
| Butyricimonas | 0.46 (0.32, 0.61) | 0.18 (0.09, 0.34) | 1.00 (0.90, 1.00) | 1.00 (0.61, 1.00) | 0.56 (0.44, 0.68) | 0.60 (0.48, 0.72) |
| Odoribacter | 0.61 (0.47, 0.75) | 0.42 (0.27, 0.59) | 0.89 (0.74, 0.95) | 0.78 (0.55, 0.91) | 0.62 (0.48, 0.74) | 0.65 (0.52, 0.76) |
| Sanguibacteroides | 0.57 (0.49, 0.65) | 0.94 (0.80, 0.98) | 0.20 (0.10, 0.36) | 0.53 (0.40, 0.65) | 0.78 (0.45, 0.94) | 0.56 (0.43, 0.68) |
| Uncultured-05 | 0.51 (0.40, 0.62) | 0.12 (0.05, 0.27) | 1.00 (0.90, 1.00) | 1.00 (0.51, 1.00) | 0.55 (0.43, 0.66) | 0.56 (0.43, 0.68) |
| **{Unknown Genus} Muribaculaceae** | **0.79 (0.69, 0.89)** | **0.88 (0.73, 0.95)** | **0.69 (0.52, 0.81)** | **0.73 (0.57, 0.84)** | **0.86 (0.69, 0.94)** | **0.76 (0.65, 0.86)** |
| CAG-873 | 0.54 (0.44, 0.63) | 0.18 (0.09, 0.34) | 0.91 (0.78, 0.97) | 0.67 (0.35, 0.88) | 0.54 (0.42, 0.66) | 0.49 (0.36, 0.61) |
| Porphyromonas | 0.61 (0.49, 0.72) | 0.30 (0.17, 0.47) | 0.94 (0.81, 0.98) | 0.83 (0.55, 0.95) | 0.59 (0.46, 0.71) | 0.59 (0.46, 0.71) |
| {Unknown Genus} Prevotellaceae | 0.33 (0.23, 0.42) | 1.00 (0.90, 1.00) | 0.00 (0.00, 0.10) | 0.49 (0.37, 0.60) | NA (NA, NA) | 0.49 (0.36, 0.61) |
| Alloprevotella | 0.57 (0.48, 0.66) | 0.91 (0.76, 0.97) | 0.26 (0.14, 0.42) | 0.54 (0.41, 0.66) | 0.75 (0.47, 0.91) | 0.57 (0.45, 0.69) |
| Paraprevotella | 0.55 (0.42, 0.68) | 0.76 (0.59, 0.87) | 0.40 (0.26, 0.56) | 0.54 (0.40, 0.68) | 0.64 (0.43, 0.80) | 0.57 (0.45, 0.69) |
| Prevotella | 0.50 (0.37, 0.63) | 0.58 (0.41, 0.73) | 0.49 (0.33, 0.64) | 0.51 (0.36, 0.67) | 0.55 (0.38, 0.71) | 0.51 (0.39, 0.64) |
| Prevotella_7 | 0.62 (0.50, 0.74) | 0.79 (0.62, 0.89) | 0.46 (0.30, 0.62) | 0.58 (0.43, 0.71) | 0.70 (0.49, 0.84) | 0.59 (0.46, 0.71) |
| **Prevotella_9** | **0.79 (0.68, 0.90)** | **0.79 (0.62, 0.89)** | **0.77 (0.61, 0.88)** | **0.76 (0.60, 0.88)** | **0.79 (0.63, 0.90)** | **0.66 (0.54, 0.77)** |
| Prevotellaceae Ga6A1 group | 0.50 (0.50, 0.50) | 1.00 (0.90, 1.00) | 0.00 (0.00, 0.10) | 0.49 (0.37, 0.60) | NA (NA, NA) | 0.49 (0.36, 0.61) |
| Prevotellaceae NK3B31 group | 0.57 (0.47, 0.66) | 0.91 (0.76, 0.97) | 0.23 (0.12, 0.39) | 0.53 (0.40, 0.65) | 0.73 (0.43, 0.90) | 0.56 (0.43, 0.68) |
| Prevotellaceae UCG-001 | 0.50 (0.50, 0.50) | 1.00 (0.90, 1.00) | 0.00 (0.00, 0.10) | 0.49 (0.37, 0.60) | NA (NA, NA) | 0.51 (0.39, 0.64) |
| Uncultured-06 | 0.53 (0.44, 0.62) | 0.88 (0.73, 0.95) | 0.20 (0.10, 0.36) | 0.51 (0.38, 0.63) | 0.64 (0.35, 0.85) | 0.51 (0.39, 0.64) |
| Alistipes | 0.40 (0.26, 0.53) | 0.06 (0.02, 0.20) | 0.97 (0.85, 0.99) | 0.67 (0.21, 0.94) | 0.52 (0.40, 0.64) | 0.51 (0.39, 0.64) |
| Rikenellaceae RC9 gut group | 0.48 (0.39, 0.57) | 1.00 (0.90, 1.00) | 0.00 (0.00, 0.10) | 0.49 (0.37, 0.60) | NA (NA, NA) | 0.47 (0.35, 0.60) |
| {Unknown Genus} Tannerellaceae | 0.57 (0.46, 0.68) | 0.33 (0.20, 0.50) | 0.80 (0.64, 0.90) | 0.61 (0.39, 0.80) | 0.56 (0.42, 0.69) | 0.57 (0.45, 0.69) |
| Parabacteroides | 0.54 (0.39, 0.68) | 0.39 (0.25, 0.56) | 0.74 (0.58, 0.86) | 0.59 (0.39, 0.77) | 0.57 (0.42, 0.70) | 0.57 (0.45, 0.69) |
| {Unknown Genus} Uncultured-02 | 0.51 (0.42, 0.60) | 0.18 (0.09, 0.34) | 0.91 (0.78, 0.97) | 0.67 (0.35, 0.88) | 0.54 (0.42, 0.66) | 0.51 (0.39, 0.64) |
| Uncultured-07 | 0.56 (0.48, 0.64) | 1.00 (0.90, 1.00) | 0.14 (0.06, 0.29) | 0.52 (0.40, 0.64) | 1.00 (0.57, 1.00) | 0.54 (0.42, 0.67) |
| Lentimicrobium | 0.50 (0.50, 0.50) | 1.00 (0.90, 1.00) | 0.00 (0.00, 0.10) | 0.49 (0.37, 0.60) | NA (NA, NA) | 0.51 (0.39, 0.64) |
| Sphingobacterium | 0.50 (0.50, 0.50) | 1.00 (0.90, 1.00) | 0.00 (0.00, 0.10) | 0.49 (0.37, 0.60) | NA (NA, NA) | 0.49 (0.36, 0.61) |
| Campylobacter | 0.65 (0.56, 0.74) | 0.36 (0.22, 0.53) | 0.94 (0.81, 0.98) | 0.86 (0.60, 0.96) | 0.61 (0.48, 0.73) | 0.51 (0.39, 0.64) |
| Helicobacter | 0.50 (0.50, 0.50) | 1.00 (0.90, 1.00) | 0.00 (0.00, 0.10) | 0.49 (0.37, 0.60) | NA (NA, NA) | 0.51 (0.39, 0.64) |
| {Unknown Family} Chloroplast | 0.33 (0.21, 0.44) | 1.00 (0.90, 1.00) | 0.00 (0.00, 0.10) | 0.49 (0.37, 0.60) | NA (NA, NA) | 0.49 (0.36, 0.61) |
| {Unknown Family} Gastranaerophilales | 0.68 (0.56, 0.81) | 0.82 (0.66, 0.91) | 0.49 (0.33, 0.64) | 0.60 (0.45, 0.73) | 0.74 (0.54, 0.87) | 0.63 (0.51, 0.75) |
| Bilophila | 0.46 (0.32, 0.60) | 0.33 (0.20, 0.50) | 0.80 (0.64, 0.90) | 0.61 (0.39, 0.80) | 0.56 (0.42, 0.69) | 0.57 (0.45, 0.69) |
| Desulfovibrio | 0.56 (0.43, 0.69) | 0.52 (0.35, 0.67) | 0.66 (0.49, 0.79) | 0.59 (0.41, 0.74) | 0.59 (0.43, 0.73) | 0.51 (0.39, 0.64) |
| Mailhella | 0.54 (0.50, 0.59) | 1.00 (0.90, 1.00) | 0.09 (0.03, 0.22) | 0.51 (0.39, 0.63) | 1.00 (0.44, 1.00) | 0.51 (0.39, 0.64) |
| Uncultured-08 | 0.52 (0.44, 0.59) | 0.12 (0.05, 0.27) | 0.94 (0.81, 0.98) | 0.67 (0.30, 0.90) | 0.53 (0.41, 0.65) | 0.53 (0.40, 0.65) |
| Elusimicrobium | 0.50 (0.50, 0.50) | 1.00 (0.90, 1.00) | 0.00 (0.00, 0.10) | 0.49 (0.37, 0.60) | NA (NA, NA) | 0.49 (0.36, 0.61) |
| {Unknown Class} Firmicutes | 0.34 (0.23, 0.46) | 0.03 (0.01, 0.15) | 0.97 (0.85, 0.99) | 0.50 (0.09, 0.91) | 0.52 (0.40, 0.63) | 0.51 (0.39, 0.64) |
| {Unknown Order} Bacilli | 0.49 (0.46, 0.51) | 1.00 (0.90, 1.00) | 0.00 (0.00, 0.10) | 0.49 (0.37, 0.60) | NA (NA, NA) | 0.50 (0.38, 0.62) |
| Anaeroplasma | 0.51 (0.45, 0.58) | 0.97 (0.85, 0.99) | 0.09 (0.03, 0.22) | 0.50 (0.38, 0.62) | 0.75 (0.30, 0.95) | 0.49 (0.36, 0.61) |
| Bacillus | 0.50 (0.50, 0.50) | 1.00 (0.90, 1.00) | 0.00 (0.00, 0.10) | 0.49 (0.37, 0.60) | NA (NA, NA) | 0.49 (0.36, 0.61) |
| Lysinibacillus | 0.50 (0.50, 0.50) | 1.00 (0.90, 1.00) | 0.00 (0.00, 0.10) | 0.49 (0.37, 0.60) | NA (NA, NA) | 0.51 (0.39, 0.64) |
| Uncultured-09 | 0.47 (0.43, 0.51) | 1.00 (0.90, 1.00) | 0.00 (0.00, 0.10) | 0.49 (0.37, 0.60) | NA (NA, NA) | 0.47 (0.35, 0.60) |
| {Unknown Genus} Erysipelatoclostridiaceae | 0.68 (0.55, 0.81) | 0.58 (0.41, 0.73) | 0.77 (0.61, 0.88) | 0.70 (0.52, 0.84) | 0.66 (0.51, 0.78) | 0.65 (0.52, 0.76) |
| Asteroleplasm | 0.54 (0.48, 0.60) | 0.97 (0.85, 0.99) | 0.11 (0.05, 0.26) | 0.51 (0.39, 0.63) | 0.80 (0.38, 0.96) | 0.53 (0.40, 0.65) |
| Candidatus Stoquefichus | 0.52 (0.49, 0.54) | 0.03 (0.01, 0.15) | 1.00 (0.90, 1.00) | 1.00 (0.21, 1.00) | 0.52 (0.40, 0.64) | 0.51 (0.39, 0.64) |
| Catenibacterium | 0.58 (0.49, 0.67) | 0.91 (0.76, 0.97) | 0.26 (0.14, 0.42) | 0.54 (0.41, 0.66) | 0.75 (0.47, 0.91) | 0.51 (0.39, 0.64) |
| Coprobacillus | 0.60 (0.49, 0.70) | 0.36 (0.22, 0.53) | 0.86 (0.71, 0.94) | 0.71 (0.47, 0.87) | 0.59 (0.45, 0.71) | 0.56 (0.43, 0.68) |
| Erysipelatoclostridium | 0.54 (0.40, 0.68) | 0.39 (0.25, 0.56) | 0.91 (0.78, 0.97) | 0.81 (0.57, 0.93) | 0.62 (0.48, 0.74) | 0.63 (0.51, 0.75) |
| Erysipelotrichaceae UCG-003 | 0.80 (0.69, 0.91) | 0.82 (0.66, 0.91) | 0.69 (0.52, 0.81) | 0.71 (0.55, 0.83) | 0.80 (0.63, 0.90) | 0.75 (0.63, 0.85) |
| UCG-004 | 0.63 (0.55, 0.71) | 0.97 (0.85, 0.99) | 0.29 (0.16, 0.45) | 0.56 (0.43, 0.68) | 0.91 (0.62, 0.98) | 0.51 (0.39, 0.64) |
| {Unknown Genus} Erysipelotrichaceae | 0.50 (0.46, 0.54) | 0.03 (0.01, 0.15) | 1.00 (0.90, 1.00) | 1.00 (0.21, 1.00) | 0.52 (0.40, 0.64) | 0.53 (0.40, 0.65) |
| [Clostridium] innocuum group | 0.65 (0.53, 0.77) | 0.42 (0.27, 0.59) | 0.91 (0.78, 0.97) | 0.82 (0.59, 0.94) | 0.63 (0.49, 0.75) | 0.63 (0.51, 0.75) |
| Allobaculum | 0.50 (0.50, 0.50) | 1.00 (0.90, 1.00) | 0.00 (0.00, 0.10) | 0.49 (0.37, 0.60) | NA (NA, NA) | 0.49 (0.36, 0.61) |
| Dielma | 0.61 (0.51, 0.71) | 0.33 (0.20, 0.50) | 0.89 (0.74, 0.95) | 0.73 (0.48, 0.89) | 0.58 (0.45, 0.71) | 0.60 (0.48, 0.72) |
| Dubosiella | 0.50 (0.50, 0.50) | 1.00 (0.90, 1.00) | 0.00 (0.00, 0.10) | 0.49 (0.37, 0.60) | NA (NA, NA) | 0.51 (0.39, 0.64) |
| Erysipelotrichaceae UCG-006 | 0.50 (0.46, 0.54) | 0.03 (0.01, 0.15) | 1.00 (0.90, 1.00) | 1.00 (0.21, 1.00) | 0.52 (0.40, 0.64) | 0.51 (0.39, 0.64) |
| Erysipelotrichaceae UCG-009 | 0.50 (0.50, 0.50) | 1.00 (0.90, 1.00) | 0.00 (0.00, 0.10) | 0.49 (0.37, 0.60) | NA (NA, NA) | 0.49 (0.36, 0.61) |
| Faecalibaculum | 0.47 (0.43, 0.51) | 1.00 (0.90, 1.00) | 0.00 (0.00, 0.10) | 0.49 (0.37, 0.60) | NA (NA, NA) | 0.49 (0.36, 0.61) |
| Faecalicoccus | 0.50 (0.46, 0.54) | 0.03 (0.01, 0.15) | 0.97 (0.85, 0.99) | 0.50 (0.09, 0.91) | 0.52 (0.40, 0.63) | 0.51 (0.39, 0.64) |
| Faecalitalea | 0.48 (0.37, 0.58) | 0.06 (0.02, 0.20) | 0.97 (0.85, 0.99) | 0.67 (0.21, 0.94) | 0.52 (0.40, 0.64) | 0.53 (0.40, 0.65) |
| Holdemanella | 0.62 (0.51, 0.74) | 0.82 (0.66, 0.91) | 0.43 (0.28, 0.59) | 0.57 (0.43, 0.70) | 0.71 (0.50, 0.86) | 0.51 (0.39, 0.64) |
| Holdemania | 0.54 (0.40, 0.67) | 0.36 (0.22, 0.53) | 0.83 (0.67, 0.92) | 0.67 (0.44, 0.84) | 0.58 (0.44, 0.71) | 0.59 (0.46, 0.71) |
| Merdibacter | 0.60 (0.49, 0.72) | 0.42 (0.27, 0.59) | 0.80 (0.64, 0.90) | 0.67 (0.45, 0.83) | 0.60 (0.45, 0.72) | 0.59 (0.46, 0.71) |
| Solobacterium | 0.57 (0.49, 0.65) | 0.94 (0.80, 0.98) | 0.20 (0.10, 0.36) | 0.53 (0.40, 0.65) | 0.78 (0.45, 0.94) | 0.51 (0.39, 0.64) |
| Turicibacter | 0.49 (0.36, 0.61) | 0.12 (0.05, 0.27) | 0.94 (0.81, 0.98) | 0.67 (0.30, 0.90) | 0.53 (0.41, 0.65) | 0.53 (0.40, 0.65) |
| Uncultured-10 | 0.47 (0.37, 0.57) | 0.06 (0.02, 0.20) | 0.97 (0.85, 0.99) | 0.67 (0.21, 0.94) | 0.52 (0.40, 0.64) | 0.53 (0.40, 0.65) |
| {Unknown Family} Izemoplasmatales | 0.48 (0.37, 0.60) | 0.09 (0.03, 0.24) | 1.00 (0.90, 1.00) | 1.00 (0.44, 1.00) | 0.54 (0.42, 0.65) | 0.51 (0.39, 0.64) |
| {Unknown Family} Lactobacillales | 0.55 (0.44, 0.65) | 0.27 (0.15, 0.44) | 0.86 (0.71, 0.94) | 0.64 (0.39, 0.84) | 0.56 (0.42, 0.68) | 0.57 (0.45, 0.69) |
| Abiotrophia | 0.50 (0.50, 0.50) | 1.00 (0.90, 1.00) | 0.00 (0.00, 0.10) | 0.49 (0.37, 0.60) | NA (NA, NA) | 0.51 (0.39, 0.64) |
| Granulicatella | 0.46 (0.34, 0.58) | 0.15 (0.07, 0.31) | 0.94 (0.81, 0.98) | 0.71 (0.36, 0.92) | 0.54 (0.42, 0.66) | 0.51 (0.39, 0.64) |
| {Unknown Genus} Enterococcaceae | 0.50 (0.50, 0.50) | 1.00 (0.90, 1.00) | 0.00 (0.00, 0.10) | 0.49 (0.37, 0.60) | NA (NA, NA) | 0.51 (0.39, 0.64) |
| **Enterococcus** | **0.76 (0.67, 0.86)** | **0.58 (0.41, 0.73)** | **0.97 (0.85, 0.99)** | **0.95 (0.76, 0.99)** | **0.71 (0.57, 0.82)** | **0.71 (0.58, 0.81)** |
| {Unknown Genus} Lactobacillaceae | 0.46 (0.40, 0.52) | 0.03 (0.01, 0.15) | 1.00 (0.90, 1.00) | 1.00 (0.21, 1.00) | 0.52 (0.40, 0.64) | 0.51 (0.39, 0.64) |
| Amylolactobacillus | 0.50 (0.50, 0.50) | 1.00 (0.90, 1.00) | 0.00 (0.00, 0.10) | 0.49 (0.37, 0.60) | NA (NA, NA) | 0.51 (0.39, 0.64) |
| Fructilactobacillus | 0.50 (0.50, 0.50) | 1.00 (0.90, 1.00) | 0.00 (0.00, 0.10) | 0.49 (0.37, 0.60) | NA (NA, NA) | 0.51 (0.39, 0.64) |
| HT002 | 0.49 (0.46, 0.51) | 1.00 (0.90, 1.00) | 0.00 (0.00, 0.10) | 0.49 (0.37, 0.60) | NA (NA, NA) | 0.49 (0.36, 0.61) |
| Lacticaseibacillus | 0.51 (0.43, 0.59) | 0.09 (0.03, 0.24) | 1.00 (0.90, 1.00) | 1.00 (0.44, 1.00) | 0.54 (0.42, 0.65) | 0.54 (0.42, 0.67) |
| Lactiplantibacillus | 0.50 (0.50, 0.50) | 1.00 (0.90, 1.00) | 0.00 (0.00, 0.10) | 0.49 (0.37, 0.60) | NA (NA, NA) | 0.51 (0.39, 0.64) |
| Lactobacillus | 0.48 (0.35, 0.60) | 0.06 (0.02, 0.20) | 0.97 (0.85, 0.99) | 0.67 (0.21, 0.94) | 0.52 (0.40, 0.64) | 0.51 (0.39, 0.64) |
| Latilactobacillus | 0.52 (0.43, 0.61) | 0.18 (0.09, 0.34) | 0.86 (0.71, 0.94) | 0.55 (0.28, 0.79) | 0.53 (0.40, 0.65) | 0.53 (0.40, 0.65) |
| Leuconostoc | 0.46 (0.36, 0.56) | 1.00 (0.90, 1.00) | 0.06 (0.02, 0.19) | 0.50 (0.38, 0.62) | 1.00 (0.34, 1.00) | 0.51 (0.39, 0.64) |
| Ligilactobacillus | 0.57 (0.50, 0.64) | 0.97 (0.85, 0.99) | 0.17 (0.08, 0.33) | 0.52 (0.40, 0.64) | 0.86 (0.49, 0.97) | 0.51 (0.39, 0.64) |
| Limosilactobacillus | 0.47 (0.36, 0.59) | 0.03 (0.01, 0.15) | 1.00 (0.90, 1.00) | 1.00 (0.21, 1.00) | 0.52 (0.40, 0.64) | 0.51 (0.39, 0.64) |
| Pediococcus | 0.55 (0.50, 0.60) | 0.09 (0.03, 0.24) | 1.00 (0.90, 1.00) | 1.00 (0.44, 1.00) | 0.54 (0.42, 0.65) | 0.51 (0.39, 0.64) |
| Weissella | 0.51 (0.49, 0.54) | 1.00 (0.90, 1.00) | 0.03 (0.01, 0.15) | 0.49 (0.38, 0.61) | 1.00 (0.21, 1.00) | 0.50 (0.38, 0.62) |
| {Unknown Genus} Streptococcaceae | 0.45 (0.31, 0.59) | 0.64 (0.47, 0.78) | 0.49 (0.33, 0.64) | 0.54 (0.39, 0.68) | 0.59 (0.41, 0.74) | 0.54 (0.42, 0.67) |
| Lactococcus | 0.41 (0.29, 0.53) | 0.12 (0.05, 0.27) | 0.89 (0.74, 0.95) | 0.50 (0.22, 0.78) | 0.52 (0.39, 0.64) | 0.50 (0.38, 0.62) |
| Streptococcus | 0.54 (0.40, 0.68) | 0.88 (0.73, 0.95) | 0.31 (0.19, 0.48) | 0.55 (0.41, 0.67) | 0.73 (0.48, 0.89) | 0.56 (0.43, 0.68) |
| {Unknown Genus} Uncultured-03 | 0.53 (0.49, 0.57) | 0.06 (0.02, 0.20) | 1.00 (0.90, 1.00) | 1.00 (0.34, 1.00) | 0.53 (0.41, 0.65) | 0.54 (0.42, 0.67) |
| Paenibacillus | 0.50 (0.50, 0.50) | 1.00 (0.90, 1.00) | 0.00 (0.00, 0.10) | 0.49 (0.37, 0.60) | NA (NA, NA) | 0.51 (0.39, 0.64) |
| **{Unknown Family} RF39** | **0.72 (0.61, 0.84)** | **0.73 (0.56, 0.85)** | **0.71 (0.55, 0.84)** | **0.71 (0.54, 0.83)** | **0.74 (0.57, 0.85)** | **0.71 (0.58, 0.81)** |
| Gemella | 0.49 (0.40, 0.59) | 0.97 (0.85, 0.99) | 0.09 (0.03, 0.22) | 0.50 (0.38, 0.62) | 0.75 (0.30, 0.95) | 0.51 (0.39, 0.64) |
| Staphylococcus | 0.52 (0.47, 0.57) | 0.06 (0.02, 0.20) | 1.00 (0.90, 1.00) | 1.00 (0.34, 1.00) | 0.53 (0.41, 0.65) | 0.53 (0.40, 0.65) |
| {Unknown Order} Clostridia | 0.67 (0.54, 0.80) | 0.42 (0.27, 0.59) | 0.91 (0.78, 0.97) | 0.82 (0.59, 0.94) | 0.63 (0.49, 0.75) | 0.66 (0.54, 0.77) |
| Caldicoprobacter | 0.45 (0.37, 0.53) | 1.00 (0.90, 1.00) | 0.00 (0.00, 0.10) | 0.49 (0.37, 0.60) | NA (NA, NA) | 0.49 (0.36, 0.61) |
| {Unknown Genus} Christensenellaceae | 0.48 (0.42, 0.55) | 1.00 (0.90, 1.00) | 0.00 (0.00, 0.10) | 0.49 (0.37, 0.60) | NA (NA, NA) | 0.49 (0.36, 0.61) |
| Christensenella | 0.49 (0.44, 0.54) | 0.03 (0.01, 0.15) | 1.00 (0.90, 1.00) | 1.00 (0.21, 1.00) | 0.52 (0.40, 0.64) | 0.51 (0.39, 0.64) |
| Christensenellaceae R-7 group | 0.67 (0.54, 0.80) | 0.61 (0.44, 0.75) | 0.71 (0.55, 0.84) | 0.67 (0.49, 0.81) | 0.66 (0.50, 0.79) | 0.63 (0.51, 0.75) |
| **Uncultured-11** | **0.73 (0.61, 0.85)** | **0.91 (0.76, 0.97)** | **0.54 (0.38, 0.70)** | **0.65 (0.51, 0.77)** | **0.86 (0.67, 0.95)** | **0.71 (0.58, 0.81)** |
| {Unknown Family} Clostridia UCG-014 | 0.84 (0.74, 0.94) | 0.73 (0.56, 0.85) | 0.86 (0.71, 0.94) | 0.83 (0.65, 0.92) | 0.77 (0.62, 0.87) | 0.78 (0.66, 0.87) |
| {Unknown Family} Clostridia vadinBB60 group | 0.26 (0.14, 0.38) | 0.03 (0.01, 0.15) | 0.97 (0.85, 0.99) | 0.50 (0.09, 0.91) | 0.52 (0.40, 0.63) | 0.51 (0.39, 0.64) |
| {Unknown Genus} Clostridiaceae | 0.46 (0.38, 0.54) | 1.00 (0.90, 1.00) | 0.00 (0.00, 0.10) | 0.49 (0.37, 0.60) | NA (NA, NA) | 0.47 (0.35, 0.60) |
| Clostridium sensu stricto 1 | 0.33 (0.21, 0.46) | 1.00 (0.90, 1.00) | 0.00 (0.00, 0.10) | 0.49 (0.37, 0.60) | NA (NA, NA) | 0.49 (0.36, 0.61) |
| Sarcina | 0.54 (0.47, 0.61) | 0.97 (0.85, 0.99) | 0.11 (0.05, 0.26) | 0.51 (0.39, 0.63) | 0.80 (0.38, 0.96) | 0.53 (0.40, 0.65) |
| Anaerofustis | 0.52 (0.45, 0.58) | 0.09 (0.03, 0.24) | 0.94 (0.81, 0.98) | 0.60 (0.23, 0.88) | 0.52 (0.40, 0.64) | 0.51 (0.39, 0.64) |
| Eubacterium | 0.50 (0.46, 0.54) | 0.03 (0.01, 0.15) | 0.97 (0.85, 0.99) | 0.50 (0.09, 0.91) | 0.52 (0.40, 0.63) | 0.50 (0.38, 0.62) |
| Defluviitaleaceae UCG-011 | 0.54 (0.46, 0.62) | 0.97 (0.85, 0.99) | 0.14 (0.06, 0.29) | 0.52 (0.39, 0.64) | 0.83 (0.44, 0.97) | 0.54 (0.42, 0.67) |
| {Unknown Genus} Lachnospiraceae | 0.48 (0.34, 0.62) | 0.15 (0.07, 0.31) | 1.00 (0.90, 1.00) | 1.00 (0.57, 1.00) | 0.56 (0.43, 0.67) | 0.59 (0.46, 0.71) |
| [Bacteroides] pectinophilus group | 0.51 (0.49, 0.54) | 1.00 (0.90, 1.00) | 0.03 (0.01, 0.15) | 0.49 (0.38, 0.61) | 1.00 (0.21, 1.00) | 0.50 (0.38, 0.62) |
| [Eubacterium] eligens group | 0.69 (0.57, 0.82) | 0.61 (0.44, 0.75) | 0.74 (0.58, 0.86) | 0.69 (0.51, 0.83) | 0.67 (0.51, 0.79) | 0.68 (0.55, 0.78) |
| [Eubacterium] fissicatena group | 0.60 (0.49, 0.72) | 0.42 (0.27, 0.59) | 0.83 (0.67, 0.92) | 0.70 (0.48, 0.85) | 0.60 (0.46, 0.73) | 0.60 (0.48, 0.72) |
| [Eubacterium] hallii group | 0.58 (0.44, 0.72) | 0.33 (0.20, 0.50) | 0.91 (0.78, 0.97) | 0.79 (0.52, 0.92) | 0.59 (0.46, 0.71) | 0.57 (0.45, 0.69) |
| [Eubacterium] oxidoreducens group | 0.66 (0.52, 0.79) | 0.76 (0.59, 0.87) | 0.57 (0.41, 0.72) | 0.63 (0.47, 0.76) | 0.71 (0.53, 0.85) | 0.65 (0.52, 0.76) |
| [Eubacterium] ruminantium group | 0.64 (0.51, 0.78) | 0.55 (0.38, 0.70) | 0.77 (0.61, 0.88) | 0.69 (0.50, 0.83) | 0.64 (0.49, 0.77) | 0.63 (0.51, 0.75) |
| [Eubacterium] ventriosum group | 0.58 (0.45, 0.72) | 0.85 (0.69, 0.93) | 0.37 (0.23, 0.54) | 0.56 (0.42, 0.69) | 0.72 (0.49, 0.88) | 0.59 (0.46, 0.71) |
| **[Eubacterium] xylanophilum group** | **0.70 (0.58, 0.83)** | **0.55 (0.38, 0.70)** | **0.86 (0.71, 0.94)** | **0.78 (0.58, 0.90)** | **0.67 (0.52, 0.79)** | **0.71 (0.58, 0.81)** |
| [Ruminococcus] gauvreauii group | 0.69 (0.56, 0.82) | 0.88 (0.73, 0.95) | 0.51 (0.36, 0.67) | 0.63 (0.49, 0.75) | 0.82 (0.61, 0.93) | 0.66 (0.54, 0.77) |
| [Ruminococcus] gnavus group | 0.42 (0.29, 0.55) | 0.94 (0.80, 0.98) | 0.14 (0.06, 0.29) | 0.51 (0.39, 0.63) | 0.71 (0.36, 0.92) | 0.50 (0.38, 0.62) |
| [Ruminococcus] torques group | 0.48 (0.33, 0.63) | 0.36 (0.22, 0.53) | 0.91 (0.78, 0.97) | 0.80 (0.55, 0.93) | 0.60 (0.47, 0.72) | 0.63 (0.51, 0.75) |
| Acetitomaculum | 0.49 (0.46, 0.51) | 1.00 (0.90, 1.00) | 0.00 (0.00, 0.10) | 0.49 (0.37, 0.60) | NA (NA, NA) | 0.50 (0.38, 0.62) |
| **Agathobacter** | **0.78 (0.67, 0.89)** | **0.85 (0.69, 0.93)** | **0.63 (0.46, 0.77)** | **0.68 (0.53, 0.80)** | **0.81 (0.63, 0.92)** | **0.69 (0.57, 0.80)** |
| Anaerosporobacter | 0.50 (0.50, 0.50) | 1.00 (0.90, 1.00) | 0.00 (0.00, 0.10) | 0.49 (0.37, 0.60) | NA (NA, NA) | 0.51 (0.39, 0.64) |
| Anaerostignum | 0.52 (0.45, 0.58) | 0.06 (0.02, 0.20) | 1.00 (0.90, 1.00) | 1.00 (0.34, 1.00) | 0.53 (0.41, 0.65) | 0.53 (0.40, 0.65) |
| **Anaerostipes** | **0.80 (0.70, 0.91)** | **0.85 (0.69, 0.93)** | **0.66 (0.49, 0.79)** | **0.70 (0.55, 0.82)** | **0.82 (0.64, 0.92)** | **0.74 (0.61, 0.83)** |
| Blautia | 0.59 (0.45, 0.72) | 0.24 (0.13, 0.41) | 1.00 (0.90, 1.00) | 1.00 (0.68, 1.00) | 0.58 (0.46, 0.70) | 0.60 (0.48, 0.72) |
| Butyrivibrio | 0.63 (0.53, 0.73) | 0.97 (0.85, 0.99) | 0.34 (0.21, 0.51) | 0.58 (0.45, 0.70) | 0.92 (0.67, 0.99) | 0.51 (0.39, 0.64) |
| CAG-56 | 0.66 (0.53, 0.79) | 0.67 (0.50, 0.80) | 0.66 (0.49, 0.79) | 0.65 (0.48, 0.79) | 0.68 (0.51, 0.81) | 0.66 (0.54, 0.77) |
| Catenibacillus | 0.47 (0.41, 0.52) | 1.00 (0.90, 1.00) | 0.00 (0.00, 0.10) | 0.49 (0.37, 0.60) | NA (NA, NA) | 0.49 (0.36, 0.61) |
| Cellulosilyticum | 0.49 (0.46, 0.51) | 1.00 (0.90, 1.00) | 0.00 (0.00, 0.10) | 0.49 (0.37, 0.60) | NA (NA, NA) | 0.49 (0.36, 0.61) |
| Coprococcus | 0.69 (0.57, 0.82) | 0.58 (0.41, 0.73) | 0.71 (0.55, 0.84) | 0.66 (0.47, 0.80) | 0.64 (0.48, 0.77) | 0.65 (0.52, 0.76) |
| Dorea | 0.66 (0.53, 0.80) | 0.58 (0.41, 0.73) | 0.80 (0.64, 0.90) | 0.73 (0.54, 0.86) | 0.67 (0.52, 0.79) | 0.66 (0.54, 0.77) |
| Eisenbergiella | 0.63 (0.51, 0.75) | 0.45 (0.30, 0.62) | 0.89 (0.74, 0.95) | 0.79 (0.57, 0.91) | 0.63 (0.49, 0.75) | 0.66 (0.54, 0.77) |
| Epulopiscium | 0.49 (0.46, 0.51) | 1.00 (0.90, 1.00) | 0.00 (0.00, 0.10) | 0.49 (0.37, 0.60) | NA (NA, NA) | 0.50 (0.38, 0.62) |
| Frisingicoccus | 0.58 (0.46, 0.70) | 0.21 (0.11, 0.38) | 0.97 (0.85, 0.99) | 0.88 (0.53, 0.98) | 0.57 (0.44, 0.68) | 0.59 (0.46, 0.71) |
| Fusicatenibacter | 0.55 (0.41, 0.69) | 0.33 (0.20, 0.50) | 0.86 (0.71, 0.94) | 0.69 (0.44, 0.86) | 0.58 (0.44, 0.70) | 0.59 (0.46, 0.71) |
| GCA-900066575 | 0.64 (0.51, 0.78) | 0.82 (0.66, 0.91) | 0.46 (0.30, 0.62) | 0.59 (0.44, 0.72) | 0.73 (0.52, 0.87) | 0.62 (0.49, 0.73) |
| GCA-900066755 | 0.51 (0.39, 0.63) | 0.42 (0.27, 0.59) | 0.66 (0.49, 0.79) | 0.54 (0.35, 0.71) | 0.55 (0.40, 0.69) | 0.54 (0.42, 0.67) |
| Howardella | 0.51 (0.41, 0.61) | 0.85 (0.69, 0.93) | 0.20 (0.10, 0.36) | 0.50 (0.37, 0.63) | 0.58 (0.32, 0.81) | 0.49 (0.36, 0.61) |
| **Hungatella** | **0.75 (0.64, 0.86)** | **0.67 (0.50, 0.80)** | **0.86 (0.71, 0.94)** | **0.81 (0.63, 0.92)** | **0.73 (0.58, 0.84)** | **0.74 (0.61, 0.83)** |
| Lachnoclostridium | 0.63 (0.49, 0.77) | 0.52 (0.35, 0.67) | 0.80 (0.64, 0.90) | 0.71 (0.51, 0.85) | 0.64 (0.49, 0.76) | 0.66 (0.54, 0.77) |
| Lachnospira | 0.69 (0.57, 0.82) | 0.85 (0.69, 0.93) | 0.46 (0.30, 0.62) | 0.60 (0.45, 0.72) | 0.76 (0.55, 0.89) | 0.63 (0.51, 0.75) |
| Lachnospiraceae FCS020 group | 0.66 (0.53, 0.79) | 0.61 (0.44, 0.75) | 0.69 (0.52, 0.81) | 0.65 (0.47, 0.79) | 0.65 (0.49, 0.78) | 0.63 (0.51, 0.75) |
| Lachnospiraceae FE2018 group | 0.50 (0.50, 0.50) | 1.00 (0.90, 1.00) | 0.00 (0.00, 0.10) | 0.49 (0.37, 0.60) | NA (NA, NA) | 0.51 (0.39, 0.64) |
| Lachnospiraceae NC2004 group | 0.53 (0.38, 0.67) | 0.48 (0.33, 0.65) | 0.74 (0.58, 0.86) | 0.64 (0.45, 0.80) | 0.60 (0.46, 0.74) | 0.62 (0.49, 0.73) |
| Lachnospiraceae ND3007 group | 0.65 (0.52, 0.79) | 0.64 (0.47, 0.78) | 0.69 (0.52, 0.81) | 0.66 (0.48, 0.80) | 0.67 (0.50, 0.80) | 0.65 (0.52, 0.76) |
| Lachnospiraceae NK3A20 group | 0.51 (0.49, 0.54) | 1.00 (0.90, 1.00) | 0.03 (0.01, 0.15) | 0.49 (0.38, 0.61) | 1.00 (0.21, 1.00) | 0.50 (0.38, 0.62) |
| **Lachnospiraceae NK4A136 group** | **0.72 (0.59, 0.84)** | **0.73 (0.56, 0.85)** | **0.71 (0.55, 0.84)** | **0.71 (0.54, 0.83)** | **0.74 (0.57, 0.85)** | **0.72 (0.60, 0.82)** |
| **Lachnospiraceae UCG-001** | **0.79 (0.68, 0.90)** | **0.64 (0.47, 0.78)** | **0.91 (0.78, 0.97)** | **0.88 (0.69, 0.96)** | **0.73 (0.58, 0.84)** | **0.74 (0.61, 0.83)** |
| Lachnospiraceae UCG-003 | 0.59 (0.52, 0.65) | 1.00 (0.90, 1.00) | 0.17 (0.08, 0.33) | 0.53 (0.41, 0.65) | 1.00 (0.61, 1.00) | 0.51 (0.39, 0.64) |
| Lachnospiraceae UCG-004 | 0.50 (0.36, 0.65) | 0.48 (0.33, 0.65) | 0.74 (0.58, 0.86) | 0.64 (0.45, 0.80) | 0.60 (0.46, 0.74) | 0.60 (0.48, 0.72) |
| Lachnospiraceae UCG-006 | 0.43 (0.36, 0.51) | 1.00 (0.90, 1.00) | 0.00 (0.00, 0.10) | 0.49 (0.37, 0.60) | NA (NA, NA) | 0.49 (0.36, 0.61) |
| Lachnospiraceae UCG-008 | 0.32 (0.19, 0.45) | 0.97 (0.85, 0.99) | 0.06 (0.02, 0.19) | 0.49 (0.37, 0.61) | 0.67 (0.21, 0.94) | 0.40 (0.28, 0.52) |
| Lachnospiraceae UCG-009 | 0.50 (0.50, 0.50) | 1.00 (0.90, 1.00) | 0.00 (0.00, 0.10) | 0.49 (0.37, 0.60) | NA (NA, NA) | 0.51 (0.39, 0.64) |
| Lachnospiraceae UCG-010 | 0.47 (0.35, 0.60) | 0.03 (0.01, 0.15) | 1.00 (0.90, 1.00) | 1.00 (0.21, 1.00) | 0.52 (0.40, 0.64) | 0.51 (0.39, 0.64) |
| Lachnotalea | 0.50 (0.50, 0.50) | 1.00 (0.90, 1.00) | 0.00 (0.00, 0.10) | 0.49 (0.37, 0.60) | NA (NA, NA) | 0.51 (0.39, 0.64) |
| Lactonifactor | 0.53 (0.49, 0.57) | 0.06 (0.02, 0.20) | 1.00 (0.90, 1.00) | 1.00 (0.34, 1.00) | 0.53 (0.41, 0.65) | 0.51 (0.39, 0.64) |
| Marvinbryantia | 0.57 (0.43, 0.71) | 0.55 (0.38, 0.70) | 0.71 (0.55, 0.84) | 0.64 (0.46, 0.79) | 0.63 (0.47, 0.76) | 0.53 (0.40, 0.65) |
| Mobilitalea | 0.50 (0.50, 0.50) | 1.00 (0.90, 1.00) | 0.00 (0.00, 0.10) | 0.49 (0.37, 0.60) | NA (NA, NA) | 0.51 (0.39, 0.64) |
| Moryella | 0.52 (0.40, 0.65) | 0.18 (0.09, 0.34) | 0.94 (0.81, 0.98) | 0.75 (0.41, 0.93) | 0.55 (0.42, 0.67) | 0.54 (0.42, 0.67) |
| Oribacterium | 0.49 (0.41, 0.57) | 0.12 (0.05, 0.27) | 0.94 (0.81, 0.98) | 0.67 (0.30, 0.90) | 0.53 (0.41, 0.65) | 0.53 (0.40, 0.65) |
| Roseburia | 0.50 (0.35, 0.64) | 0.42 (0.27, 0.59) | 0.74 (0.58, 0.86) | 0.61 (0.41, 0.78) | 0.58 (0.43, 0.71) | 0.53 (0.40, 0.65) |
| Sellimonas | 0.59 (0.49, 0.69) | 0.30 (0.17, 0.47) | 0.94 (0.81, 0.98) | 0.83 (0.55, 0.95) | 0.59 (0.46, 0.71) | 0.62 (0.49, 0.73) |
| Shuttleworthia | 0.47 (0.42, 0.53) | 1.00 (0.90, 1.00) | 0.00 (0.00, 0.10) | 0.49 (0.37, 0.60) | NA (NA, NA) | 0.49 (0.36, 0.61) |
| Tuzzerella | 0.49 (0.46, 0.51) | 1.00 (0.90, 1.00) | 0.00 (0.00, 0.10) | 0.49 (0.37, 0.60) | NA (NA, NA) | 0.49 (0.36, 0.61) |
| Tyzzerella | 0.58 (0.45, 0.71) | 0.24 (0.13, 0.41) | 0.97 (0.85, 0.99) | 0.89 (0.57, 0.98) | 0.58 (0.45, 0.69) | 0.59 (0.46, 0.71) |
| UC5-1-2E3 | 0.63 (0.52, 0.74) | 0.42 (0.27, 0.59) | 0.86 (0.71, 0.94) | 0.74 (0.51, 0.88) | 0.61 (0.47, 0.74) | 0.65 (0.52, 0.76) |
| Uncultured-12 | 0.44 (0.30, 0.58) | 0.61 (0.44, 0.75) | 0.46 (0.30, 0.62) | 0.51 (0.36, 0.66) | 0.55 (0.38, 0.72) | 0.50 (0.38, 0.62) |
| Monoglobus | 0.48 (0.34, 0.62) | 0.30 (0.17, 0.47) | 0.77 (0.61, 0.88) | 0.56 (0.34, 0.75) | 0.54 (0.40, 0.67) | 0.53 (0.40, 0.65) |
| {Unknown Family} Oscillospirales | 0.57 (0.43, 0.71) | 0.42 (0.27, 0.59) | 0.80 (0.64, 0.90) | 0.67 (0.45, 0.83) | 0.60 (0.45, 0.72) | 0.59 (0.46, 0.71) |
| Hydrogenoanaerobacterium | 0.53 (0.42, 0.65) | 0.30 (0.17, 0.47) | 0.86 (0.71, 0.94) | 0.67 (0.42, 0.85) | 0.57 (0.43, 0.69) | 0.57 (0.45, 0.69) |
| {Unknown Genus} [Clostridium] methylpentosum group | 0.47 (0.35, 0.59) | 0.82 (0.66, 0.91) | 0.20 (0.10, 0.36) | 0.49 (0.36, 0.62) | 0.54 (0.29, 0.77) | 0.49 (0.36, 0.61) |
| {Unknown Genus} [Eubacterium] coprostanoligenes group | 0.53 (0.39, 0.67) | 0.27 (0.15, 0.44) | 0.89 (0.74, 0.95) | 0.69 (0.42, 0.87) | 0.56 (0.43, 0.69) | 0.51 (0.39, 0.64) |
| {Unknown Genus} Butyricicoccaceae | 0.51 (0.49, 0.54) | 1.00 (0.90, 1.00) | 0.03 (0.01, 0.15) | 0.49 (0.38, 0.61) | 1.00 (0.21, 1.00) | 0.50 (0.38, 0.62) |
| **Butyricicoccus** | **0.73 (0.60, 0.85)** | **0.82 (0.66, 0.91)** | **0.63 (0.46, 0.77)** | **0.68 (0.52, 0.80)** | **0.79 (0.60, 0.90)** | **0.71 (0.58, 0.81)** |
| UCG-008 | 0.55 (0.45, 0.64) | 0.85 (0.69, 0.93) | 0.26 (0.14, 0.42) | 0.52 (0.39, 0.65) | 0.64 (0.39, 0.84) | 0.54 (0.42, 0.67) |
| UCG-009 | 0.67 (0.56, 0.79) | 0.76 (0.59, 0.87) | 0.60 (0.44, 0.74) | 0.64 (0.48, 0.77) | 0.72 (0.54, 0.85) | 0.65 (0.52, 0.76) |
| Acetanaerobacterium | 0.50 (0.40, 0.59) | 0.97 (0.85, 0.99) | 0.06 (0.02, 0.19) | 0.49 (0.37, 0.61) | 0.67 (0.21, 0.94) | 0.49 (0.36, 0.61) |
| Fastidiosipila | 0.50 (0.50, 0.50) | 1.00 (0.90, 1.00) | 0.00 (0.00, 0.10) | 0.49 (0.37, 0.60) | NA (NA, NA) | 0.51 (0.39, 0.64) |
| Ruminiclostridium | 0.56 (0.45, 0.66) | 0.27 (0.15, 0.44) | 0.89 (0.74, 0.95) | 0.69 (0.42, 0.87) | 0.56 (0.43, 0.69) | 0.56 (0.43, 0.68) |
| Saccharofermentans | 0.50 (0.50, 0.50) | 1.00 (0.90, 1.00) | 0.00 (0.00, 0.10) | 0.49 (0.37, 0.60) | NA (NA, NA) | 0.51 (0.39, 0.64) |
| Uncultured-13 | 0.53 (0.49, 0.57) | 1.00 (0.90, 1.00) | 0.06 (0.02, 0.19) | 0.50 (0.38, 0.62) | 1.00 (0.34, 1.00) | 0.51 (0.39, 0.64) |
| {Unknown Genus} Oscillospiraceae | 0.40 (0.26, 0.53) | 0.21 (0.11, 0.38) | 0.80 (0.64, 0.90) | 0.50 (0.27, 0.73) | 0.52 (0.39, 0.65) | 0.50 (0.38, 0.62) |
| Colidextribacter | 0.57 (0.42, 0.71) | 0.58 (0.41, 0.73) | 0.69 (0.52, 0.81) | 0.63 (0.46, 0.78) | 0.63 (0.47, 0.77) | 0.56 (0.43, 0.68) |
| Flavonifractor | 0.64 (0.51, 0.78) | 0.55 (0.38, 0.70) | 0.77 (0.61, 0.88) | 0.69 (0.50, 0.83) | 0.64 (0.49, 0.77) | 0.65 (0.52, 0.76) |
| Intestinimonas | 0.75 (0.63, 0.87) | 0.85 (0.69, 0.93) | 0.57 (0.41, 0.72) | 0.65 (0.50, 0.78) | 0.80 (0.61, 0.91) | 0.69 (0.57, 0.80) |
| NK4A214 group | 0.50 (0.36, 0.64) | 0.27 (0.15, 0.44) | 0.86 (0.71, 0.94) | 0.64 (0.39, 0.84) | 0.56 (0.42, 0.68) | 0.57 (0.45, 0.69) |
| Oscillibacter | 0.56 (0.42, 0.70) | 0.67 (0.50, 0.80) | 0.51 (0.36, 0.67) | 0.56 (0.41, 0.71) | 0.62 (0.44, 0.77) | 0.56 (0.43, 0.68) |
| Oscillospira | 0.35 (0.22, 0.47) | 1.00 (0.90, 1.00) | 0.00 (0.00, 0.10) | 0.49 (0.37, 0.60) | NA (NA, NA) | 0.49 (0.36, 0.61) |
| Papillibacter | 0.44 (0.34, 0.53) | 0.06 (0.02, 0.20) | 1.00 (0.90, 1.00) | 1.00 (0.34, 1.00) | 0.53 (0.41, 0.65) | 0.51 (0.39, 0.64) |
| Pseudoflavonifractor | 0.46 (0.37, 0.55) | 1.00 (0.90, 1.00) | 0.00 (0.00, 0.10) | 0.49 (0.37, 0.60) | NA (NA, NA) | 0.49 (0.36, 0.61) |
| UCG-002 | 0.42 (0.28, 0.55) | 0.09 (0.03, 0.24) | 0.94 (0.81, 0.98) | 0.60 (0.23, 0.88) | 0.52 (0.40, 0.64) | 0.50 (0.38, 0.62) |
| UCG-003 | 0.51 (0.37, 0.65) | 0.39 (0.25, 0.56) | 0.74 (0.58, 0.86) | 0.59 (0.39, 0.77) | 0.57 (0.42, 0.70) | 0.57 (0.45, 0.69) |
| UCG-005 | 0.57 (0.43, 0.71) | 0.76 (0.59, 0.87) | 0.46 (0.30, 0.62) | 0.57 (0.42, 0.70) | 0.67 (0.47, 0.82) | 0.59 (0.46, 0.71) |
| UCG-007 | 0.50 (0.50, 0.50) | 1.00 (0.90, 1.00) | 0.00 (0.00, 0.10) | 0.49 (0.37, 0.60) | NA (NA, NA) | 0.51 (0.39, 0.64) |
| Uncultured-14 | 0.55 (0.40, 0.69) | 0.30 (0.17, 0.47) | 0.91 (0.78, 0.97) | 0.77 (0.50, 0.92) | 0.58 (0.45, 0.70) | 0.60 (0.48, 0.72) |
| V9D2013 group | 0.46 (0.39, 0.53) | 1.00 (0.90, 1.00) | 0.00 (0.00, 0.10) | 0.49 (0.37, 0.60) | NA (NA, NA) | 0.49 (0.36, 0.61) |
| {Unknown Genus} Ruminococcaceae | 0.67 (0.54, 0.80) | 0.45 (0.30, 0.62) | 0.89 (0.74, 0.95) | 0.79 (0.57, 0.91) | 0.63 (0.49, 0.75) | 0.68 (0.55, 0.78) |
| [Eubacterium] siraeum group | 0.58 (0.44, 0.72) | 0.55 (0.38, 0.70) | 0.74 (0.58, 0.86) | 0.67 (0.48, 0.81) | 0.63 (0.48, 0.76) | 0.62 (0.49, 0.73) |
| Anaerofilum | 0.49 (0.36, 0.62) | 0.67 (0.50, 0.80) | 0.37 (0.23, 0.54) | 0.50 (0.36, 0.64) | 0.54 (0.35, 0.72) | 0.50 (0.38, 0.62) |
| Anaerotruncus | 0.67 (0.54, 0.79) | 0.61 (0.44, 0.75) | 0.74 (0.58, 0.86) | 0.69 (0.51, 0.83) | 0.67 (0.51, 0.79) | 0.68 (0.55, 0.78) |
| CAG-352 | 0.59 (0.50, 0.69) | 0.91 (0.76, 0.97) | 0.29 (0.16, 0.45) | 0.55 (0.42, 0.67) | 0.77 (0.50, 0.92) | 0.59 (0.46, 0.71) |
| Candidatus Soleaferrea | 0.43 (0.30, 0.56) | 0.94 (0.80, 0.98) | 0.11 (0.05, 0.26) | 0.50 (0.38, 0.62) | 0.67 (0.30, 0.90) | 0.47 (0.35, 0.60) |
| DTU089 | 0.55 (0.42, 0.69) | 0.45 (0.30, 0.62) | 0.74 (0.58, 0.86) | 0.63 (0.43, 0.79) | 0.59 (0.44, 0.72) | 0.59 (0.46, 0.71) |
| Faecalibacterium | 0.64 (0.51, 0.78) | 0.52 (0.35, 0.67) | 0.80 (0.64, 0.90) | 0.71 (0.51, 0.85) | 0.64 (0.49, 0.76) | 0.66 (0.54, 0.77) |
| Fournierella | 0.57 (0.46, 0.69) | 0.88 (0.73, 0.95) | 0.29 (0.16, 0.45) | 0.54 (0.41, 0.66) | 0.71 (0.45, 0.88) | 0.53 (0.40, 0.65) |
| Harryflintia | 0.48 (0.42, 0.55) | 1.00 (0.90, 1.00) | 0.00 (0.00, 0.10) | 0.49 (0.37, 0.60) | NA (NA, NA) | 0.47 (0.35, 0.60) |
| Incertae Sedis | 0.45 (0.31, 0.59) | 0.45 (0.30, 0.62) | 0.66 (0.49, 0.79) | 0.56 (0.37, 0.72) | 0.56 (0.41, 0.70) | 0.54 (0.42, 0.67) |
| Negativibacillus | 0.50 (0.37, 0.64) | 0.15 (0.07, 0.31) | 0.94 (0.81, 0.98) | 0.71 (0.36, 0.92) | 0.54 (0.42, 0.66) | 0.56 (0.43, 0.68) |
| Paludicola | 0.51 (0.46, 0.56) | 1.00 (0.90, 1.00) | 0.03 (0.01, 0.15) | 0.49 (0.38, 0.61) | 1.00 (0.21, 1.00) | 0.50 (0.38, 0.62) |
| Phocea | 0.51 (0.41, 0.62) | 0.82 (0.66, 0.91) | 0.26 (0.14, 0.42) | 0.51 (0.38, 0.64) | 0.60 (0.36, 0.80) | 0.51 (0.39, 0.64) |
| Ruminococcus | 0.62 (0.49, 0.76) | 0.64 (0.47, 0.78) | 0.69 (0.52, 0.81) | 0.66 (0.48, 0.80) | 0.67 (0.50, 0.80) | 0.66 (0.54, 0.77) |
| Subdoligranulum | 0.64 (0.51, 0.77) | 0.36 (0.22, 0.53) | 0.94 (0.81, 0.98) | 0.86 (0.60, 0.96) | 0.61 (0.48, 0.73) | 0.65 (0.52, 0.76) |
| UBA1819 | 0.63 (0.49, 0.77) | 0.52 (0.35, 0.67) | 0.77 (0.61, 0.88) | 0.68 (0.48, 0.83) | 0.63 (0.48, 0.76) | 0.65 (0.52, 0.76) |
| Uncultured-15 | 0.68 (0.55, 0.80) | 0.42 (0.27, 0.59) | 0.89 (0.74, 0.95) | 0.78 (0.55, 0.91) | 0.62 (0.48, 0.74) | 0.66 (0.54, 0.77) |
| {Unknown Genus} UCG-010 | 0.31 (0.18, 0.44) | 1.00 (0.90, 1.00) | 0.00 (0.00, 0.10) | 0.49 (0.37, 0.60) | NA (NA, NA) | 0.35 (0.24, 0.48) |
| {Unknown Genus} UCG-011 | 0.54 (0.47, 0.61) | 0.94 (0.80, 0.98) | 0.14 (0.06, 0.29) | 0.51 (0.39, 0.63) | 0.71 (0.36, 0.92) | 0.51 (0.39, 0.64) |
| {Unknown Genus} Uncultured-04 | 0.53 (0.42, 0.64) | 0.33 (0.20, 0.50) | 0.80 (0.64, 0.90) | 0.61 (0.39, 0.80) | 0.56 (0.42, 0.69) | 0.56 (0.43, 0.68) |
| Peptococcus | 0.44 (0.32, 0.57) | 0.03 (0.01, 0.15) | 1.00 (0.90, 1.00) | 1.00 (0.21, 1.00) | 0.52 (0.40, 0.64) | 0.53 (0.40, 0.65) |
| Uncultured-16 | 0.56 (0.42, 0.70) | 0.79 (0.62, 0.89) | 0.37 (0.23, 0.54) | 0.54 (0.40, 0.67) | 0.65 (0.43, 0.82) | 0.56 (0.43, 0.68) |
| [Eubacterium] brachy group | 0.55 (0.48, 0.62) | 0.15 (0.07, 0.31) | 0.97 (0.85, 0.99) | 0.83 (0.44, 0.97) | 0.55 (0.43, 0.67) | 0.57 (0.45, 0.69) |
| [Eubacterium] nodatum group | 0.45 (0.32, 0.58) | 0.21 (0.11, 0.38) | 0.89 (0.74, 0.95) | 0.64 (0.35, 0.85) | 0.54 (0.42, 0.67) | 0.54 (0.42, 0.67) |
| [Eubacterium] saphenum group | 0.50 (0.46, 0.54) | 1.00 (0.90, 1.00) | 0.03 (0.01, 0.15) | 0.49 (0.38, 0.61) | 1.00 (0.21, 1.00) | 0.49 (0.36, 0.61) |
| Anaerovorax | 0.52 (0.49, 0.54) | 0.03 (0.01, 0.15) | 1.00 (0.90, 1.00) | 1.00 (0.21, 1.00) | 0.52 (0.40, 0.64) | 0.51 (0.39, 0.64) |
| Family XIII AD3011 group | 0.40 (0.26, 0.54) | 0.18 (0.09, 0.34) | 0.91 (0.78, 0.97) | 0.67 (0.35, 0.88) | 0.54 (0.42, 0.66) | 0.53 (0.40, 0.65) |
| Family XIII UCG-001 | 0.66 (0.53, 0.80) | 0.76 (0.59, 0.87) | 0.60 (0.44, 0.74) | 0.64 (0.48, 0.77) | 0.72 (0.54, 0.85) | 0.65 (0.52, 0.76) |
| Mogibacterium | 0.53 (0.47, 0.59) | 0.09 (0.03, 0.24) | 0.97 (0.85, 0.99) | 0.75 (0.30, 0.95) | 0.53 (0.41, 0.65) | 0.54 (0.42, 0.67) |
| Uncultured-17 | 0.50 (0.50, 0.50) | 1.00 (0.90, 1.00) | 0.00 (0.00, 0.10) | 0.49 (0.37, 0.60) | NA (NA, NA) | 0.51 (0.39, 0.64) |
| Anaerococcus | 0.55 (0.48, 0.61) | 0.12 (0.05, 0.27) | 0.97 (0.85, 0.99) | 0.80 (0.38, 0.96) | 0.54 (0.42, 0.66) | 0.51 (0.39, 0.64) |
| Ezakiella | 0.50 (0.43, 0.56) | 1.00 (0.90, 1.00) | 0.00 (0.00, 0.10) | 0.49 (0.37, 0.60) | NA (NA, NA) | 0.47 (0.35, 0.60) |
| Fenollaria | 0.58 (0.49, 0.67) | 0.24 (0.13, 0.41) | 0.94 (0.81, 0.98) | 0.80 (0.49, 0.94) | 0.57 (0.44, 0.69) | 0.60 (0.48, 0.72) |
| Finegoldia | 0.59 (0.52, 0.67) | 0.21 (0.11, 0.38) | 1.00 (0.90, 1.00) | 1.00 (0.65, 1.00) | 0.57 (0.45, 0.69) | 0.60 (0.48, 0.72) |
| Helcococcus | 0.50 (0.50, 0.50) | 1.00 (0.90, 1.00) | 0.00 (0.00, 0.10) | 0.49 (0.37, 0.60) | NA (NA, NA) | 0.51 (0.39, 0.64) |
| Murdochiella | 0.49 (0.46, 0.51) | 1.00 (0.90, 1.00) | 0.00 (0.00, 0.10) | 0.49 (0.37, 0.60) | NA (NA, NA) | 0.49 (0.36, 0.61) |
| Parvimonas | 0.47 (0.41, 0.53) | 1.00 (0.90, 1.00) | 0.00 (0.00, 0.10) | 0.49 (0.37, 0.60) | NA (NA, NA) | 0.49 (0.36, 0.61) |
| Peptoniphilus | 0.42 (0.35, 0.50) | 1.00 (0.90, 1.00) | 0.00 (0.00, 0.10) | 0.49 (0.37, 0.60) | NA (NA, NA) | 0.49 (0.36, 0.61) |
| {Unknown Genus} Peptostreptococcaceae | 0.50 (0.50, 0.50) | 1.00 (0.90, 1.00) | 0.00 (0.00, 0.10) | 0.49 (0.37, 0.60) | NA (NA, NA) | 0.49 (0.36, 0.61) |
| Clostridioides | 0.50 (0.50, 0.50) | 1.00 (0.90, 1.00) | 0.00 (0.00, 0.10) | 0.49 (0.37, 0.60) | NA (NA, NA) | 0.49 (0.36, 0.61) |
| Criibacterium | 0.50 (0.50, 0.50) | 1.00 (0.90, 1.00) | 0.00 (0.00, 0.10) | 0.49 (0.37, 0.60) | NA (NA, NA) | 0.51 (0.39, 0.64) |
| Intestinibacter | 0.57 (0.43, 0.71) | 0.79 (0.62, 0.89) | 0.46 (0.30, 0.62) | 0.58 (0.43, 0.71) | 0.70 (0.49, 0.84) | 0.59 (0.46, 0.71) |
| Paeniclostridium | 0.50 (0.50, 0.50) | 1.00 (0.90, 1.00) | 0.00 (0.00, 0.10) | 0.49 (0.37, 0.60) | NA (NA, NA) | 0.49 (0.36, 0.61) |
| Peptostreptococcus | 0.50 (0.50, 0.50) | 1.00 (0.90, 1.00) | 0.00 (0.00, 0.10) | 0.49 (0.37, 0.60) | NA (NA, NA) | 0.51 (0.39, 0.64) |
| Romboutsia | 0.70 (0.58, 0.83) | 0.88 (0.73, 0.95) | 0.54 (0.38, 0.70) | 0.64 (0.50, 0.77) | 0.83 (0.63, 0.93) | 0.71 (0.58, 0.81) |
| Terrisporobacter | 0.62 (0.51, 0.73) | 0.94 (0.80, 0.98) | 0.31 (0.19, 0.48) | 0.56 (0.43, 0.69) | 0.85 (0.58, 0.96) | 0.62 (0.49, 0.73) |
| Uncultured-18 | 0.51 (0.47, 0.56) | 1.00 (0.90, 1.00) | 0.06 (0.02, 0.19) | 0.50 (0.38, 0.62) | 1.00 (0.34, 1.00) | 0.50 (0.38, 0.62) |
| {Unknown Family} Uncultured | 0.54 (0.45, 0.62) | 0.88 (0.73, 0.95) | 0.20 (0.10, 0.36) | 0.51 (0.38, 0.63) | 0.64 (0.35, 0.85) | 0.53 (0.40, 0.65) |
| Desulfurispora | 0.50 (0.50, 0.50) | 1.00 (0.90, 1.00) | 0.00 (0.00, 0.10) | 0.49 (0.37, 0.60) | NA (NA, NA) | 0.51 (0.39, 0.64) |
| {Unknown Family} DTU014 | 0.52 (0.42, 0.62) | 0.09 (0.03, 0.24) | 0.97 (0.85, 0.99) | 0.75 (0.30, 0.95) | 0.53 (0.41, 0.65) | 0.54 (0.42, 0.67) |
| {Unknown Family} MBA03 | 0.42 (0.33, 0.51) | 0.06 (0.02, 0.20) | 1.00 (0.90, 1.00) | 1.00 (0.34, 1.00) | 0.53 (0.41, 0.65) | 0.53 (0.40, 0.65) |
| {Unknown Order} Negativicutes | 0.58 (0.47, 0.68) | 0.33 (0.20, 0.50) | 0.83 (0.67, 0.92) | 0.65 (0.41, 0.83) | 0.57 (0.43, 0.69) | 0.59 (0.46, 0.71) |
| {Unknown Genus} Acidaminococcaceae | 0.50 (0.50, 0.50) | 1.00 (0.90, 1.00) | 0.00 (0.00, 0.10) | 0.49 (0.37, 0.60) | NA (NA, NA) | 0.51 (0.39, 0.64) |
| Acidaminococcus | 0.50 (0.39, 0.60) | 0.09 (0.03, 0.24) | 0.97 (0.85, 0.99) | 0.75 (0.30, 0.95) | 0.53 (0.41, 0.65) | 0.54 (0.42, 0.67) |
| Phascolarctobacterium | 0.63 (0.50, 0.77) | 0.61 (0.44, 0.75) | 0.63 (0.46, 0.77) | 0.61 (0.44, 0.75) | 0.63 (0.46, 0.77) | 0.60 (0.48, 0.72) |
| Succiniclasticum | 0.52 (0.46, 0.58) | 0.09 (0.03, 0.24) | 1.00 (0.90, 1.00) | 1.00 (0.44, 1.00) | 0.54 (0.42, 0.65) | 0.51 (0.39, 0.64) |
| Megamonas | 0.47 (0.43, 0.51) | 1.00 (0.90, 1.00) | 0.00 (0.00, 0.10) | 0.49 (0.37, 0.60) | NA (NA, NA) | 0.49 (0.36, 0.61) |
| Mitsuokella | 0.48 (0.46, 0.51) | 1.00 (0.90, 1.00) | 0.00 (0.00, 0.10) | 0.49 (0.37, 0.60) | NA (NA, NA) | 0.49 (0.36, 0.61) |
| Selenomonas | 0.50 (0.50, 0.50) | 1.00 (0.90, 1.00) | 0.00 (0.00, 0.10) | 0.49 (0.37, 0.60) | NA (NA, NA) | 0.51 (0.39, 0.64) |
| Uncultured-19 | 0.56 (0.49, 0.62) | 0.97 (0.85, 0.99) | 0.14 (0.06, 0.29) | 0.52 (0.39, 0.64) | 0.83 (0.44, 0.97) | 0.51 (0.39, 0.64) |
| {Unknown Genus} Veillonellaceae | 0.45 (0.39, 0.52) | 1.00 (0.90, 1.00) | 0.00 (0.00, 0.10) | 0.49 (0.37, 0.60) | NA (NA, NA) | 0.47 (0.35, 0.60) |
| Allisonella | 0.40 (0.32, 0.49) | 0.03 (0.01, 0.15) | 0.97 (0.85, 0.99) | 0.50 (0.09, 0.91) | 0.52 (0.40, 0.63) | 0.50 (0.38, 0.62) |
| Dialister | 0.50 (0.36, 0.64) | 0.88 (0.73, 0.95) | 0.26 (0.14, 0.42) | 0.53 (0.40, 0.65) | 0.69 (0.42, 0.87) | 0.56 (0.43, 0.68) |
| Megasphaera | 0.42 (0.35, 0.49) | 1.00 (0.90, 1.00) | 0.00 (0.00, 0.10) | 0.49 (0.37, 0.60) | NA (NA, NA) | 0.49 (0.36, 0.61) |
| Uncultured-20 | 0.50 (0.50, 0.50) | 1.00 (0.90, 1.00) | 0.00 (0.00, 0.10) | 0.49 (0.37, 0.60) | NA (NA, NA) | 0.51 (0.39, 0.64) |
| Veillonella | 0.69 (0.56, 0.82) | 0.73 (0.56, 0.85) | 0.69 (0.52, 0.81) | 0.69 (0.52, 0.81) | 0.73 (0.56, 0.85) | 0.60 (0.48, 0.72) |
| Syntrophomonas | 0.50 (0.50, 0.50) | 1.00 (0.90, 1.00) | 0.00 (0.00, 0.10) | 0.49 (0.37, 0.60) | NA (NA, NA) | 0.51 (0.39, 0.64) |
| {Unknown Order} Uncultured | 0.47 (0.41, 0.54) | 0.03 (0.01, 0.15) | 1.00 (0.90, 1.00) | 1.00 (0.21, 1.00) | 0.52 (0.40, 0.64) | 0.51 (0.39, 0.64) |
| Cetobacterium | 0.50 (0.50, 0.50) | 1.00 (0.90, 1.00) | 0.00 (0.00, 0.10) | 0.49 (0.37, 0.60) | NA (NA, NA) | 0.51 (0.39, 0.64) |
| Fusobacterium | 0.56 (0.44, 0.68) | 0.27 (0.15, 0.44) | 0.94 (0.81, 0.98) | 0.82 (0.52, 0.95) | 0.58 (0.45, 0.70) | 0.59 (0.46, 0.71) |
| Leptotrichia | 0.52 (0.49, 0.54) | 0.03 (0.01, 0.15) | 1.00 (0.90, 1.00) | 1.00 (0.21, 1.00) | 0.52 (0.40, 0.64) | 0.51 (0.39, 0.64) |
| Oceanivirga | 0.50 (0.50, 0.50) | 1.00 (0.90, 1.00) | 0.00 (0.00, 0.10) | 0.49 (0.37, 0.60) | NA (NA, NA) | 0.49 (0.36, 0.61) |
| Sneathia | 0.48 (0.46, 0.51) | 1.00 (0.90, 1.00) | 0.00 (0.00, 0.10) | 0.49 (0.37, 0.60) | NA (NA, NA) | 0.49 (0.36, 0.61) |
| {Unknown Genus} WD2101 soil group | 0.50 (0.50, 0.50) | 1.00 (0.90, 1.00) | 0.00 (0.00, 0.10) | 0.49 (0.37, 0.60) | NA (NA, NA) | 0.51 (0.39, 0.64) |
| {Unknown Class} Proteobacteria | 0.50 (0.50, 0.50) | 1.00 (0.90, 1.00) | 0.00 (0.00, 0.10) | 0.49 (0.37, 0.60) | NA (NA, NA) | 0.49 (0.36, 0.61) |
| {Unknown Genus} Uncultured-05 | 0.31 (0.18, 0.44) | 0.18 (0.09, 0.34) | 0.86 (0.71, 0.94) | 0.55 (0.28, 0.79) | 0.53 (0.40, 0.65) | 0.53 (0.40, 0.65) |
| {Unknown Genus} Mitochondria | 0.50 (0.50, 0.50) | 1.00 (0.90, 1.00) | 0.00 (0.00, 0.10) | 0.49 (0.37, 0.60) | NA (NA, NA) | 0.49 (0.36, 0.61) |
| {Unknown Order} Gammaproteobacteria | 0.68 (0.56, 0.81) | 0.61 (0.44, 0.75) | 0.86 (0.71, 0.94) | 0.80 (0.61, 0.91) | 0.70 (0.55, 0.81) | 0.69 (0.57, 0.80) |
| Advenella | 0.52 (0.49, 0.54) | 0.03 (0.01, 0.15) | 1.00 (0.90, 1.00) | 1.00 (0.21, 1.00) | 0.52 (0.40, 0.64) | 0.51 (0.39, 0.64) |
| Alcaligenes | 0.52 (0.49, 0.54) | 0.03 (0.01, 0.15) | 1.00 (0.90, 1.00) | 1.00 (0.21, 1.00) | 0.52 (0.40, 0.64) | 0.53 (0.40, 0.65) |
| Lautropia | 0.50 (0.50, 0.50) | 1.00 (0.90, 1.00) | 0.00 (0.00, 0.10) | 0.49 (0.37, 0.60) | NA (NA, NA) | 0.51 (0.39, 0.64) |
| {Unknown Genus} Comamonadaceae | 0.50 (0.50, 0.50) | 1.00 (0.90, 1.00) | 0.00 (0.00, 0.10) | 0.49 (0.37, 0.60) | NA (NA, NA) | 0.51 (0.39, 0.64) |
| Comamonas | 0.66 (0.56, 0.75) | 0.39 (0.25, 0.56) | 0.94 (0.81, 0.98) | 0.87 (0.62, 0.96) | 0.62 (0.49, 0.74) | 0.68 (0.55, 0.78) |
| Neisseria | 0.50 (0.46, 0.54) | 0.03 (0.01, 0.15) | 0.97 (0.85, 0.99) | 0.50 (0.09, 0.91) | 0.52 (0.40, 0.63) | 0.51 (0.39, 0.64) |
| Uncultured-21 | 0.49 (0.46, 0.51) | 1.00 (0.90, 1.00) | 0.00 (0.00, 0.10) | 0.49 (0.37, 0.60) | NA (NA, NA) | 0.49 (0.36, 0.61) |
| Oxalobacter | 0.59 (0.48, 0.70) | 0.94 (0.80, 0.98) | 0.26 (0.14, 0.42) | 0.54 (0.42, 0.67) | 0.82 (0.52, 0.95) | 0.57 (0.45, 0.69) |
| Uncultured-22 | 0.50 (0.50, 0.50) | 1.00 (0.90, 1.00) | 0.00 (0.00, 0.10) | 0.49 (0.37, 0.60) | NA (NA, NA) | 0.49 (0.36, 0.61) |
| Parasutterella | 0.64 (0.50, 0.77) | 0.45 (0.30, 0.62) | 0.83 (0.67, 0.92) | 0.71 (0.50, 0.86) | 0.62 (0.47, 0.74) | 0.60 (0.48, 0.72) |
| Sutterella | 0.50 (0.36, 0.64) | 0.64 (0.47, 0.78) | 0.46 (0.30, 0.62) | 0.53 (0.37, 0.67) | 0.57 (0.39, 0.73) | 0.53 (0.40, 0.65) |
| {Unknown Family} Enterobacterales | 0.63 (0.53, 0.74) | 0.42 (0.27, 0.59) | 0.86 (0.71, 0.94) | 0.74 (0.51, 0.88) | 0.61 (0.47, 0.74) | 0.63 (0.51, 0.75) |
| Aestuariibacter | 0.50 (0.50, 0.50) | 1.00 (0.90, 1.00) | 0.00 (0.00, 0.10) | 0.49 (0.37, 0.60) | NA (NA, NA) | 0.51 (0.39, 0.64) |
| {Unknown Genus} Enterobacteriaceae | 0.68 (0.56, 0.81) | 0.52 (0.35, 0.67) | 0.89 (0.74, 0.95) | 0.81 (0.60, 0.92) | 0.66 (0.52, 0.78) | 0.69 (0.57, 0.80) |
| Buttiauxella | 0.52 (0.49, 0.54) | 0.03 (0.01, 0.15) | 1.00 (0.90, 1.00) | 1.00 (0.21, 1.00) | 0.52 (0.40, 0.64) | 0.53 (0.40, 0.65) |
| Cedecea | 0.50 (0.50, 0.50) | 1.00 (0.90, 1.00) | 0.00 (0.00, 0.10) | 0.49 (0.37, 0.60) | NA (NA, NA) | 0.51 (0.39, 0.64) |
| **Citrobacter** | **0.72 (0.61, 0.83)** | **0.58 (0.41, 0.73)** | **0.89 (0.74, 0.95)** | **0.83 (0.63, 0.93)** | **0.69 (0.54, 0.80)** | **0.49 (0.36, 0.61)** |
| Cronobacter | 0.48 (0.46, 0.51) | 1.00 (0.90, 1.00) | 0.00 (0.00, 0.10) | 0.49 (0.37, 0.60) | NA (NA, NA) | 0.49 (0.36, 0.61) |
| **Enterobacter** | **0.74 (0.64, 0.85)** | **0.61 (0.44, 0.75)** | **0.89 (0.74, 0.95)** | **0.83 (0.64, 0.93)** | **0.70 (0.56, 0.82)** | **0.75 (0.63, 0.85)** |
| Escherichia-Shigella | 0.65 (0.52, 0.78) | 0.42 (0.27, 0.59) | 0.91 (0.78, 0.97) | 0.82 (0.59, 0.94) | 0.63 (0.49, 0.75) | 0.65 (0.52, 0.76) |
| **Klebsiella** | **0.71 (0.60, 0.82)** | **0.55 (0.38, 0.70)** | **0.89 (0.74, 0.95)** | **0.82 (0.61, 0.93)** | **0.67 (0.53, 0.79)** | **0.49 (0.36, 0.61)** |
| Kluyvera | 0.62 (0.53, 0.71) | 0.30 (0.17, 0.47) | 0.94 (0.81, 0.98) | 0.83 (0.55, 0.95) | 0.59 (0.46, 0.71) | 0.63 (0.51, 0.75) |
| Kosakonia | 0.50 (0.50, 0.50) | 1.00 (0.90, 1.00) | 0.00 (0.00, 0.10) | 0.49 (0.37, 0.60) | NA (NA, NA) | 0.49 (0.36, 0.61) |
| Lelliottia | 0.50 (0.44, 0.56) | 0.06 (0.02, 0.20) | 0.94 (0.81, 0.98) | 0.50 (0.15, 0.85) | 0.52 (0.40, 0.63) | 0.51 (0.39, 0.64) |
| Pluralibacter | 0.50 (0.50, 0.50) | 1.00 (0.90, 1.00) | 0.00 (0.00, 0.10) | 0.49 (0.37, 0.60) | NA (NA, NA) | 0.49 (0.36, 0.61) |
| Raoultella | 0.56 (0.48, 0.64) | 0.18 (0.09, 0.34) | 0.94 (0.81, 0.98) | 0.75 (0.41, 0.93) | 0.55 (0.42, 0.67) | 0.56 (0.43, 0.68) |
| Salmonella | 0.50 (0.45, 0.56) | 0.06 (0.02, 0.20) | 0.97 (0.85, 0.99) | 0.67 (0.21, 0.94) | 0.52 (0.40, 0.64) | 0.53 (0.40, 0.65) |
| Erwinia | 0.59 (0.52, 0.67) | 0.21 (0.11, 0.38) | 0.97 (0.85, 0.99) | 0.88 (0.53, 0.98) | 0.57 (0.44, 0.68) | 0.56 (0.43, 0.68) |
| Pantoea | 0.52 (0.47, 0.57) | 0.06 (0.02, 0.20) | 1.00 (0.90, 1.00) | 1.00 (0.34, 1.00) | 0.53 (0.41, 0.65) | 0.53 (0.40, 0.65) |
| Siccibacter | 0.53 (0.46, 0.60) | 0.12 (0.05, 0.27) | 0.94 (0.81, 0.98) | 0.67 (0.30, 0.90) | 0.53 (0.41, 0.65) | 0.54 (0.42, 0.67) |
| Tatumella | 0.56 (0.50, 0.62) | 0.12 (0.05, 0.27) | 1.00 (0.90, 1.00) | 1.00 (0.51, 1.00) | 0.55 (0.43, 0.66) | 0.57 (0.45, 0.69) |
| Hafnia-Obesumbacterium | 0.52 (0.45, 0.58) | 0.06 (0.02, 0.20) | 1.00 (0.90, 1.00) | 1.00 (0.34, 1.00) | 0.53 (0.41, 0.65) | 0.54 (0.42, 0.67) |
| Morganella | 0.50 (0.50, 0.50) | 1.00 (0.90, 1.00) | 0.00 (0.00, 0.10) | 0.49 (0.37, 0.60) | NA (NA, NA) | 0.51 (0.39, 0.64) |
| Proteus | 0.50 (0.50, 0.50) | 1.00 (0.90, 1.00) | 0.00 (0.00, 0.10) | 0.49 (0.37, 0.60) | NA (NA, NA) | 0.49 (0.36, 0.61) |
| Providencia | 0.48 (0.46, 0.51) | 1.00 (0.90, 1.00) | 0.00 (0.00, 0.10) | 0.49 (0.37, 0.60) | NA (NA, NA) | 0.49 (0.36, 0.61) |
| {Unknown Genus} Pasteurellaceae | 0.42 (0.29, 0.54) | 0.06 (0.02, 0.20) | 0.94 (0.81, 0.98) | 0.50 (0.15, 0.85) | 0.52 (0.40, 0.63) | 0.50 (0.38, 0.62) |
| Actinobacillus | 0.50 (0.50, 0.50) | 1.00 (0.90, 1.00) | 0.00 (0.00, 0.10) | 0.49 (0.37, 0.60) | NA (NA, NA) | 0.51 (0.39, 0.64) |
| Aggregatibacter | 0.50 (0.46, 0.54) | 1.00 (0.90, 1.00) | 0.00 (0.00, 0.10) | 0.49 (0.37, 0.60) | NA (NA, NA) | 0.49 (0.36, 0.61) |
| Haemophilus | 0.40 (0.28, 0.51) | 1.00 (0.90, 1.00) | 0.00 (0.00, 0.10) | 0.49 (0.37, 0.60) | NA (NA, NA) | 0.49 (0.36, 0.61) |
| Pasteurella | 0.50 (0.50, 0.50) | 1.00 (0.90, 1.00) | 0.00 (0.00, 0.10) | 0.49 (0.37, 0.60) | NA (NA, NA) | 0.51 (0.39, 0.64) |
| {Unknown Genus} Succinivibrionaceae | 0.47 (0.43, 0.51) | 1.00 (0.90, 1.00) | 0.00 (0.00, 0.10) | 0.49 (0.37, 0.60) | NA (NA, NA) | 0.49 (0.36, 0.61) |
| Succinatimonas | 0.50 (0.50, 0.50) | 1.00 (0.90, 1.00) | 0.00 (0.00, 0.10) | 0.49 (0.37, 0.60) | NA (NA, NA) | 0.51 (0.39, 0.64) |
| Succinivibrio | 0.44 (0.37, 0.52) | 1.00 (0.90, 1.00) | 0.00 (0.00, 0.10) | 0.49 (0.37, 0.60) | NA (NA, NA) | 0.47 (0.35, 0.60) |
| Vibrio | 0.57 (0.50, 0.65) | 0.18 (0.09, 0.34) | 0.97 (0.85, 0.99) | 0.86 (0.49, 0.97) | 0.56 (0.43, 0.67) | 0.59 (0.46, 0.71) |
| Rahnella1 | 0.52 (0.49, 0.54) | 0.03 (0.01, 0.15) | 1.00 (0.90, 1.00) | 1.00 (0.21, 1.00) | 0.52 (0.40, 0.64) | 0.51 (0.39, 0.64) |
| Serratia | 0.59 (0.52, 0.67) | 0.21 (0.11, 0.38) | 0.97 (0.85, 0.99) | 0.88 (0.53, 0.98) | 0.57 (0.44, 0.68) | 0.60 (0.48, 0.72) |
| Acinetobacter | 0.50 (0.50, 0.50) | 1.00 (0.90, 1.00) | 0.00 (0.00, 0.10) | 0.49 (0.37, 0.60) | NA (NA, NA) | 0.49 (0.36, 0.61) |
| Uncultured-23 | 0.50 (0.50, 0.50) | 1.00 (0.90, 1.00) | 0.00 (0.00, 0.10) | 0.49 (0.37, 0.60) | NA (NA, NA) | 0.51 (0.39, 0.64) |
| Pseudomonas | 0.48 (0.41, 0.56) | 1.00 (0.90, 1.00) | 0.00 (0.00, 0.10) | 0.49 (0.37, 0.60) | NA (NA, NA) | 0.49 (0.36, 0.61) |
| {Unknown Genus} Xanthomonadaceae | 0.50 (0.50, 0.50) | 1.00 (0.90, 1.00) | 0.00 (0.00, 0.10) | 0.49 (0.37, 0.60) | NA (NA, NA) | 0.49 (0.36, 0.61) |
| Stenotrophomonas | 0.50 (0.50, 0.50) | 1.00 (0.90, 1.00) | 0.00 (0.00, 0.10) | 0.49 (0.37, 0.60) | NA (NA, NA) | 0.49 (0.36, 0.61) |
| Cloacibacillus | 0.58 (0.50, 0.66) | 0.21 (0.11, 0.38) | 0.97 (0.85, 0.99) | 0.88 (0.53, 0.98) | 0.57 (0.44, 0.68) | 0.57 (0.45, 0.69) |
| Jonquetella | 0.50 (0.50, 0.50) | 1.00 (0.90, 1.00) | 0.00 (0.00, 0.10) | 0.49 (0.37, 0.60) | NA (NA, NA) | 0.51 (0.39, 0.64) |
| Pyramidobacter | 0.50 (0.50, 0.50) | 1.00 (0.90, 1.00) | 0.00 (0.00, 0.10) | 0.49 (0.37, 0.60) | NA (NA, NA) | 0.49 (0.36, 0.61) |
| Synergistes | 0.52 (0.49, 0.54) | 0.03 (0.01, 0.15) | 1.00 (0.90, 1.00) | 1.00 (0.21, 1.00) | 0.52 (0.40, 0.64) | 0.51 (0.39, 0.64) |
| {Unknown Family} WCHB1-41 | 0.50 (0.50, 0.50) | 1.00 (0.90, 1.00) | 0.00 (0.00, 0.10) | 0.49 (0.37, 0.60) | NA (NA, NA) | 0.51 (0.39, 0.64) |
| {Unknown Genus} VadinBE97 | 0.45 (0.34, 0.56) | 0.12 (0.05, 0.27) | 0.89 (0.74, 0.95) | 0.50 (0.22, 0.78) | 0.52 (0.39, 0.64) | 0.51 (0.39, 0.64) |
| {Unknown Genus} Victivallaceae | 0.45 (0.37, 0.52) | 1.00 (0.90, 1.00) | 0.00 (0.00, 0.10) | 0.49 (0.37, 0.60) | NA (NA, NA) | 0.49 (0.36, 0.61) |
| Victivallis | 0.39 (0.26, 0.52) | 0.06 (0.02, 0.20) | 0.94 (0.81, 0.98) | 0.50 (0.15, 0.85) | 0.52 (0.40, 0.63) | 0.51 (0.39, 0.64) |
| Cerasicoccus | 0.50 (0.50, 0.50) | 1.00 (0.90, 1.00) | 0.00 (0.00, 0.10) | 0.49 (0.37, 0.60) | NA (NA, NA) | 0.51 (0.39, 0.64) |
| Puniceicoccus | 0.49 (0.46, 0.51) | 1.00 (0.90, 1.00) | 0.00 (0.00, 0.10) | 0.49 (0.37, 0.60) | NA (NA, NA) | 0.49 (0.36, 0.61) |
| Uncultured-24 | 0.46 (0.35, 0.56) | 1.00 (0.90, 1.00) | 0.00 (0.00, 0.10) | 0.49 (0.37, 0.60) | NA (NA, NA) | 0.49 (0.36, 0.61) |
| Akkermansia | 0.48 (0.34, 0.62) | 0.27 (0.15, 0.44) | 0.83 (0.67, 0.92) | 0.60 (0.36, 0.80) | 0.55 (0.41, 0.67) | 0.53 (0.40, 0.65) |
| Pycnora | 0.50 (0.50, 0.50) | 1.00 (0.90, 1.00) | 0.00 (0.00, 0.10) | 0.49 (0.37, 0.60) | NA (NA, NA) | 0.51 (0.39, 0.64) |
| {Unknown Genus}R Xylariaceae | 0.50 (0.50, 0.50) | 1.00 (0.90, 1.00) | 0.00 (0.00, 0.10) | 0.49 (0.37, 0.60) | NA (NA, NA) | 0.51 (0.39, 0.64) |
| Blastocystis | 0.46 (0.40, 0.52) | 1.00 (0.90, 1.00) | 0.00 (0.00, 0.10) | 0.49 (0.37, 0.60) | NA (NA, NA) | 0.49 (0.36, 0.61) |
