# Supplementary material for: Fecal profiling reveals a common microbial signature for pancreatic cancer in Finnish and Iranian cohorts
Source: Gut Pathog. 2025 Apr 16;17:24. doi: 10.1186/s13099-025-00698-0 (PMC12001732; doi:10.1186/s13099-025-00698-0)
Supplement: Supplementary file 9 — Additional file 9: Table S7. ROC curve analysis for PDAC prediction performance metrics. ROC curve analysis for PDAC prediction performance metrics by taxonomic phylum-, family- and genus-based and machine learning methods using different feature selections methods [file 13099_2025_698_MOESM9_ESM.docx]

**Supplementary Table S7.** **ROC curve analysis for PDAC prediction performance metrics**

**Supplementary Table S7A.** ROC curve analysis for PDAC prediction performance metrics by taxonomic phylum-based and Machine Learning methods using different feature selections methods

| **Method** | **Variables** | **AUC (95% CI)** | **SE (95% CI)** | **SP (95% CI)** | **PPV (95% CI)** | **NPV (95% CI)** | **Accuracy 95% CI)** |
| --- | --- | --- | --- | --- | --- | --- | --- |
| Combination of Random Forest and Logistic Regression | All variables | 0.85 (0.74, 0.95) | 0.91 (0.76, 0.97) | 0.86 (0.71, 0.94) | 0.86 (0.71, 0.94) | 0.91 (0.76, 0.97) | 0.87 (0.76, 0.94) |
|  | LR-Selected variables | 0.83 (0.72, 0.93) | 0.82 (0.66, 0.91) | 0.80 (0.64, 0.90) | 0.79 (0.63, 0.90) | 0.82 (0.66, 0.92) | 0.79 (0.68, 0.88) |
|  | SVM - Selected variables | 0.70 (0.57, 0.82) | 0.76 (0.59, 0.87) | 0.63 (0.46, 0.77) | 0.66 (0.50, 0.79) | 0.73 (0.56, 0.86) | 0.68 (0.55, 0.78) |
|  | NB - Selected variables | 0.80 (0.68, 0.91) | 0.85 (0.69, 0.93) | 0.74 (0.58, 0.86) | 0.76 (0.60, 0.87) | 0.84 (0.67, 0.93) | 0.79 (0.68, 0.88) |
|  | NN - Selected variables | 0.66 (0.52, 0.79) | 0.67 (0.50, 0.80) | 0.69 (0.52, 0.81) | 0.67 (0.50, 0.80) | 0.69 (0.52, 0.81) | 0.68 (0.55, 0.78) |
|  | DT - Selected variables | 0.64 (0.54, 0.74) | 0.91 (0.76, 0.97) | 0.37 (0.23, 0.54) | 0.58 (0.44, 0.70) | 0.81 (0.57, 0.93) | 0.51 (0.39, 0.64) |
| Random Forest Method | All variables | 0.85 (0.74, 0.95) | 0.91 (0.76, 0.97) | 0.86 (0.71, 0.94) | 0.86 (0.71, 0.94) | 0.91 (0.76, 0.97) | 0.87 (0.76, 0.94) |
|  | LR-Selected variables | 0.83 (0.72, 0.93) | 0.82 (0.66, 0.91) | 0.80 (0.64, 0.90) | 0.79 (0.63, 0.90) | 0.82 (0.66, 0.92) | 0.79 (0.68, 0.88) |
|  | SVM - Selected variables | 0.70 (0.57, 0.82) | 0.76 (0.59, 0.87) | 0.63 (0.46, 0.77) | 0.66 (0.50, 0.79) | 0.73 (0.56, 0.86) | 0.68 (0.55, 0.78) |
|  | NB - Selected variables | 0.80 (0.68, 0.91) | 0.85 (0.69, 0.93) | 0.74 (0.58, 0.86) | 0.76 (0.60, 0.87) | 0.84 (0.67, 0.93) | 0.79 (0.68, 0.88) |
|  | NN - Selected variables | 0.66 (0.52, 0.79) | 0.67 (0.50, 0.80) | 0.69 (0.52, 0.81) | 0.67 (0.50, 0.80) | 0.69 (0.52, 0.81) | 0.68 (0.55, 0.78) |
|  | DT - Selected variables | 0.64 (0.54, 0.74) | 0.91 (0.76, 0.97) | 0.37 (0.23, 0.54) | 0.58 (0.44, 0.70) | 0.81 (0.57, 0.93) | 0.51 (0.39, 0.64) |
| Logistic Regression | All variables | 0.85 (0.74, 0.95) | 0.91 (0.76, 0.97) | 0.86 (0.71, 0.94) | 0.86 (0.71, 0.94) | 0.91 (0.76, 0.97) | 0.87 (0.76, 0.94) |
|  | LR-Selected variables | 0.79 (0.68, 0.91) | 0.82 (0.66, 0.91) | 0.80 (0.64, 0.90) | 0.79 (0.63, 0.90) | 0.82 (0.66, 0.92) | 0.79 (0.68, 0.88) |
|  | SVM - Selected variables | 0.72 (0.60, 0.85) | 0.85 (0.69, 0.93) | 0.66 (0.49, 0.79) | 0.70 (0.55, 0.82) | 0.82 (0.64, 0.92) | 0.74 (0.61, 0.83) |
|  | NB - Selected variables | 0.79 (0.68, 0.91) | 0.91 (0.76, 0.97) | 0.71 (0.55, 0.84) | 0.75 (0.60, 0.86) | 0.89 (0.73, 0.96) | 0.81 (0.70, 0.89) |
|  | NN - Selected variables | 0.66 (0.52, 0.79) | 0.67 (0.50, 0.80) | 0.69 (0.52, 0.81) | 0.67 (0.50, 0.80) | 0.69 (0.52, 0.81) | 0.68 (0.55, 0.78) |
|  | DT - Selected variables | 0.65 (0.53, 0.76) | 0.85 (0.69, 0.93) | 0.49 (0.33, 0.64) | 0.61 (0.46, 0.74) | 0.77 (0.57, 0.90) | 0.66 (0.54, 0.77) |

**Table S7A** provides a summary of the ROC curve analysis conducted to evaluate the prediction of pancreatic ductal adenocarcinoma (PDAC) versus healthy controls using various machine learning methods and taxonomic feature selection approaches. Performance metrics, including Area Under the Curve (AUC), sensitivity (SE), specificity (SP), positive predictive value (PPV), negative predictive value (NPV), and accuracy, are presented with their corresponding 95% confidence intervals (CIs). Features were selected through Random Forest (*Firmicutes*, *Proteobacteria*, *Verrucomicrobiota*, *Bacteroidota*, *{Unknown Phylum} Bacteria*, *Actinobacteriota*, *Desulfobacterota*, and *Cyanobacteria*), Logistic Regression (*Firmicutes*, *Bacteroidota*, and *Cyanobacteria*), and a combined approach including unique features from both methods. Various machine learning methods were applied, including Random Forest, Logistic Regression, Support Vector Machines (SVM), Naive Bayes (NB), Neural Networks (NN), and Decision Trees (DT).

**Supplementary Table S7B.** ROC curve analysis for PDAC prediction performance metrics by taxonomic family-based and Machine Learning methods using different feature selections methods

| **Method** | **Variables** | **AUC (95% CI)** | **SE (95% CI)** | **SP (95% CI)** | **PPV (95% CI)** | **NPV (95% CI)** | **Accuracy 95% CI)** |
| --- | --- | --- | --- | --- | --- | --- | --- |
| Combination of Random Forest and Logistic Regression | All variables | 0.50 (0.38, 0.63) | 0.64 (0.47, 0.78) | 0.40 (0.26, 0.56) | 0.50 (0.36, 0.64) | 0.54 (0.35, 0.71) | 0.51 (0.39, 0.64) |
|  | LR-Selected variables | 0.66 (0.53, 0.79) | 0.79 (0.62, 0.89) | 0.54 (0.38, 0.70) | 0.62 (0.47, 0.75) | 0.73 (0.54, 0.86) | 0.66 (0.54, 0.77) |
|  | SVM - Selected variables | 0.72 (0.60, 0.85) | 0.70 (0.53, 0.83) | 0.69 (0.52, 0.81) | 0.68 (0.51, 0.81) | 0.71 (0.54, 0.83) | 0.69 (0.57, 0.80) |
|  | NB - Selected variables | 0.63 (0.49, 0.77) | 0.55 (0.38, 0.70) | 0.86 (0.71, 0.94) | 0.78 (0.58, 0.90) | 0.67 (0.52, 0.79) | 0.71 (0.58, 0.81) |
|  | NN - Selected variables | 0.83 (0.73, 0.93) | 0.73 (0.56, 0.85) | 0.86 (0.71, 0.94) | 0.83 (0.65, 0.92) | 0.77 (0.62, 0.87) | 0.79 (0.68, 0.88) |
|  | DT - Selected variables | 0.60 (0.48, 0.72) | 0.64 (0.47, 0.78) | 0.57 (0.41, 0.72) | 0.58 (0.42, 0.73) | 0.63 (0.45, 0.77) | 0.51 (0.39, 0.64) |
| Random Forest Method | All variables | 0.50 (0.38, 0.63) | 0.64 (0.47, 0.78) | 0.40 (0.26, 0.56) | 0.50 (0.36, 0.64) | 0.54 (0.35, 0.71) | 0.51 (0.39, 0.64) |
|  | LR-Selected variables | 0.72 (0.59, 0.84) | 0.70 (0.53, 0.83) | 0.71 (0.55, 0.84) | 0.70 (0.53, 0.83) | 0.71 (0.55, 0.84) | 0.69 (0.57, 0.80) |
|  | SVM - Selected variables | 0.73 (0.61, 0.85) | 0.70 (0.53, 0.83) | 0.69 (0.52, 0.81) | 0.68 (0.51, 0.81) | 0.71 (0.54, 0.83) | 0.68 (0.55, 0.78) |
|  | NB - Selected variables | 0.59 (0.45, 0.73) | 0.39 (0.25, 0.56) | 0.89 (0.74, 0.95) | 0.76 (0.53, 0.90) | 0.61 (0.47, 0.73) | 0.65 (0.52, 0.76) |
|  | NN - Selected variables | 0.67 (0.54, 0.80) | 0.42 (0.27, 0.59) | 0.91 (0.78, 0.97) | 0.82 (0.59, 0.94) | 0.63 (0.49, 0.75) | 0.68 (0.55, 0.78) |
|  | DT - Selected variables | 0.60 (0.48, 0.72) | 0.64 (0.47, 0.78) | 0.57 (0.41, 0.72) | 0.58 (0.42, 0.73) | 0.63 (0.45, 0.77) | 0.51 (0.39, 0.64) |
| Logistic Regression | All variables | 0.50 (0.38, 0.63) | 0.64 (0.47, 0.78) | 0.40 (0.26, 0.56) | 0.50 (0.36, 0.64) | 0.54 (0.35, 0.71) | 0.51 (0.39, 0.64) |
|  | LR-Selected variables | 0.88 (0.78, 0.97) | 0.85 (0.69, 0.93) | 0.89 (0.74, 0.95) | 0.88 (0.72, 0.95) | 0.86 (0.71, 0.94) | 0.87 (0.76, 0.94) |
|  | SVM - Selected variables | 0.86 (0.77, 0.95) | 0.85 (0.69, 0.93) | 0.77 (0.61, 0.88) | 0.78 (0.62, 0.88) | 0.84 (0.68, 0.93) | 0.78 (0.66, 0.87) |
|  | NB - Selected variables | 0.81 (0.70, 0.92) | 0.91 (0.76, 0.97) | 0.66 (0.49, 0.79) | 0.71 (0.56, 0.83) | 0.88 (0.71, 0.96) | 0.78 (0.66, 0.87) |
|  | NN - Selected variables | 0.84 (0.74, 0.94) | 0.73 (0.56, 0.85) | 0.86 (0.71, 0.94) | 0.83 (0.65, 0.92) | 0.77 (0.62, 0.87) | 0.79 (0.68, 0.88) |
|  | DT - Selected variables | 0.77 (0.65, 0.88) | 0.85 (0.69, 0.93) | 0.74 (0.58, 0.86) | 0.76 (0.60, 0.87) | 0.84 (0.67, 0.93) | 0.79 (0.68, 0.88) |

**Table S7B** presents the ROC curve analysis conducted to evaluate the prediction of pancreatic ductal adenocarcinoma (PDAC) versus healthy controls using various machine learning methods and taxonomic family-based feature selection approaches. Performance metrics, including Area Under the Curve (AUC), sensitivity (SE), specificity (SP), positive predictive value (PPV), negative predictive value (NPV), and accuracy, are reported along with their corresponding 95% confidence intervals (CIs). Features were selected using Logistic Regression (*{Unknown Family} Clostridia UCG-014*, *Enterococcaceae*, *Prevotellaceae*, *Butyricicoccaceae*, *Enterobacteriaceae*, *Erysipelatoclostridiaceae*, *Muribaculaceae*, *{Unknown Family} RF39*, and *FamilyXI*) and Random Forest (*{Unknown Order} Clostridia*, *Ruminococcaceae*, *Streptococcaceae*, *Lachnospiraceae*, *{Unknown Family} Clostridia UCG-014*, *Peptostreptococcaceae*, *Lactobacillaceae*, *Enterobacteriaceae*, *{Unknown Order} Gammaproteobacteria*, *{Unknown Family} Oscillospirales*, and *Monoglobaceae*). A combined approach, incorporating unique features from both methods, was also used.

**Supplementary Table S7C.** ROC curve analysis for PDAC prediction performance metrics by taxonomic genus-based and Machine Learning methods using different feature selections methods

| **Method** | **Variables** | **AUC (95% CI)** | **SE (95% CI)** | **SP (95% CI)** | **PPV (95% CI)** | **NPV (95% CI)** | **Accuracy 95% CI)** |
| --- | --- | --- | --- | --- | --- | --- | --- |
| Combination of Random Forest and Logistic Regression | All variables | 0.42 (0.30, 0.54) | 1.00 (0.90, 1.00) | 0.00 (0.00, 0.10) | 0.49 (0.37, 0.60) | NA (NA, NA) | 0.49 (0.36, 0.61) |
|  | LR-Selected variables | 0.61 (0.51, 0.70) | 0.88 (0.73, 0.95) | 0.34 (0.21, 0.51) | 0.56 (0.42, 0.68) | 0.75 (0.51, 0.90) | 0.51 (0.39, 0.64) |
|  | SVM - Selected variables | 0.84 (0.75, 0.93) | 0.76 (0.59, 0.87) | 0.80 (0.64, 0.90) | 0.78 (0.61, 0.89) | 0.78 (0.62, 0.88) | 0.78 (0.66, 0.87) |
|  | NB - Selected variables | 0.76 (0.63, 0.88) | 0.73 (0.56, 0.85) | 0.80 (0.64, 0.90) | 0.77 (0.60, 0.89) | 0.76 (0.60, 0.87) | 0.76 (0.65, 0.86) |
|  | NN - Selected variables | 0.83 (0.73, 0.93) | 0.73 (0.56, 0.85) | 0.86 (0.71, 0.94) | 0.83 (0.65, 0.92) | 0.77 (0.62, 0.87) | 0.79 (0.68, 0.88) |
|  | DT - Selected variables | 0.59 (0.46, 0.73) | 0.88 (0.73, 0.95) | 0.37 (0.23, 0.54) | 0.57 (0.43, 0.69) | 0.76 (0.53, 0.90) | 0.62 (0.49, 0.73) |
| Random Forest Method | All variables | 0.42 (0.30, 0.54) | 1.00 (0.90, 1.00) | 0.00 (0.00, 0.10) | 0.49 (0.37, 0.60) | NA (NA, NA) | 0.49 (0.36, 0.61) |
|  | LR-Selected variables | 0.76 (0.64, 0.88) | 0.79 (0.62, 0.89) | 0.71 (0.55, 0.84) | 0.72 (0.56, 0.84) | 0.78 (0.61, 0.89) | 0.75 (0.63, 0.85) |
|  | SVM - Selected variables | 0.80 (0.69, 0.90) | 0.82 (0.66, 0.91) | 0.71 (0.55, 0.84) | 0.73 (0.57, 0.85) | 0.81 (0.64, 0.91) | 0.76 (0.65, 0.86) |
|  | NB - Selected variables | 0.77 (0.66, 0.88) | 0.76 (0.59, 0.87) | 0.71 (0.55, 0.84) | 0.71 (0.55, 0.84) | 0.76 (0.59, 0.87) | 0.74 (0.61, 0.83) |
|  | NN - Selected variables | 0.67 (0.54, 0.80) | 0.42 (0.27, 0.59) | 0.91 (0.78, 0.97) | 0.82 (0.59, 0.94) | 0.63 (0.49, 0.75) | 0.68 (0.55, 0.78) |
|  | DT - Selected variables | 0.60 (0.49, 0.72) | 0.64 (0.47, 0.78) | 0.57 (0.41, 0.72) | 0.58 (0.42, 0.73) | 0.63 (0.45, 0.77) | 0.51 (0.39, 0.64) |
| Logistic Regression | All variables | 0.42 (0.30, 0.54) | 1.00 (0.90, 1.00) | 0.00 (0.00, 0.10) | 0.49 (0.37, 0.60) | NA (NA, NA) | 0.49 (0.36, 0.61) |
|  | LR-Selected variables | 0.82 (0.71, 0.93) | 0.82 (0.66, 0.91) | 0.83 (0.67, 0.92) | 0.82 (0.66, 0.91) | 0.83 (0.67, 0.92) | 0.81 (0.70, 0.89) |
|  | SVM - Selected variables | 0.87 (0.78, 0.95) | 0.79 (0.62, 0.89) | 0.80 (0.64, 0.90) | 0.79 (0.62, 0.89) | 0.80 (0.64, 0.90) | 0.79 (0.68, 0.88) |
|  | NB - Selected variables | 0.75 (0.62, 0.88) | 0.76 (0.59, 0.87) | 0.80 (0.64, 0.90) | 0.78 (0.61, 0.89) | 0.78 (0.62, 0.88) | 0.78 (0.66, 0.87) |
|  | NN - Selected variables | 0.83 (0.73, 0.93) | 0.73 (0.56, 0.85) | 0.86 (0.71, 0.94) | 0.83 (0.65, 0.92) | 0.77 (0.62, 0.87) | 0.79 (0.68, 0.88) |
|  | DT - Selected variables | 0.85 (0.75, 0.94) | 0.82 (0.66, 0.91) | 0.83 (0.67, 0.92) | 0.82 (0.66, 0.91) | 0.83 (0.67, 0.92) | 0.82 (0.71, 0.91) |

**Table S7C** summarizes the ROC curve analysis conducted to evaluate the prediction of pancreatic ductal adenocarcinoma (PDAC) versus healthy controls using various machine learning methods and taxonomic genus-based feature selection approaches. Performance metrics, including Area Under the Curve (AUC), sensitivity (SE), specificity (SP), positive predictive value (PPV), negative predictive value (NPV), and accuracy, are presented along with their corresponding 95% confidence intervals (CIs). Features were selected using Logistic Regression (*Unknown Family Clostridia UCG-014*, *Anaerostipes*, *Erysipelotrichaceae UCG-003*, *Lachnospiraceae UCG-001*, *Unknown Genus Muribaculaceae*, *Prevotella_9*, *Agathobacter*, *Enterococcus*, *Hungatella*, *Intestinimonas*, *Enterobacter*, *Uncultured11*, *Butyricicoccus*, *Unknown Family RF39*, *Citrobacter*, *Lachnospiraceae NK4A136 group*, *Uncultured03*, *Klebsiella*, *Eubacterium xylanophilum group*, and *Romboutsia*) and Random Forest (*Unknown Order Clostridia*, *Unknown Genus Streptococcaceae*, *Limosilactobacillus*, *Ruminococcus*, *Eubacterium xylanophilum group*, *Streptococcus*, *Anaerostipes*, *Unknown Genus Lactobacillaceae*, *Agathobacter*, *Coprococcus*, *Lachnospiraceae NC2004 group*, *Ruminococcus gauvreauii group*, and *Lachnospiraceae FCS020 group*). A combined approach, incorporating unique features from both methods, was also applied. Machine learning methods included Random Forest, Logistic Regression, Support Vector Machines (SVM), Naive Bayes (NB), Neural Networks (NN), and Decision Trees (DT).
